# Drainage basin checklists and dichotomous keys for inland fishes of Texas

**DOI:** 10.3897/zookeys.874.35618

**Published:** 2019-09-02

**Authors:** Cody Andrew Craig, Timothy Hallman Bonner

**Affiliations:** 1 Department of Biology/Aquatic Station, Texas State University, San Marcos, Texas 78666, USA Texas State University San Marcos United States of America

**Keywords:** Texas, fish, checklist, dichotomous key, key, identification, occurrence, drainage

## Abstract

Species checklists and dichotomous keys are valuable tools that provide many services for ecological studies and management through tracking native and non-native species through time. We developed nine drainage basin checklists and dichotomous keys for 196 inland fishes of Texas, consisting of 171 native fishes and 25 non-native fishes. Our checklists were updated from previous checklists and revised using reports of new established native and non-native fishes in Texas, reports of new fish occurrences among drainages, and changes in species taxonomic nomenclature. We provided the first dichotomous keys for major drainage basins in Texas. Among the 171 native inland fishes, 6 species are considered extinct or extirpated, 13 species are listed as threatened or endangered by U.S. Fish and Wildlife Service, and 59 species are listed as Species of Greatest Conservation Need (SGCN) by the state of Texas. Red River drainage basin was the most speciose with 120 fishes. Rio Grande & Pecos drainage basin had the greatest number of threatened or endangered fishes (N = 7) and the greatest number of SGCN fishes (N = 28). We revised drainage basin occurrences for 77 species. Drainage basin checklists and dichotomous keys provide finer resolution of species distributions within the geopolitical boundaries of Texas and can reduce probability of errors in fish identification errors by removing species not occurring within a natural boundary.

## Introduction

Species checklists consolidate biodiversity records using standardized taxonomic nomenclature and updated species occurrences within pre-defined boundaries (Fleishman et al. 2006; Martellos and Nimis 2015). Benefits of checklists include use in ecological studies and natural resources management, such as assessments of global patterns in species richness (Gaston 2000), identification of biodiversity hotspots (Kent et al. 2002), occurrences for species distribution models (Caicco et al. 1995), and expansion and contraction of native and non-native species (Lee et al. 2008; Magurran et al. 2010). Often coupled with checklists, dichotomous keys facilitate species identification using a series of distinguishing characteristics (Griffing 2011). Dichotomous keys usually are created for taxa within geopolitical boundaries (e.g., Hubbs et al. 2008); however, geopolitical boundaries often are arbitrary to species distributions (Forman 2014). Recent development and use of dichotomous keys along natural boundaries, such as drainage basin (Worsham et al. 2016), provide finer resolution on species distributions and reduce probability of identification errors by removing species not occurring within a natural boundary.

Within Texas, Evermann and Kendall (1894) published the first checklist of freshwater fishes. A revised checklist was published by Baughman (1950a, 1950b), using standardized taxonomic nomenclature provided by Jordan et al. (1930). Jurgens and Hubbs (1953) were the first to publish a checklist using standardized taxonomic nomenclature provided by American Fisheries Society Committee on Names of Fishes (Chute et al. 1948). This checklist was periodically revised by Hubbs (i.e., Hubbs 1957, 1958, 1961, 1972, 1976, 1982). Knapp (1953) published a checklist and the first dichotomous key for freshwater fishes of Texas. Texas drainage basin checklists were published for western Gulf Slope drainage basins (Conner and Suttkus 1986), Mississippi River drainage basins (Cross et al. 1986), and Rio Grande drainage basin (Smith and Miller 1986). Statewide checklist and dichotomous key were revised by Hubbs et al. (1991) and Hubbs et al. (2008).

Revisions of checklists for freshwater fishes of Texas were necessary through time to accommodate additions of previously unreported species, multiple species described from a single species, and non-native species introductions (Hanks and McCoid 1988; Eisenhour 2004; Gallaway et al. 2008) and to accommodate removal of introduced fishes that did not establish populations (Howells 2001). In addition, species distributions were updated to document range expansions (e.g., *Percina carbonaria*, Hubbs et al. 2008), range contractions (e.g., *Ictalurus lupus*, Kelsch and Hendricks 1990), and name changes (e.g., *Micropterus treculi* to *Micropterus treculii*) using standardized taxonomic nomenclature (e.g., Nelson et al. 2004). Since Hubbs et al. (2008), American Fisheries Society and American Society of Ichthyologists and Herpetologists (AFS-ASIH) Committee of Names of Fishes published a revised common and scientific names list (Page et al. 2013), new native species were reported within Texas (e.g., Craig et al. 2015), a fish name was synonymized (Echelle et al. 2013), introduced species became established (e.g., Cohen et al. 2014), and species ranges expanded (e.g., Dautreuil et al. 2016) and contracted (e.g., Craig et al. 2017).

Purposes of this paper were to develop drainage basin checklists and dichotomous keys for Texas freshwater fishes. As with previous revisions, we updated the statewide checklist and dichotomous key with new species, removal of species, and range changes. However, our checklists and dichotomous keys differ markedly from previous revisions. We identified fishes as inland, rather than freshwater, and divided the geopolitical boundary into natural boundaries using major drainage basins. Texas is particularly well suited for drainage basin checklists and keys because majority of the drainage basins became independent of one another during the early Holocene (i.e., river termini in Gulf of Mexico bays), generally restricting freshwater fish movement among drainage basins. As such, fishes are rarely homogenously distributed among all drainage basins, with 41% of fishes restricted to one or two drainage basins (Conner and Suttkus 1986; Hubbs et al. 2008).

## Materials and methods

Development of a freshwater fish checklist is a challenge within natural or geopolitical boundaries having fresh and marine environments (Ross 2001; Moyle 2002). Inclusions of marine fishes on a freshwater fish checklist are subjective (Ross 2001). Knapp (1953) included marine fishes if observed in waters with salinities < 2 ppt. Hubbs et al. (1991) included marine fishes if found in “low salinity habitats”. Using salinity as an objective measure is limiting. Several fishes found in upper reaches of the Canadian River, Red River, Brazos River, Colorado River, and Pecos River inhabit saline waters with salinities exceeding 50 ppt at times (Echelle et al. 1972), so excluding fishes based on salinity tolerances would exclude several species not known to inhabit marine or estuarine environments. Avoiding salinity as a measure, we used the term “inland” instead of “freshwater” to represent fishes found in Texas rivers generally upstream from transitory freshwater-saltwater boundaries. We accepted fishes as inland if they hatch, feed, and reproduce within inland waters (i.e., all water bodies upstream of river termini). We also accepted two forms of marine fishes as inland fishes: diadromous fishes (i.e., *Anguilla rostrata*, *Agonostomus monticola*, and *Trinectes maculatus*) and fishes with reported self-sustaining populations within inland waters (e.g., *Syngnathus scovelli*, Martin et al. 2013). Our acceptance of fishes as inland oversimplifies the complex and dynamic relationship of fish communities within estuarine systems of the Gulf of Mexico (Gelwick et al. 2001); therefore, our inland fish checklists underestimate the number of fishes encountered in estuarine systems.

**Figure 1.**
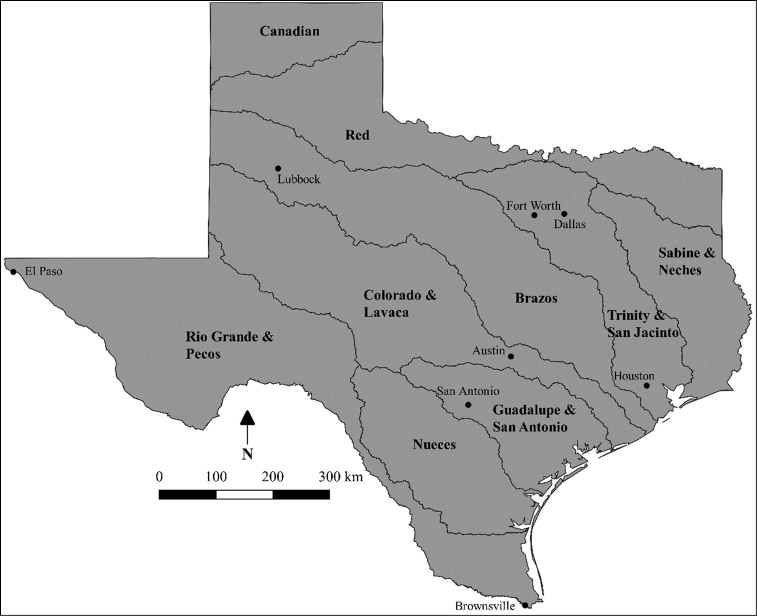
Map of Texas with major drainage basins outlined and labeled. Also included are major cities to serve as reference points.

Drainage basins were defined as major independent rivers that flow directly into the Gulf of Mexico (i.e., Sabine & Neches, Trinity & San Jacinto, Brazos, Colorado & Lavaca, Guadalupe & San Antonio, Nueces, and Rio Grande & Pecos) or beyond Texas borders (i.e., Canadian and Red) (Figure 1). Drainage basin checklists were developed using specific (Conner and Suttkus 1986; Cross et al. 1986; Smith and Miller 1986) and generalized (Hubbs et al. 2008) drainage basin checklists. Checklists were consolidated and updated based on drainage basin distribution records for each species using Texas Natural History Collections database (Hendrickson and Cohen 2015), published consolidated species accounts (e.g., Lee et al. 1980), and published individual species range accounts (e.g., Wilde and Bonner 2000). We only included species from previous checklists if species were recognized by Page et al. (2013) to minimize taxonomic inflation (Isaac et al. 2004). New species were added to checklists and keys based on published accounts of self-sustaining populations (*Ameiurus nebulosus*; Craig et al. 2015). A species was designated as native if it occurs within at least one Texas drainage basin without human aid. Transient border species (i.e., *Pimephales notatus*, Lee and Shute 1980; *Hiodon tergisus*, Gilbert 1980; *Cyprinella panarcys*, Pinion et al. 2018) with occurrences in boundary waters of Texas were excluded because of uncertainty in self-sustaining populations. At least 80 non-native fishes have been introduced into Texas drainage basins; however, the majority did not establish self-sustaining populations (Howells 2001). Non-native fishes were included in drainage basin checklists if we had evidence (i.e., publications, personal communications) of self-sustaining populations or regular stocking (e.g., *Ctenopharyngodon idella*). Fishes considered extinct (IUCN 2018) were included in the checklist but excluded from keys because of low likelihood of encounter.

Each drainage basin dichotomous key consists of family and species keys. We developed novel distinguishing characteristics for family and species keys along with modifying and using characteristics from original species descriptions (e.g., Eisenhour 2004) and existing keys (e.g., Robison and Buchanan 1988; Sublette et al. 1990; Boschung and Mayden 2004; Thomas et al. 2007; Hubbs et al. 2008). Distinguishing characteristics were comprised of external and internal morphologies, meristics, and color patterns of adult fishes. Each couplet lists the most pronounced distinguishing characteristic first, followed by additional, generally less pronounced, distinguishing characteristics.

## Results and discussion

The composite drainage basin checklist included 196 inland fishes, representing 79 genera and 30 families (Table 1). Dichotomous keys were developed for nine drainage basins (Suppl. material 1). The number of inland fishes, based on our definition herein, reported in previous checklists ranged from 93 (Evermann and Kendall 1894) to 191 (Hubbs et al. 2008). Hubbs et al. (2008) and our composite drainage basin checklist were the most similar but with differences. Our checklist included three fishes reported in Texas after 2008: native *Ameiurus nebulosus* (Craig et al. 2015), non-native *Xiphophorus variatus* (Cohen et al. 2014), and non-native *Hypophthalmichthys nobilis* (T. Bister, Texas Parks and Wildlife Inland Fisheries, personal communication 10 March 2019). Fishes included by Hubbs et al. (2008) and excluded from our checklist were *Cyprinella* sp., *Cycleptus* sp., and *Ictalurus* sp., because Page et al. (2013) did not recognize these three putative species. Also based on Page et al. (2013), fish names were changed for three species: *Herichthys cyanoguttatus*, *Erimyzon claviformis*, and *Menidia audens*. One species (i.e., *Gambusia clarkhubbsi*) was included by Hubbs et al. (2008) and Page et al. (2013) but excluded from our checklist, because *G. clarkhubbsi* was later determined to be a junior synonym for *Gambusia krumholzi* (Echelle et al. 2013). *Gambusia krumholzi* replaced *G. clarkhubbsi* in our checklist. We excluded 8 non-native fishes reported by Hubbs et al. (2008), each lacking evidence of self-sustaining populations: *Scardinius erythrophthalmus*, *Agamyxis pectinifrons*, *Platydoras armatulus*, *Pterygoplichthys multiradiatus*, *Esox lucius*, *Perca flavescens*, *Sander canadensis*, and *Tilapia zillii*. Our checklist includes updated distributions of several fishes from previous checklists. Our checklist has 77 fishes with different drainage basin distributions compared to the drainage basin checklists of Conner and Suttkus (1986), Cross et al. (1986), and Smith and Miller (1986). Although interpreted from generalized descriptions, we determined our checklist has different drainage basin distributions of at least 46 fishes compared to Hubbs et al. (2008). Differences in distributions of fishes are largely due to the generalized nature of Hubbs et al. (2008) descriptions, but also include range expansions and contractions.

**Table 1.** Fishes in Texas inland waters. Presence is denoted by “X”. All scientific and common names were from Page et al. (2013). Asterisk next to scientific name denotes species that were not included in the dichotomous keys due to low likelihood of encounter. “Native” denotes species is native to any Texas drainage basin. “Ext/exp” denotes species is extinct or extirpated from Texas. “USFWS” denotes species that are federally listed as Threatened or Endangered Species by United States Fish and Wildlife Service. “SGCN” denotes species that are state listed as Species of Greatest Conservation Need.

	Family	Species	Common Name	Native	Ext/exp	USFWS	SGCN	Canadian	Red	Sabine & Neches	Trinity & San Jacinto	Brazos	Colorado & Lavaca	Guadalupe & San Antonio	Nueces	Rio Grande & Pecos
1	Petromyzontidae	*Ichthyomyzon castaneus*	Chestnut Lamprey	X					X	X						
2		*Ichthyomyzon gagei*	Southern Brook Lamprey	X					X	X	X					
3	Acipenseridae	*Scaphirhynchus platorynchus*	Shovelnose Sturgeon	X			X		X							
4	Polyodontidae	*Polyodon spathula*	Paddlefish	X			X		X	X	X					
5	Lepisosteidae	*Atractosteus spatula*	Alligator Gar	X			X		X	X	X	X	X	X	X	X
6		*Lepisosteus oculatus*	Spotted Gar	X					X	X	X	X	X	X	X	X
7		*Lepisosteus osseus*	Longnose Gar	X					X	X	X	X	X	X	X	X
8		*Lepisosteus platostomus*	Shortnose Gar	X					X							
9	Amiidae	*Amia calva*	Bowfin	X					X	X	X	X	X			
10	Hiodontidae	*Hiodon alosoides*	Goldeye	X			X		X							
11	Anguillidae	*Anguilla rostrata*	American Eel	X			X		X	X	X	X	X	X	X	X
12	Clupeidae	*Dorosoma cepedianum*	Gizzard Shad	X				X	X	X	X	X	X	X	X	X
13		*Dorosoma petenense*	Threadfin Shad	X				X	X	X	X	X	X	X	X	X
14	Cyprinidae	*Campostoma anomalum*	Central Stoneroller	X				X	X		X	X	X	X	X	X
15		*Campostoma ornatum*	Mexican Stoneroller	X			X									X
16		*Carassius auratus*	Goldfish						X	X	X	X	X	X	X	X
17		*Ctenopharyngodon idella*	Grass Carp					X	X	X	X	X	X	X	X	X
18		*Cyprinella lepida*	Plateau Shiner	X			X							X	X	
19		*Cyprinella lutrensis*	Red Shiner	X				X	X	X	X	X	X	X	X	X
20		*Cyprinella proserpina*	Proserpine Shiner	X			X									X
21		*Cyprinella venusta*	Blacktail Shiner	X					X	X	X	X	X	X	X	X
22		*Cyprinus carpio*	Common carp					X	X	X	X	X	X	X	X	X
23		*Dionda argentosa*	Manantial Roundnose Minnow	X			X									X
24		*Dionda diaboli*	Devils River Minnow	X		X	X									X
25		*Dionda episcopa*	Roundnose Minnow	X			X									X
26		*Dionda nigrotaeniata*	Guadalupe Roundnose Minnow	X			X						X	X		
27		*Dionda serena*	Nueces Roundnose Minnow	X			X								X	
28		*Gila pandora*	Rio Grande Chub	X			X									X
29		*Hybognathus amarus*	Rio Grande Silvery Minnow	X		X	X									X
30		*Hybognathus hayi*	Cypress Minnow	X					X	X						
31		*Hybognathus nuchalis*	Mississippi Silvery Minnow	X					X	X	X	X				
32		*Hybognathus placitus*	Plains Minnow	X				X	X			X	X			
33		*Hybopsis amnis*	Pallid Shiner	X						X	X	X	X	X		
34		*Hypophthalmichthys nobilis*	Bighead Carp						X							
35		*Luxilus chrysocephalus*	Striped Shiner	X					X							
36		*Lythrurus fumeus*	Ribbon Shiner	X					X	X	X	X	X	X		
37		*Lythrurus umbratilis*	Redfin Shiner	X					X	X	X					
38	Cyprinidae	*Macrhybopsis aestivalis*	Speckled Chub	X			X									X
39		*Macrhybopsis australis*	Prairie Chub	X			X		X							
40		*Macrhybopsis hyostoma*	Shoal Chub	X					X	X	X	X	X			
41		*Macrhybopsis marconis*	Burrhead Chub	X									X	X		
42		*Macrhybopsis storeriana*	Silver Chub	X			X		X			X				
43		*Macrhybopsis tetranema*	Peppered Chub	X			X	X								
44		*Notemigonus crysoleucas*	Golden Shiner	X				X	X	X	X	X	X	X	X	X
45		*Notropis amabilis*	Texas Shiner	X			X						X	X	X	X
46		*Notropis atherinoides*	Emerald Shiner	X				X	X	X	X					
47		*Notropis atrocaudalis*	Blackspot Shiner	X			X		X	X	X	X				
48		*Notropis bairdi*	Red River Shiner	X			X		X							
49		*Notropis blennius*	River Shiner	X				X	X							
50		*Notropis braytoni*	Tamaulipas Shiner	X			X									X
51		*Notropis buccula*	Smalleye Shiner	X		X	X					X	X			
52		*Notropis buchanani*	Ghost Shiner	X					X	X	X	X	X	X	X	X
53		*Notropis chalybaeus*	Ironcolor Shiner	X			X		X	X	X			X		
54		*Notropis chihuahua*	Chihuahua Shiner	X			X									X
55		*Notropis girardi*	Arkansas River Shiner	X		X	X	X								
56		*Notropis jemezanus*	Rio Grande Shiner	X			X									X
57		*Notropis maculatus*	Taillight Shiner	X			X		X							
58		Notropis orca*	Phantom Shiner	X	X											X
59		*Notropis oxyrhynchus*	Sharpnose Shiner	X		X	X					X	X			
60		*Notropis potteri*	Chub Shiner	X			X		X		X	X				
61		*Notropis sabinae*	Sabine Shiner	X			X			X	X					
62		*Notropis shumardi*	Silverband Shiner	X			X		X	X	X	X	X			
63		*Notropis simus*	Bluntnose Shiner	X	X	X	X									X
64		*Notropis stramineus*	Sand Shiner	X				X	X		X	X	X	X	X	X
65		*Notropis texanus*	Weed Shiner	X					X	X	X	X	X	X	X	
66		*Notropis volucellus*	Mimic Shiner	X					X	X	X	X	X	X	X	
67		*Opsopoeodus emiliae*	Pugnose Minnow	X					X	X	X	X	X	X	X	
68		*Phenacobius mirabilis*	Suckermouth Minnow	X				X	X	X	X		X			
69		*Pimephales promelas*	Fathead Minnow	X				X	X	X	X	X	X	X	X	X
70		*Pimephales vigilax*	Bullhead Minnow	X				X	X	X	X	X	X	X	X	X
71		*Platygobio gracilis*	Flathead Chub	X				X								
72		*Pteronotropis hubbsi*	Bluehead Shiner	X			X		X							
73		*Rhinichthys cataractae*	Longnose Dace	X			X									X
74		*Semotilus atromaculatus*	Creek Chub	X					X	X	X	X				
75	Catostomidae	*Carpiodes carpio*	River Carpsucker	X				X	X	X	X	X	X	X	X	X
76		*Cycleptus elongatus*	Blue Sucker	X			X		X	X	X	X	X	X	X	X
77		*Erimyzon claviformis*	Western Creek Chubsucker	X			X		X	X	X	X				
78		*Erimyzon sucetta*	Lake Chubsucker	X					X	X	X	X		X		
79		*Ictiobus bubalus*	Smallmouth Buffalo	X					X	X	X	X	X	X	X	X
80		*Ictiobus cyprinellus*	Bigmouth Buffalo	X					X	X						
81		*Ictiobus niger*	Black Buffalo	X					X	X		X	X			X
82		*Minytrema melanops*	Spotted Sucker	X					X	X	X	X	X			
83		*Moxostoma austrinum*	Mexican Redhorse	X			X									X
84		*Moxostoma congestum*	Gray Redhorse	X								X	X	X	X	X
85		*Moxostoma erythrurum*	Golden Redhorse	X					X							
86		*Moxostoma poecilurum*	Blacktail Redhorse	X						X	X					
87	Characidae	*Astyanax mexicanus*	Mexican Tetra	X					X		X	X	X	X	X	X
88	Ictaluridae	*Ameiurus melas*	Black Bullhead	X				X	X	X	X	X	X	X	X	X
89		*Ameiurus natalis*	Yellow Bullhead	X				X	X	X	X	X	X	X	X	X
90		*Ameiurus nebulosus*	Brown Bullhead	X					X							
91		*Ictalurus furcatus*	Blue Catfish	X					X	X	X	X	X	X	X	X
92		*Ictalurus lupus*	Headwater Catfish	X			X						X	X	X	X
93		*Ictalurus punctatus*	Channel Catfish	X				X	X	X	X	X	X	X	X	X
94		*Noturus gyrinus*	Tadpole Madtom	X					X	X	X	X	X	X	X	X
95		*Noturus nocturnus*	Freckled Madtom	X					X	X	X	X				
96		*Pylodictis olivaris*	Flathead Catfish	X				X	X	X	X	X	X	X	X	X
97		*Satan eurystomus*	Widemouth Blindcat	X			X							X		
98		*Trogloglanis pattersoni*	Toothless Blindcat	X			X							X		
99	Loricariidae	*Hypostomus plecostomus*	Suckermouth Catfish											X		X
100		*Pterygoplichthys anisitsi*	Southern Sailfin Catfish								X			X		
101		*Pterygoplichthys disjunctivus*	Vermiculated Sailfin Catfish								X			X		X
102	Salmonidae	*Oncorhynchus clarkii*	Cutthroat Trout	X	X		X									X
103		*Oncorhynchus mykiss*	Rainbow Trout					X	X	X	X	X	X	X	X	X
104	Esocidae	*Esox americanus*	Redfin Pickerel	X					X	X	X	X				
105		*Esox niger*	Chain Pickerel	X					X	X						
106	Aphredoderidae	*Aphredoderus sayanus*	Pirate Perch	X					X	X	X	X	X			
107	Mugilidae	*Mugil cephalus*	Striped Mullet	X					X	X	X	X	X	X	X	X
108		*Agonostomus monticola*	Mountain Mullet	X						X	X	X	X	X	X	X
109	Atherinopsidae	*Labidesthes sicculus*	Brook Silverside	X					X	X	X	X				
110		*Membras martinica*	Rough Silverside								X			X		X
111		*Menidia audens*	Mississippi Silverside	X				X	X	X	X	X	X	X	X	X
112	Fundulidae	*Fundulus blairae*	Western Starhead Topminnow	X					X	X	X	X				
113		*Fundulus chrysotus*	Golden Topminnow	X					X	X	X	X	X	X		
114		*Fundulus grandis*	Gulf Killifish						X			X	X			X
115		*Fundulus kansae*	Northern Plains Killifish	X				X								
116		*Fundulus notatus*	Blackstripe Topminnow	X					X	X	X	X	X	X	X	
117		*Fundulus olivaceus*	Blackspotted Topminnow	X					X	X	X	X				
118		*Fundulus zebrinus*	Plains Killifish	X					X		X	X	X			X
119		*Lucania goodei*	Bluefin Killifish											X		
120		*Lucania parva*	Rainwater Killifish	X									X	X		X
121	Cyprinodontidae	*Cyprinodon bovinus*	Leon Springs Pupfish	X		X	X									X
122		*Cyprinodon elegans*	Comanche Springs Pupfish	X		X	X									X
123		*Cyprinodon eximius*	Conchos Pupfish	X			X									X
124		*Cyprinodon pecosensis*	Pecos Pupfish	X			X									X
125		*Cyprinodon rubrofluviatilis*	Red River Pupfish	X			X	X	X			X	X			
126		*Cyprinodon variegatus*	Sheepshead Minnow								X	X	X	X		X
127	Poeciliidae	*Gambusia affinis*	Western Mosquitofish	X				X	X	X	X	X	X	X	X	X
128		Gambusia amistadensis*	Amistad Gambusia	X	X											X
129		*Gambusia gaigei*	Big Bend Gambusia	X		X	X									X
130		*Gambusia geiseri*	Largespring Gambusia	X									X	X		X
131		Gambusia georgei*	San Marcos Gambusia	X	X	X								X		
132		*Gambusia heterochir*	Clear Creek Gambusia	X		X	X						X			
133	Poeciliidae	*Gambusia krumholzi*	Spotfin Gambusia	X												X
134		*Gambusia nobilis*	Pecos Gambusia	X		X	X									X
135		*Gambusia senilis*	Blotched Gambusia	X	X		X									X
136		*Gambusia speciosa*	Tex-Mex Gambusia	X												X
137		*Heterandria formosa*	Least Killifish	X						X						
138		*Poecilia formosa*	Amazon Molly											X	X	X
139		*Poecilia latipinna*	Sailfin Molly							X	X	X	X	X	X	X
140		*Poecilia reticulata*	Guppy											X		
141		*Xiphophorus hellerii*	Green Swordtail											X		
142		*Xiphophorus variatus*	Variable Platyfish										X			
143	Syngnathidae	*Syngnathus scovelli*	Gulf Pipefish	X										X		
144	Moronidae	*Morone chrysops*	White Bass	X				X	X	X	X	X	X	X	X	X
145		*Morone mississippiensis*	Yellow Bass	X					X	X	X	X				
146		*Morone saxatilis*	Striped Bass						X	X	X	X	X	X	X	X
147	Centrarchidae	*Ambloplites rupestris*	Rock Bass											X		
148		*Centrarchus macropterus*	Flier	X					X	X	X					
149		*Lepomis auritus*	Redbreast Sunfish						X	X	X	X	X	X	X	X
150		*Lepomis cyanellus*	Green Sunfish	X				X	X	X	X	X	X	X	X	X
151		*Lepomis gulosus*	Warmouth	X					X	X	X	X	X	X	X	X
152		*Lepomis humilis*	Orangespotted Sunfish	X				X	X	X	X	X	X	X	X	
153		*Lepomis macrochirus*	Bluegill	X				X	X	X	X	X	X	X	X	X
154		*Lepomis marginatus*	Dollar Sunfish	X					X	X	X	X				
155		*Lepomis megalotis*	Longear Sunfish	X				X	X	X	X	X	X	X	X	X
156		*Lepomis microlophus*	Redear Sunfish	X				X	X	X	X	X	X	X	X	X
157		*Lepomis miniatus*	Redspotted Sunfish	X					X	X	X	X	X	X	X	X
158		*Lepomis symmetricus*	Bantam Sunfish	X					X	X	X	X	X			
159		*Micropterus dolomieu*	Smallmouth Bass					X	X			X	X	X	X	X
160		*Micropterus punctulatus*	Spotted Bass	X					X	X	X	X	X	X		
161		*Micropterus salmoides*	Largemouth Bass	X				X	X	X	X	X	X	X	X	X
162		*Micropterus treculii*	Guadalupe Bass	X			X					X	X	X	X	
163		*Pomoxis annularis*	White Crappie	X				X	X	X	X	X	X	X	X	X
164		*Pomoxis nigromaculatus*	Black Crappie	X					X	X	X	X	X	X	X	X
165	Percidae	*Ammocrypta clara*	Western Sand Darter	X			X		X	X						
166		*Ammocrypta vivax*	Scaly Sand Darter	X					X	X	X					
167		*Etheostoma artesiae*	Redspot Darter	X					X	X						
168		*Etheostoma asprigene*	Mud Darter	X					X	X						
169		*Etheostoma chlorosoma*	Bluntnose Darter	X					X	X	X	X	X	X		
170		*Etheostoma fonticola*	Fountain Darter	X		X	X							X		
171		*Etheostoma fusiforme*	Swamp Darter	X					X	X						
172		*Etheostoma gracile*	Slough Darter	X					X	X	X	X	X	X	X	X
173		*Etheostoma grahami*	Rio Grande Darter	X			X									X
174		*Etheostoma histrio*	Harlequin Darter	X					X	X						
175		*Etheostoma lepidum*	Greenthroat Darter	X									X	X	X	
176		*Etheostoma parvipinne*	Goldstripe Darter	X					X	X	X	X	X			
177		*Etheostoma proeliare*	Cypress Darter	X					X	X	X		X			
178		*Etheostoma radiosum*	Orangebelly Darter	X			X		X							
179		*Etheostoma spectabile*	Orangethroat Darter	X					X		X	X	X	X		
180		*Percina apristis*	Guadalupe Darter	X			X							X		
181		*Percina caprodes*	Logperch	X					X							
182		*Percina carbonaria*	Texas Logperch	X							X	X	X	X	X	
183		*Percina macrolepida*	Bigscale Logperch	X					X	X	X	X	X	X		X
184		*Percina maculata*	Blackside Darter	X			X		X	X	X					
185	Percidae	*Percina phoxocephala*	Slenderhead Darter	X					X							
186		*Percina sciera*	Dusky Darter	X					X	X	X	X	X			
187		*Percina shumardi*	River Darter	X					X	X				X		
188		*Sander vitreus*	Walleye					X								X
189	Sciaenidae	*Aplodinotus grunniens*	Freshwater Drum	X					X	X	X	X	X	X	X	X
190	Elassomatidae	*Elassoma zonatum*	Banded Pygmy Sunfish	X					X	X	X	X				
191	Cichlidae	*Herichthys cyanoguttatus*	Rio Grande Cichlid	X							X	X	X	X	X	X
192		*Oreochromis aureus*	Blue Tilapia								X	X	X	X	X	X
193		*Oreochromis mossambicus*	Mozambique Tilapia											X	X	X
194	Gobiidae	*Awaous banana*	River Goby	X												X
195		*Gobiosoma bosc*	Naked Goby										X			X
196	Achiridae	*Trinectes maculatus*	Hogchoker	X						X	X	X	X	X	X	
			Total	171	6	13	59	37	120	101	102	96	94	94	66	95

Our composite drainage basin checklist has 171 native and 25 non-native inland fishes. Among native species, three fishes (i.e., *Notropis orca*, *Gambusia amistadensis*, and *Gambusia georgei*) are considered extinct, and three fishes (i.e., *Notropis simus*, *Oncorhynchus clarkii*, and *Gambusia senilis*) are considered extirpated (Hubbs et al. 2008). Thirteen fishes are listed as threatened and endangered by U.S. Fish and Wildlife Service (USFWS), and 59 fishes are listed as Species of Greatest Conservation Need (SGCN, Texas Parks and Wildlife 2012). Number of native fishes by drainage basin ranged from 32 in the Canadian to 111 in the Red. Rio Grande & Pecos had the greatest number of USFWS threatened and endangered fishes (N = 7) and SGCN fishes (N = 28). Number of non-native fishes by drainage basin ranged from five in the Canadian to 20 in the Guadalupe & San Antonio. Origins of non-native fishes are from marine waters of Texas and from inland waters of North America and other continents (Table 2). Based on published accounts, non-native fishes were introduced for human consumption and sport (Nico and Fuller 1999), bait bucket releases (Howells 2001), vegetation control (Guillory and Gasaway 1978), accidental aquaculture releases (Howells 2001), and aquarium releases (Cohen et al. 2014).

**Table 2.** Non-native fishes established in Texas and their continent of origin with respective citation. Presence denoted by “X”.

Family	Species	Common Name	Marine	North America	Asia	Africa	South America	Europe	Citation
Cyprinidae	*Carassius auratus*	Goldfish			X				Hubbs et al. 2008
	*Ctenopharyngodon idella*	Grass Carp			X				Guillory and Gasaway 1978
	*Cyprinus carpio*	Common carp						X	Allen 1980
	*Hypophthalmichthys nobilis*	Bighead Carp			X				Kolar et al. 2007
Loricariidae	*Hypostomus plecostomus*	Suckermouth Catfish					X		Hubbs et al. 2008
	*Pterygoplichthys anisitsi*	Southern Sailfin Catfish					X		Nico and Martin 2001
	*Pterygoplichthys disjunctivus*	Vermiculated Sailfin Catfish					X		Nico and Martin 2001
Salmonidae	*Oncorhynchus mykiss*	Rainbow Trout		X					Hubbs et al. 1991
Atherinopsidae	*Membras martinica*	Rough Silverside	X						Hubbs et al. 1991
Fundulidae	*Fundulus grandis*	Gulf Killifish	X						Hubbs et al. 1991
	*Lucania goodei*	Bluefin Killifish		X					Gallaway et al. 2008
Cyprinodontidae	*Cyprinodon variegatus*	Sheepshead Minnow	X						Hubbs et al. 1991
Poeciliidae	*Poecilia formosa*	Amazon Molly	X						Hubbs et al. 1991
	*Poecilia latipinna*	Sailfin Molly	X						Hubbs et al. 1991
	*Poecilia reticulata*	Guppy					X		Hubbs et al. 2008
	*Xiphophorus hellerii*	Green Swordtail		X					Hubbs et al. 2008
	*Xiphophorus variatus*	Variable Platyfish		X					Cohen et al. 2014
Moronidae	*Morone saxatilis*	Striped Bass	X						Hubbs et al. 1991
Centrarchidae	*Ambloplites rupestris*	Rock Bass		X					Hubbs et al. 1991
	*Lepomis auritus*	Redbreast Sunfish		X					Hubbs et al. 1991
	*Micropterus dolomieu*	Smallmouth Bass		X					Hubbs et al. 1991
Percidae	*Sander vitreus*	Walleye		X					Hubbs et al. 1991
Cichlidae	*Oreochromis aureus*	Blue Tilapia				X			Hubbs et al. 2008
	*Oreochromis mossambicus*	Mozambique Tilapia				X			Hubbs et al. 2008
Gobiidae	*Gobiosoma bosc*	Naked Goby	X						T. Bonner, unpublished data

A limitation of the drainage basin checklist and dichotomous keys is that documentation of species by drainage is incomplete. As such, our drainage basin checklists and dichotomous keys should be viewed as living documents and will need periodic updates. While using a drainage basin key, we caution users that the key only includes species known to occur within a basin, and the drainage basin might include more species. If an unknown specimen does not seem to key to a species, we recommend using a key from an adjacent drainage basin. Periodic updates of checklists for Texas inland fishes will come from previously unreported species, non-native species introductions, extirpations of introduced and native fishes, and multiple species described from a single species through genetic analyses. Sources of this information will be dependent on publications and ichthyological records, such as Texas Natural History Collections (Hendrickson and Cohen 2015). In addition to publications and ichthyological records, an emerging tool for documenting species occurrences is the use of citizen science through web-based applications (e.g., iNaturalist, http://www.inaturalist.org). We plan to publish revised checklists and keys following the next release of revised common and scientific names list by the AFS-ASIH Committee of Names of Fishes.

## Supplementary materials

Supplementary material 1 Drainage Basin Keys Data type: Microsoft Word file https://binary.pensoft.net/file/330389This dataset is made available under the Open Database License (http://opendatacommons.org/licenses/odbl/1.0/). The Open Database License (ODbL) is a license agreement intended to allow users to freely share, modify, and use this Dataset while maintaining this same freedom for others, provided that the original source and author(s) are credited.Cody Andrew Craig, Timothy Hallman Bonner
