# Supplementary material for: Drainage basin checklists and dichotomous keys for inland fishes of Texas
Source: Zookeys. 2019 Sep 2;874:31–45. doi: 10.3897/zookeys.874.35618 (PMC6733811; doi:10.3897/zookeys.874.35618)
Supplement: Supplementary material 1 [file zookeys-874-031-s001.docx]

­­­

Drainage Basin Keys

Brazos River 2

Canadian River 12

Colorado and Lavaca Rivers 18

Guadalupe and San Antonio Rivers 29

Nueces River 40

Red River 48

Rio Grande and Pecos River 62

Sabine and Neches Rivers 73

Trinity and San Jacinto Rivers 85

**BRAZOS RIVER BASIN**

KEY TO THE FAMILIES

1a. Both eyes on one side of head; without right pectoral fin American Soles – Achiridae

1b. One eye on either side of head; with both pectoral fins 2

2a. (1b) Body long and slender; without pelvic fins Freshwater Eels – Anguillidae

2b. (1b) Body truncated or elongated; with pelvic fins 3

3a. (2b) Caudal fin heterocercal or abbreviated heterocercal 4

3b. (2b) Caudal fin homocercal 5

4a. (3a) Body covered with ganoid scales; snout formed into a beak; without gular plate Gars – Lepisosteidae

4b. (3a) Body covered with cycloid scales, snout not formed into a beak, with a gular plate Bowfin – Amiidae

5a. (3b) Jaws duckbilled Pickerels – Esocidae

5b. (3b) Jaws not duckbilled 6

6a. (5b) One dorsal fin; pelvic fins without uniserial spines 7

6b. (5b) One or two dorsal fins; pelvic fins with uniserial spines 15

7a. (6a) With adipose fin 8

7b. (6a) Without adipose fin 10

8a. (7a) Without barbels 9

8b. (7a) With barbels Bullhead Catfishes – Ictaluridae

9a. (8a) Scales large, < 50 lateral line scales; incisor teeth present. Tetras – Characidae

9b. (8a) Scales small, > 60 lateral line scales; incisor teeth absent Trouts – Salmonidae

10a. (7b) Long anal fin with ≥ 17 fin rays Shads – Clupeidae

10b. (7b) Short anal fin with ≤ 13 rays 11

11a. (10b) Caudal fin forked or emarginated; lateral line usually present 12

11b. (10b) Caudal fin truncated or rounded; lateral line usually absent 13

12a. (11a) Inferior, fleshy mouth modified for sucking; > 7 pharyngeal teeth in main row, usually ≥ 10 dorsal fin rays Suckers – Catostomidae

12b. (11a) Mouth usually not fleshy or modified for sucking; < 7 pharyngeal teeth in main row, usually ≤ 10 dorsal fin rays Minnows – Cyprinidae

13a. (11b) Mature males with rounded anal fin; males and females with 3rd anal fin ray branched, no gonopodium present 14

13b. (11b) Mature males with pointed anal fin forming a gonopodium; males and females with 3rd anal fin ray unbranched Livebearers – Poeciliidae

14a. (13a) Body robust; teeth in single row are incisor-like and tricuspid (three points on a tooth) Pupfishes – Cyprinodontidae

14b. (13a) Body elongate; conical (cone-shaped) pointed teeth in a single row or several rows Killifishes – Fundulidae

15a. (6b) Anus anterior to pelvic fins; > 5 soft rays on each pelvic fin Pirate Perch – Aphredoderidae

15b. (6b) Anus posterior to pelvic fins; 5 soft rays on pelvic fins 16

16a. (15b) Pelvic fin position abdominal or sub-thoracic; dorsal fins widely separated 17

16b. (15b) Pelvic fin position thoracic; dorsal fins joined or, if separate, closely adjacent to one another 18

17a. (16a) Dorsal fin with 4 thick spines; anal fin with 2-3 spines; adipose eyelids present Mullets – Mugilidae

17b. (16a) Dorsal fin with 4 to 8 thin spines; anal fin with 1 spine; adipose eyelids absent Silversides – Atherinopsidae

18a. (16b) One nostril (nare) on each side of head; lateral line interrupted Cichlids – Cichlidae

18b. (16b) Two nostrils (nares) on each side of head; lateral line complete, incomplete, or absent 19

19a. (18b) Dorsal fin with > 23 fin rays; lateral line extends to tip of caudal fin Drums – Sciaenidae

19b. (18b) Dorsal fin with < 23 fin rays; lateral line, if present, does not extend to tip of caudal fin 20

20a. (19b) Anal fin with 1 to 2 spines Perches – Percidae

20b. (19b) Anal fin with 3 to 8 spines 21

21a. (20b) Posterior margin of operculum with a sharp spine; spiny and soft dorsal fin separate or only slightly connected; pseudobranchium present and exposed Temperate Basses – Moronidae

21b. (20b) Posterior margin of operculum without a sharp spine; spiny and soft dorsal fins connected or with deep notch; pseudobranchium covered or absent 22

22a. (21b) Lateral line present or incomplete Sunfishes – Centrarchidae

22b. (21b) Lateral line absent Pygmy Sunfishes – Elassomatidae

KEY TO THE SPECIES

FAMILY LEPISOSTEIDAE—gars

1a. Large teeth in upper jaw in parallel rows on each side *Atractosteus spatula*

1b. Large teeth in upper jaw in one row, although another non-parallel row might be present 2

2a. (1b) Beak long and narrow, least width goes about 12 to 20 times in length; width of beak at nostrils < eye diameter; snout > ⅔ of head length *Lepisosteus osseus*

2b. (1b) Beak short and blunt, least width goes about 5 to 7 times in length; width of beak at nostrils > eye diameter; snout < ⅔ of head length *Lepisosteus oculatus*

FAMILY AMIIDAE—bowfins

*Amia calva*

FAMILY ANGUILLIDAE—freshwater eels

*Anguilla rostrata*

FAMILY CLUPEIDAE—herrings

1a. Twenty-nine to 33 anal fin rays; mouth subterminal and below level of middle of eye; black

shoulder spot ≥ pupil of eye *Dorosoma cepedianum*

1b. Twenty-four to 28 anal fin rays; mouth terminal and at level of eye; black shoulder spot < pupil of eye *Dorosoma petenense*

FAMILY CYPRINIDAE - minnows

1a. More than 15 soft rays on dorsal fin; dorsal and anal fins each with a strong serrated spine 2

1b. Fewer than 10 soft rays on dorsal fin; dorsal and anal fins without spine 3

2a. (1a) Upper jaw with two pairs of barbels *Cyprinus carpio*

2b. (1a) Upper jaw without barbels *Carassius auratus*

3a. (1b) Anal fin near caudal fin, distance from snout to origin of anal fin is > 2.5 times the distance from origin of anal fin to base of caudal fin; pharyngeal teeth with prominent parallel grooves *Ctenopharyngodon idella*

3b. (1b) Anal fin not noticeably near caudal fin: distance from snout to origin of anal fin is < 2.5 times the distance from origin of anal fin to base of caudal fin; pharyngeal teeth without prominent parallel grooves 4

4a. (3b) Intestine wound spirally around swim bladder; keratinous ridge on lower jaw *Campostoma anomalum*

4b. (3b) Intestine not wound spirally around swim bladder; keratinous ridge of lower jaw hardly evident 5

5a. (4b) Abdomen behind pelvic fins with a fleshy keel lacking scales; lateral line greatly decurved, distance between anterior lateral line scale and ventral most lateral line scale is > 3 scales in height *Notemigonus crysoleucas*

5b. (4b) Abdomen behind pelvic fin with scales; lateral line not greatly decurved, lateral line descends < 3 scales ventrally from highest point 6

6a. (5b) With maxillary barbels, might be small and not observable without opening the mouth or with magnification 7

6b. (5b) Without maxillary barbels 9

7a. (6a) Mouth terminal; distinct black spot located anteriorly on dorsal fin; pharyngeal teeth on main row 5-4 or 5-5 *Semotilus atromaculatus*

7b. (6a) Mouth subterminal or inferior; no distinct black spot on dorsal fin; pharyngeal teeth on main row 4-4 8

8a. (7b) Body silvery, without scattered black specks *Macrhybopsis storeriana*

8b. (7b) Body with scattered black specks *Macrhybopsis hyostoma*

9a. (6b) Predorsal scales appear crowded, smaller than scales on lateral body or appear as

overlapping scales; black spot in the middle, anterior portion of the dorsal fin 10

9b. (6b) Predorsal scales not crowded; without black spot in the middle, anterior portion of the dorsal fin 11

10a. (9a) Caudal spot, if distinct, continuous with mid-lateral stripe; lateral line incomplete; intestine long, more than twice the standard length. *Pimephales promelas*

10b. (9a) Caudal spot distinct from mid-lateral stripe; lateral line complete, intestine forming a short S-shaped loop *Pimephales vigilax*

11a. (9b) Long intestine in a flat coil 12

11b. (9b) Short S-shaped intestine 13

12a. (11a) Head width greater than distance from tip of snout to posterior margin of orbital; internal posterior basiocciptial process is narrow and peg-like, width of internal posterior basiocciptal process fits in to head width at occipital > 7 times *Hybognathus placitus*

12b. (11a) Head width about equal to distance from tip of snout to posterior margin of orbital; internal posterior basiocciptial process is wide and flat, width of internal posterior basioccipital process fits in to head width at occipital < 7 times *Hybognathus nuchalis*

13a. (11b) Moderately decurved lateral line; diamond-shaped scales; dark shoulder patch present; melanophores concentrated between rays of dorsal and anal fins 14

13b. (11b) Lateral line incomplete, complete-straight, or complete-slightly decurved; scales not noticeably diamond-shaped; without dark shoulder patch; melanophores concentrated along rays of dorsal and anal fins 15

14a. (13a) Caudal fin base with a large black spot, about size of eye *Cyprinella venusta*

14b. (13a) Caudal fin base without a large black spot *Cyprinella lutrensis*

15a. (13b) Distinct and separate black dash at base of dorsal fin; apparent when viewed from above; pharyngeal teeth count usually 0,4-4,0 16

15b. (13b) No distinct and separate black dash at base of dorsal fin; pharyngeal teeth count usually 1,4-4,1 or 2,4-4,2 19

16a. (15a) Lateral line scales markedly elevated (taller than wide) anteriorly, elevated scale height 2 to 5 times scale width 17

16b. (15a) Lateral line scales not markedly elevated anteriorly, scale height 1 to 2 times scale width 18

17a. (16a) Dorsal and lateral body with melanophores outlining scales; with pronounced black lateral stripe; dorsal fin height goes 2.1 or more times in pre-dorsal length; infraorbital canal complete *Notropis volucellus*

17b. (16a) Dorsal and lateral body with sparse melanophores; scales outlined with melanophores are rare; pre-dorsal black spot is prominent and distinct from mid-dorsal stripe; dorsal fin height goes 2.0 or fewer times in pre-dorsal length; infraorbital canal incomplete *Notropis buchanani*

18a. (16b) Eye large, eye diameter is > snout length *Notropis stramineus*

18b. (16b) Eye small, eye diameter is < snout length *Notropis buccula*

19a. (15b) Depressed dorsal fin longer than head. *Hybopsis amnis*

19b. (15b) Depressed dorsal fin shorter than head 20

20a. (19b) Mouth is sub-terminal; pharyngeal teeth are 0,4-4,0 *Notropis atrocaudalis*

20b. (19b) Mouth is terminal; pharyngeal teeth are 1,4-4,1 or 2,4-4,2 or 5-5 21

21a. (20b) Dorsal fin origin opposite or anterior to pelvic fin origin 22

21b. (20b) Dorsal fin origin posterior to pelvic fin origin 25

22a. (21a) Prominent mid-lateral stripe; extending through eye 23

22b. (21a) No prominent mid-lateral stripe present 24

23a. (22a) Pharyngeal teeth 5-5; mouth small and almost vertical *Opsopoeodus emiliae*

23b. (22a) Pharyngeal teeth are 1,4-4,1 or 2,4-4,2; mouth large and oblique *Notropis texanus*

24a. (22b) Usually 8 anal fin rays; head is narrow, depth at occiput more than width at occiput *Notropis shumardi*

24b. (22b) Usually 7 anal fin rays; head is wide, depth at occiput less than or equal to width at

occiput *Notropis potteri*

25a. (21b) Small scales, ≥ 41 lateral line scales, > 25 predorsal scales *Lythrurus fumeus*

25b. (21b) Moderate-sized scales, ≤ 40 lateral line scales, < 24 predorsal scales *Notropis oxyrhynchus*

FAMILY CATOSTOMIDAE—suckers

1a. Dorsal fin long, base > than ⅓ of standard length; 22 to 30 dorsal fin rays 2

1b. Dorsal fin short, base < than ¼ of standard length; 4 to 18 dorsal fin rays 5

2a. (1a) Small scales, lateral line scales > 50; eye closer to back of head than to tip of snout; head abruptly more slender than body; papillose lips *Cycleptus elongatus*

2b. (1a) Large scales, lateral line scales < 45; eye closer to tip of snout than back of head; plicate lips 3

3a. (2b) Subopercle triangular, broadest toward base; knob present at tip of lower lip; blunt snout, forming level with eye *Carpiodes carpio*

3b. (2b) Subopercle semicircular, broadest towards middle; knob absent at tip of lower lip; rounded snout, forming below level of eye 4

4a. (3b) Body elongate and slender, greatest body depth goes 2.6 to 3.3 times in standard length, and height of anterior rays in dorsal and anal fins often less than 2/3 head length in individuals >300 mm; small eye, eye diameter goes ≥ 2 times in snout length of individuals <300 mm *Ictiobus niger*

4b. (3b) Body deep and narrow, greatest body depth goes 2.2 to 2.8 times in standard length, and height of anterior dorsal and anal fin rays often greater than 2/3 head length in individuals >300 mm; large eye, eye diameter goes ≤ 2 times in snout length of individuals <300 mm *Ictiobus bubalus*

5a. (1b) Lateral line complete and well developed; air bladder with 3 chambers *Moxostoma congestum*

5b. (1b) Lateral line incomplete or absent; air bladder with 2 chambers 6

6a. (5b) Lateral line incomplete; rows of spots *Minytrema melanops*

6b. (5b) Lateral line absent 7

7a. (6b) Scales larger, lateral scale count 34 to 37; eye larger, eye length ½ of snout length; dorsal fin rays 11 or 12; back with crescentic scale marks *Erimyzon sucetta*

7b. (6b) Scales smaller, lateral scale count 39 to 43; eye smaller, eye length < ½ of snout length; dorsal fin rays 9 or 10; back without crescentic scale marks *Erimyzon claviformis*

FAMILY CHARACIDAE—characins

*Astyanax mexicanus*

FAMILY ICTALURIDAE—bullhead catfishes

1a. Adipose fin joined to the caudal fin or separated by a shallow notch 2

1b. Adipose fin free at tip, not joined to caudal fin 3

2a. (1a) Mouth terminal; pectoral fin spine not serrated; lower lip and chin not heavily speckled with black pigment *Noturus gyrinus*

2b. (1a) Mouth sub-terminal; pectoral spine serrated; lower lip and chin heavily speckled with black pigment. *Noturus nocturnus*

3a. (1b) Head dorso-ventrally compressed; mouth terminal to superior *Pylodictis olivaris*

3b. (1b) Head rounded; mouth subterminal 4

4a. (3b) Caudal fin rounded or shallowly emarginate 5

4b. (3b) Caudal fin deeply forked 6

5a. (4a) Chin barbels completely or partially black; anal fin rays 17 to 24; anal fin broadly rounded *Ameiurus melas*

5b. (4a) Chin barbels white or yellow; anal fin rays 24 to 27; margin of anal fin generally straight *Ameiurus natalis*

6a. (4b) Anal fin rays 30 to 36; anal fin free margin is straight; medial keel-like ridge anterior to dorsal fin forms humped back appearance *Ictalurus furcatus*

6b. (4b) Anal fin rays 22 to 29; anal fin free margin is rounded; no humped back appearance *Ictalurus punctatus*

FAMILY SALMONIDAE—salmons

*Oncorhynchus mykiss*

FAMILY ESOCIDAE—pikes and pickerels

*Esox americanus*

FAMILY APHREDODERIDAE—pirate perch

*Aphredoderus sayanus*

FAMILY MUGILIDAE—mullets

1a. Lower jaw rounded, without a symphyseal knob; lower limb of 1st gill arch with 17 to 20 gill rakers; no adipose eyelid; scales ctenoid *Agonostomus monticola*

1b. Lower jaw angular, with a prominent symphyseal knob; lower limb of 1st gill arch with 25

to 60 gill rakers; adipose eyelid well developed in adults; scales cycloid in young, ctenoid in adults *Mugil cephalus*

FAMILY ATHERINOPSIDAE—New World silversides

1a. Scales small, > 60 scales in lateral series, jaws produced into a short beak; snout length > eye length; > 20 anal fin rays *Labidesthes sicculus*

1b. Scales large, < 50 scales in lateral series; jaws not produced into a beak; snout length ≤ eye length; < 20 anal fin rays *Menidia audens*

FAMILY FUNDULIDAE—topminnows

1a. More than 40 longitudinal scale rows; dark vertical barring; gill slit not extending dorsal to uppermost pectoral fin ray *Fundulus zebrinus*

1b. Fewer than 40 longitudinal scale rows; gill slit extending dorsal to uppermost pectoral fin ray 2

2a. (1b) Body with a distinct black lateral band 3

2b. (1b) Body without a distinct black lateral band 4

3a. (2a) Distinct black spots on anterior dorso-lateral region are as pronounced as lateral stripe; distinct black spots throughout dorsal and caudal fins *Fundulus olivaceus*

3b. (2a) Faint black spots on anterior dorso-lateral region are not as pronounced as lateral stripe; distinct black spots near base of dorsal and caudal fins *Fundulus notatus*

4a. (2b) Dorsal fin originating anterior to anal fin origin; more than 15 scale rows from pelvic fin origin to isthmus predorsal stripe absent or not reaching occiput *Fundulus grandis*

4b. (2b) Dorsal fin originating posterior to anal fin origin 5

5a. (4b) Red to dark spots in multiple rows longitudinally along lateral sides; usually with dark subocular bar *Fundulus blairae*

5b. (4b) Body mottled, barred or irregularly spotted; no dark subocular bar *Fundulus chrysotus*

FAMILY CYPRINODONTIDAE—pupfishes

1a. Abdomen with scales anterior to pelvic fins; distance from origin of dorsal fin to end of hypural plate > distance from origin of dorsal to anterior nostril *Cyprinodon variegatus*

1b. Abdomen without scales anterior to pelvic fins; distance from origin of dorsal fin to end of hypural plate < the distance from origin of dorsal to anterior nostril *Cyprinodon rubrofluviatilis*

FAMILY POECILIIDAE—livebearers

1a. Origin of dorsal fin anterior to anal fin origin; intestinal canal long with many convolutions *Poecilia latipinna*

1b. Origin of dorsal fin posterior to anal fin origin; intestinal canal short with few convolutions

*Gambusia affinis*

FAMILY MORONIDAE—temperate basses

1a. Body depth goes < 3 times in standard length; teeth in single patch on back of

tongue *Morone chrysops*

2b. Body depth goes > 3 times in standard length; teeth in 2 parallel patches on back

of tongue *Morone saxatilis*

FAMILY CENTRARCHIDAE—sunfishes

1a. Five to 8 anal spines 2

1b. Three anal spines 3

2a. (1a) Dorsal fin set back on body, length of dorsal fin base < distance from its origin to posterior margin of eye; lateral body with wide to narrow dorsal black bands; ≤ 6 dorsal spines *Pomoxis annularis*

2b. (1a) Dorsal fin set forward on body, length of dorsal fin base equal to or greater than distance from its origin to posterior margin of eye; lateral body with checkerboard black and light pattern; ≥7 dorsal spines *Pomoxis nigromaculatus*

3a. (1b) Body slender, body depth contained > 3 times into standard length 4

3b. (1b) Body deep, body depth contained < 3 times into standard length 7

4a. (3a) Dorsal fins narrowly joined at base, forming a deep notch; upper jaw extends past posterior margin of eye in adults; mid-lateral stripe generally complete, rows of spots ventral to mid-lateral stripe faint and incomplete *Micropterus salmoides*

4b. (3a) Dorsal fins broadly joined at base forming a shallow notch; upper jaw does not reach past posterior portion of eye; bases of soft dorsal and anal fins scaled 5

5a. (4b) No tooth patch on tongue; lower lateral region scales without black spots forming horizontal rows *Micropterus dolomieu*

5b. (4b) Tooth patch on tongue; lower lateral region scales with black spots forming horizontal rows 6

6a. (5b) Mid-lateral stripe often appears interrupted anteriorly, rows of spots ventral to mid-lateral stripe distinct and complete. *Micropterus punctulatus*

6b. (5b) Dark wide midlateral stripe present and disconnected anteriorly into a narrow midlateral stripe posteriorly, forming vertical bars *Micropterus treculii*

7a. (3b) Teeth on tongue; head and opercle with 3 to 5 distinct dark and light longitudinal stripes; red spot on posterior margin of opercle flap in fresh specimens *Lepomis gulosus*

7b. (3b) No teeth on tongue; head and opercle lacking distinct dark and light longitudinal stripe 8

8a. (7b) Pectoral fins long and pointed, reach anterior portion of eye or beyond when bent forward 9

8b. (7b) Pectoral fins short and rounded, do not reach past eye when bent forward 11

9a. (8a) Opercle flap stiff to its margin, posterior margin either red or orange in live specimens *Lepomis microlophus*

9b. (8a) Opercle flap flexible, posterior margin not red or orange in live specimens 10

10a. (9b) Opercle flap black to the margin; black spot on posterior base of soft dorsal fin *Lepomis macrochirus*

10b. (9b) Opercle flap outlined with thick white band; lacking black spot on posterior base of soft dorsal fin *Lepomis humilis*

11a. (8b) Black opercle flap stiff near the posterior margin with bone supporting all or majority of the flap 12

11b. (8b) Black opercle flap flexible near the posterior margin without bone supporting majority of the flap 14

12a. (11a) Lateral line incomplete; smaller individuals with black spot surrounded by white margin on posterior base of soft dorsal fin *Lepomis symmetricus*

12b. (11a) Lateral line complete; black spot, if present, on posterior base of soft dorsal fin without white margin 13

13a. (12b) Body elongated with black spot on posterior base of soft dorsal fin *Lepomis cyanellus*

13b. (12b) Body rounded without black spot on posterior base of soft dorsal fin; lateral body with alternating stripes formed from black and red spots *Lepomis miniatus*

14a. (11b) Opercle flap black to the posterior margin; opercle flap is thin near the opercle bone with the narrowest width of the flexible portion of the flap about the same diameter of the eye pupil *Lepomis auritus*

14b. (11b) Opercle flap black and surrounded by white on the posterior margin; opercle flap is wide with narrowest width of the flexible flap is about two times the diameter of the eye pupil 15

15a. (14b) Twelve pectoral fin rays, 3 to 5 cheek scales; opercle flap often with white pigment form speckles, distinct red spots (white in preserved specimens) along lateral line *Lepomis marginatus*

15b. (14b) Thirteen to 15 pectoral fin rays, 5 to 7 cheek scales; opercle flaps with red or white margin; 13 to 15 pectoral fin rays *Lepomis megalotis*

FAMILY PERCIDAE—perches

1a. Snout conical, extends beyond upper lip; body with ≥ 14 black vertical bars 2

1b. Snout less conical, does not extend beyond upper lip; body with < 14 black vertical bars or with a pattern other than vertical bars 3

2a. (1a) Body with thick vertical bars, bars alternate in length from long to short; 9 to 10 long bars *Percina carbonaria*

2b. (1a) Body with 14 to 16 thin vertical bars of similar length *Percina macrolepida*

3a. (1b) Sides of body with large black blotches; midline of abdomen naked or with enlarged scales *Percina sciera*

3b. (1b) Sides of body without large black blotches; scales on abdomen normal 4

4a. (3b) Lateral line arched upward *Etheostoma gracile*

4b. (3b) Lateral line straight 5

5a. (4b) Lateral body with distinct series of M-shaped pigments; snout rounded and blunt *Etheostoma chlorosoma*

5b. (4b) Lateral body without distinct series of M-shaped pigments; snout not noticeably rounded and blunt 6

6a. (5b) Lateral region with mottling bisected by a light colored lateral stripe *Etheostoma parvipinne*

6b. (5b) Anterior portion of lateral region with black horizontal dashes and posterior portion of lateral region with 8 to 11 vertical bars; throat of live males orange *Etheostoma spectabile*

FAMILY SCIAENIDAE—drums

*Aplodinotus grunniens*

FAMILY ELASSOMATIDAE—pygmy sunfishes

*Elassoma zonatum*

FAMILY CICHLIDAE—cichlids

1a. Anal fin spines 5 to 6 *Herichthys cyanoguttatus*

1b. Anal fin spines < 5 (usually 3) *Oreochromis aureus*

FAMILY ACHIRIDAE—American soles

*Trinectes maculatus*

**CANADIAN RIVER BASIN**

KEY TO THE FAMILIES

1a. With adipose fin 2

1b. Without adipose fin 3

2a. (1a) Without barbels Trouts – Salmonidae

2b. (1a) With barbels Bullhead Catfishes – Ictaluridae

3a. (1b) One dorsal fin; pelvic fins without uniserial spines 4

3b. (1b) One or two dorsal fins; pelvic fins with uniserial spines 9

4a. (3a) Long anal fin with ≥ 17 fin rays Shads – Clupiedae

4b. (3a) Short anal fin with ≤ 13 rays 5

5a. (4b) Caudal fin forked or emarginated; lateral line usually present 6

5b. (4b) Caudal fin truncated or rounded; lateral line usually absent 7

6a. (5a) Inferior, fleshy mouth modified for sucking; > 7 pharyngeal teeth in main row, usually ≥ 10 dorsal fin rays Suckers – Catostomidae

6b. (5a) Mouth usually not fleshy or modified for sucking; < 7 pharyngeal teeth in main row, usually ≤ 10 dorsal fin rays Carps and Minnows – Cyprinidae

7a. (5b) Mature males with rounded anal fin; males and females with 3rd anal fin ray branched, no gonopodium present 8

7b. (5b) Mature males with pointed anal fin forming a gonopodium; males and females with 3rd anal fin ray unbranched Livebearers – Poeciliidae

8a. (7a) Body robust; teeth in single row are incisor-like and tricuspid (three points on a tooth). Pupfishes – Cyprinodontidae

8b. (7a) Body elongate; conical (cone-shaped) pointed teeth in a single row or several rows Killifishes – Fundulidae

9a. (3b) Pelvic fin position abdominal or sub-thoracic; dorsal fins widely separated Silversides – Atherinopsinidae

9b. (3b) Pelvic fin position thoracic; dorsal fins joined or, if separate, closely adjacent to one another 10

10a. (9b) Anal fin with 1 to 2 spines Perches – Percidae

10b. (9b) Anal fin with 3 to 8 spines 11

11a. (10b) Posterior margin of operculum with a sharp spine; spiny and soft dorsal fin separate or only slightly connected; pseudobranchium present and exposed Temparate Basses – Moronidae

11b. (10b) Posterior margin of operculum without a sharp spine; spiny and soft dorsal fins connected or with deep notch; pseudobranchium covered or absent Sunfishes – Centrarchidae

KEY TO THE SPECIES

FAMILY CLUPEIDAE—herrings

1a. Twenty-nine to 33 anal fin rays; mouth subterminal and below level of middle of eye; black

shoulder spot ≥ pupil of eye *Dorosoma cepedianum*

1b. Twenty-four to 28 anal fin rays; mouth terminal and at level of eye; black shoulder spot < pupil of eye *Dorosoma petenense*

FAMILY CYPRINIDAE - minnows

1a. More than 15 soft rays on dorsal fin; dorsal and anal fins each with a strong serrated spine *Cyprinus carpio*

1b. Fewer than 10 soft rays on dorsal fin; dorsal and anal fins without spine 2

2a. (1b) Anal fin near caudal fin, distance from snout to origin of anal fin is > 2.5 times the distance from origin of anal fin to base of caudal fin; pharyngeal teeth with prominent parallel grooves *Ctenopharyngodon idella*

2b. (1b) Anal fin not noticeably near caudal fin: distance from snout to origin of anal fin is < 2.5 times the distance from origin of anal fin to base of caudal fin; pharyngeal teeth without prominent parallel grooves 3

3a. (2b) Intestine wound spirally around swim bladder; keratinous ridge on lower jaw *Campostoma anomalum*

3b. (2b) Intestine not wound spirally around swim bladder; keratinous ridge of lower jaw hardly evident 4

4a. (3b) Abdomen behind pelvic fins with a fleshy keel lacking scales; lateral line greatly decurved, distance between anterior lateral line scale and ventral most lateral line scale is > 3 scales in height *Notemigonus crysoleucas*

4b. (3b) Abdomen behind pelvic fin with scales; lateral line not greatly decurved, lateral line descends < 3 scales ventrally from highest point 5

5a. (4b) With maxillary barbels, might be small and not observable without opening the mouth or with magnification 6

5b. (4b) Without maxillary barbels 7

6a. (5a) Body with scattered black specks *Macrhybopsis tetranema*

6b. (5a) Body silvery, without scattered black specks *Platygobio gracilis*

7a. (5b) Thick lower lip at corners, mouth noticeably ventral; black spot at base of caudal fin *Phenacobius mirabilis*

7b. (5b) Lower lip thin or not noticeably thick; with or without black spot at base of caudal fin 8

8a. (7b) Predorsal scales appear crowded, smaller than scales on lateral body or appear as

overlapping scales; black spot in the middle, anterior portion of the dorsal fin 9

8b. (7b) Predorsal scales not crowded; without black spot in the middle, anterior portion of the dorsal fin 10

9a. (8a) Caudal spot, if distinct, continuous with mid-lateral stripe; lateral line incomplete; intestine long, more than twice the standard length. *Pimephales promelas*

9b. (8a) Caudal spot distinct from mid-lateral stripe; lateral line complete, intestine forming a short S-shaped loop *Pimephales vigilax*

10a. (8b) Long intestine in a flat coil *Hybognathus placitus*

10b. (8b) Short S-shaped intestine 11

11a. (10b) Moderately decurved lateral line; diamond-shaped scales; dark shoulder patch present; melanophores concentrated between rays of dorsal and anal fins *Cyprinella lutrensis*

11b. (10b) Lateral line incomplete, complete-straight, or complete-slightly decurved; scales not noticeably diamond-shaped; without dark shoulder patch; melanophores concentrated along rays of dorsal and anal fins 12

12a. (11b) No distinct and separate black dash at base of dorsal fin; pharyngeal teeth count usually 1,4-4,1 or 2,4-4,2 13

12b. (11b) Distinct and separate black dash at base of dorsal fin; apparent when viewed from above; pharyngeal teeth count usually 0,4-4,0 14

13a. (12a) Dorsal fin origin opposite to pelvic fin origin *Notropis blennius*

13b. (12a) Dorsal fin origin posterior to pelvic fin origin *Notropis atherinoides*

14a. (12b) Eye large, eye diameter is > snout length *Notropis stramineus*

14b. (12b) Eye small, eye diameter is < snout length *Notropis girardi*

FAMILY CATOSTOMIDAE—suckers

*Carpiodes carpio*

FAMILY ICTALURIDAE—bullhead catfishes

1a. Head dorso-ventrally compressed; mouth terminal to superior *Pylodictis olivaris*

1b. Head rounded; mouth subterminal 2

2a. (1b) Caudal fin rounded or shallowly emarginate 3

2b. (1b) Caudal fin deeply forked *Ictalurus punctatus*

3a. (2a) Chin barbels completely or partially black; anal fin rays 17 to 24; anal fin broadly rounded *Ameiurus melas*

3b. (2a) Chin barbels white or yellow; anal fin rays 24 to 27; margin of anal fin generally straight *Ameiurus natalis*

FAMILY SALMONIDAE—salmons

*Oncorhynchus mykiss*

FAMILY ATHERINOPSIDAE—New World silversides

*Menidia audens*

FAMILY FUNDULIDAE—topminnows

*Fundulus kansae*

FAMILY POECILIIDAE—livebearers

*Gambusia affinis*

FAMILY CYPRINODONTIDAE— pupfishes

*Cyprinodon rubrofluviatilis*

FAMILY MORONIDAE—temperate basses

*Morone chrysops*

FAMILY CENTRARCHIDAE—sunfishes

1a. Five to 8 anal spines *Pomoxis annularis*

1b. Three anal spines 2

2a. (1b) Body slender, body depth contained > 3 times into standard length 3

2b. (1b) Body deep, body depth contained < 3 times into standard length 4

3a. (2a) Dorsal fins narrowly joined at base forming a deep notch; upper jaw extends past posterior margin of eye in adults; mid-lateral stripe generally complete, rows of spots ventral to mid-lateral stripe faint and incomplete *Micropterus salmoides*

3b. (2a) Dorsal fins broadly joined at base forming a shallow notch; upper jaw does not reach past posterior portion of eye; bases of soft dorsal and anal fins scaled *Micropterus dolomieu*

4a. (2b) Pectoral fins long and pointed, reach anterior portion of eye or beyond when bent forward 5

4b. (2b) Pectoral fins short and rounded, do not reach past eye when bent forward 7

5a. (4a) Opercle flap stiff to its margin, posterior margin either red or orange in live specimens *Lepomis microlophus*

5b. (4a) Opercle flap flexible, posterior margin not red or orange in live specimens 6

6a. (5b) Opercle flap black to the margin; black spot on posterior base of soft dorsal fin *Lepomis macrochirus*

6b. (5b) Opercle flap outlined with thick white band; lacking black spot on posterior base of soft dorsal fin *Lepomis humilis*

7a. (4b) Black opercle flap stiff near the posterior margin with bone supporting all or majority of the flap *Lepomis cyanellus*

7b. (4b) Black opercle flap flexible near the posterior margin without bone supporting majority of the flap *Lepomis megalotis*

FAMILY PERCIDAE—perches

*Sander vitreus*

**COLORADO AND LAVACA RIVER BASINS**

KEY TO THE FAMILIES

1a. Both eyes on one side of head; without right pectoral fin American Soles – Achiridae

1b. One eye on either side of head; with both pectoral fins 2

2a. (1b) Caudal fin heterocercal or abbreviated heterocercal 3

2b. (1b) Caudal fin homocercal 4

3a. (2a) Body with ganoid scales; snout formed into a beak; without gular plate Gars – Lepiososteidae

3b. (2a) Body covered with cycloid scales, snout not formed into a beak, with a gular plate Bowfin – Amiidae

4a. (2b) One dorsal fin; pelvic fins without uniserial spines 5

4b. (2b) One or two dorsal fins; pelvic fins with uniserial spines 13

5a. (4a) With adipose fin 6

5b. (4a) Without adipose fin 8

6a. (5a) Without barbels 7

6b. (5a) With barbels Bullhead Catfishes – Ictaluridae

7a. (6a) Scales large, < 50 lateral line scales; incisor teeth present Tetras – Characidae

7b. (6a) Scales small, > 60 lateral line scales; incisor teeth absent Trouts – Salmonidae

8a. (5b) Long anal fin with ≥ 17 fin rays Shads – Clupiedae

8b. (5b) Short anal fin with ≤ 13 rays 9

9a. (8b) Caudal fin forked or emarginated; lateral line usually present 10

9b. (8b) Caudal fin truncated or rounded; lateral line usually absent 11

10a. (9a) Inferior, fleshy mouth modified for sucking; > 7 pharyngeal teeth in main row, usually ≥ 10 dorsal fin rays Suckers – Catostomidae

10b. (9a) Mouth usually not fleshy or modified for sucking; < 7 pharyngeal teeth in main row, usually ≤ 10 dorsal fin rays Carps and Minnows – Cyprinidae

11a. (9b) Mature males with rounded anal fin; males and females with 3rd anal fin ray branched, no gonopodium present 12

11b. (9b) Mature males with pointed anal fin forming a gonopodium; males and females with 3rd anal fin ray unbranched Livebearers – Poeciliidae

12a. (11a) Body robust; teeth in single row are incisor-like and tricuspid (three points on a tooth). Pupfishes – Cyprinodontidae

12b. (11a) Body elongate; conical (cone-shaped) pointed teeth in a single row or several rows Killifishes – Fundulidae

13a. (4b) Anus anterior to pelvic fins; > 5 soft rays on each pelvic fin Pirate perch – Aphredoderidae

13b. (4b) Anus posterior to pelvic fins; 5 soft rays on pelvic fins 14

14a. (13b) Pelvic fin position abdominal or sub-thoracic; dorsal fins widely separated 15

14b. (13b) Pelvic fin position thoracic; dorsal fins joined or, if separate, closely adjacent to one another 16

15a. (14a) Dorsal fin with 4 thick spines; anal fin with 2-3 spines; adipose eyelids present Mullets – Mugilidae

15b. (14a) Dorsal fin with 4 to 8 thin spines; anal fin with 1 spine; adipose eyelids absent Silversides – Atherinopsinidae

16a. (14b) One nostril (nare) on each side of head; lateral line interrupted Cichlids – Cichlidae

16b. (14b) Two nostrils (nares) on each side of head; lateral line complete, incomplete, or absent 17

17a. (16b) Dorsal fin with > 23 fin rays; lateral line extends to tip of caudal fin Drums – Sciaenidae

17b. (16b) Dorsal fin with < 23 fin rays; lateral line, if present, does not extend to tip of caudal fin 18

18a. (17b) Pelvic fins joined into a sucking disk, gill membranes broadly joined to isthmus Gobies – Gobiidae

18b. (17b) Pelvic fins not joined, gill membranes free or nearly free from isthmus (may be joined to each other across isthmus) 19

19a. (18b) Anal fin with 1 to 2 spines Perches – Percidae

19b. (18b) Anal fin with 3 to 8 spines 20

20a. (19b) Posterior margin of operculum with a sharp spine; spiny and soft dorsal fin separate or only slightly connected; pseudobranchium present and exposed Temperate Basses – Moronidae

20b. (19b) Posterior margin of operculum without a sharp spine; spiny and soft dorsal fins connected or with deep notch; pseudobranchium covered or absent Sunfishes – Centrarchidae

KEY TO THE SPECIES

FAMILY LEPISOSTEIDAE—gars

1a. Large teeth in upper jaw in parallel rows on each side *Atractosteus spatula*

1b. Large teeth in upper jaw one row, although another non-parallel row might be present 2

2a. (1b) Beak long and narrow, least width goes about 12 to 20 times in length; width of beak at nostrils < eye diameter; snout > ⅔ of head length *Lepisosteus osseus*

2b. (1b) Beak short and blunt, least width goes about 5 to 7 times in length; width of beak at nostrils > eye diameter; snout < ⅔ of head length *Lepisosteus oculatus*

FAMILY AMIIDAE—bowfins

*Amia calva*

FAMILY ANGUILLIDAE—freshwater eels

*Anguilla rostrata*

FAMILY CLUPEIDAE—herrings

1a. Twenty-nine to 33 anal fin rays; mouth subterminal and below level of middle of eye; black

shoulder spot ≥ pupil of eye *Dorosoma cepedianum*

1b. Twenty-four to 28 anal fin rays; mouth terminal and at level of eye; black shoulder spot < pupil of eye *Dorosoma petenense*

FAMILY CYPRINIDAE - minnows

1a. More than 15 soft rays on dorsal fin; dorsal and anal fins each with a strong serrated spine 2

1b. Fewer than 10 soft rays on dorsal fin; dorsal and anal fins without spine 3

2a. (1a) Upper jaw with two pairs of barbels *Cyprinus carpio*

2b. (1a) Upper jaw without barbels *Carassius auratus*

3a. (2b) Anal fin near caudal fin, distance from snout to origin of anal fin is > 2.5 times the distance from origin of anal fin to base of caudal fin; pharyngeal teeth with prominent parallel grooves *Ctenopharyngodon idella*

3b. (2b) Anal fin not noticeably near caudal fin: distance from snout to origin of anal fin is < 2.5 times the distance from origin of anal fin to base of caudal fin; pharyngeal teeth without prominent parallel grooves 4

4a. (3b) Intestine wound spirally around swim bladder; keratinous ridge on lower jaw *Campostoma anomalum*

4b. (3b) Intestine not wound spirally around swim bladder; keratinous ridge of lower jaw hardly evident 5

5a. (4b) Abdomen behind pelvic fins with a fleshy keel lacking scales; lateral line greatly decurved, distance between anterior lateral line scale and ventral most lateral line scale is > 3 scales in height *Notemigonus crysoleucas*

5b. (4b) Abdomen behind pelvic fin with scales; lateral line not greatly decurved, lateral line descends < 3 scales ventrally from highest point 6

6a. (5b) With maxillary barbels, might be small and not observable without opening the mouth or with magnification 7

6b. (5b) Without maxillary barbels 8

7a. (6a) Prominent mid-lateral stripe reaching from opercle to caudal peduncle *Macrhybopsis marconis*

7b. (6a) Mid-lateral stripe incomplete or absent, more pronounced posteriorly on caudal peduncle when incomplete *Macrhybopsis hyostoma*

8a. (6b) Thick lower lip at corners, mouth noticeably ventral; black spot at base of caudal fin *Phenacobius mirabilis*

8b. (6b) Lower lip thin or not noticeably thick; with or without black spot at base of caudal fin 9

9a. (8b) Predorsal scales appear crowded, smaller than scales on lateral body or appear as

overlapping scales; black spot in the middle, anterior portion of the dorsal fin 10

9b. (8b) Predorsal scales not crowded; without black spot in the middle, anterior portion of the dorsal fin 11

10a. (9a) Caudal spot, if distinct, continuous with mid-lateral stripe; lateral line incomplete; intestine long, more than twice the standard length. *Pimephales promelas*

10b. (9a) Caudal spot distinct from mid-lateral stripe; lateral line complete, intestine forming a short S-shaped loop *Pimephales vigilax*

11a. (9b) Long intestine in a flat coil 12

11b. (9b) Short S-shaped intestine 13

12a. (11a) Black mid-lateral stripe extends through eye to snout; eye width greater than or equal to snout length *Dionda nigrotaeniata*

12b. (11a) Mid-lateral stripe, if present (sometimes appears as a broad, diffuse band of melanophores), does not extend through the eye to the snout; eye width less than snout length *Hybognathus placitus*

13a. (11b) Moderately decurved lateral line; diamond-shaped scales; dark shoulder patch present; melanophores concentrated between rays of dorsal and anal fins 14

13b. (11b) Lateral line incomplete, complete-straight, or complete-slightly decurved; scales not noticeably diamond-shaped; without dark shoulder patch; melanophores concentrated along rays of dorsal and anal fins 15

14a. (13a) Sub-terminal mouth; caudal fin base with a spot larger than eye *Cyprinella venusta*

14b. (13a) Terminal mouth; no caudal spot *Cyprinella lutrensis*

15a. (13b) Distinct and separate black dash at base of dorsal fin; apparent when viewed from above; pharyngeal teeth count usually 0,4-4,0 16

15b. (13b) No distinct and separate black dash at base of dorsal fin; pharyngeal teeth count usually 1,4-4,1 or 2,4-4,2 19

16a. (15a) Lateral line scales markedly elevated (taller than wide) anteriorly, elevated scale height 2 to 5 times scale width 17

16b. (15a) Lateral line scales not markedly elevated anteriorly, scale height 1 to 2 times scale width 18

17a. (16a) Dorsal and lateral body with melanophores outlining scales; with pronounced black lateral stripe; dorsal fin height goes 2.1 or more times in pre-dorsal length; infraorbital canal complete *Notropis volucellus*

17b. (16a) Dorsal and lateral body with sparse melanophores; scales outlined with melanophores are rare; pre-dorsal black spot is prominent and distinct from mid-dorsal stripe; dorsal fin height goes 2.0 or fewer times in pre-dorsal length; infraorbital canal incomplete *Notropis buchanani*

18a. (16b) Eye large, eye diameter is > snout length *Notropis stramineus*

18b. (16b) Eye small, eye diameter is < snout length *Notropis buccula*

19a. (15b) Depressed dorsal fin longer than head. *Hybopsis amnis*

19b. (15b) Depressed dorsal fin shorter than head 20

20a. (19b) Dorsal fin origin opposite or anterior to pelvic fin origin 21

20b. (19b) Dorsal fin origin posterior to pelvic fin origin 24

21a. (20a) Prominent mid-lateral stripe; extending through eye 22

21b. (20a) No prominent mid-lateral stripe present *Notropis shumardi*

22a. (21a) Pharyngeal teeth 5-5; mouth small and almost vertical *Opsopoeodus emiliae*

22b. (21a) Pharyngeal teeth are 1,4-4,1 or 2,4-4,2; mouth large and oblique *Notropis texanus*

23a. (20b) Small scales, ≥ 41 lateral line scales, > 25 predorsal scales *Lythrurus fumeus*

23b. (20b) Moderate-sized scales, ≤ 40 lateral line scales, < 24 predorsal scales 24

24a. (23b) Eye larger, eye diameter > snout length *Notropis amabilis*

24b. (23b) Eye smaller, eye diameter < snout length *Notropis oxyrhynchus*

FAMILY CATOSTOMIDAE—suckers

1a. Dorsal fin long, base > than ⅓ of standard length; 22 to 30 dorsal fin rays 2

1b. Dorsal fin short, base < than ¼ of standard length; 4 to 18 dorsal fin rays 5

2a. (1a) Small scales, lateral line scales > 50; eye closer to back of head than to tip of snout;

head abruptly more slender than body; papillose lips *Cycleptus elongatus*

2b. (1a) Large scales, lateral line scales < 45; eye closer to tip of snout than back of head; plicate lips 3

3a. (2b) Subopercle triangular, broadest toward base; knob present at tip of lower lip; blunt snout, forming level with eye *Carpiodes carpio*

3b. (2b) Subopercle semicircular, broadest towards middle; knob absent at tip of lower lip; rounded snout, forming below level of eye 4

4a. (3b) Body elongate and slender, greatest body depth goes 2.6 to 3.3 times in standard length, and height of anterior rays in dorsal and anal fins often less than 2/3 head length in individuals >300 mm; small eye, eye diameter goes ≥ 2 times in snout length of individuals <300 mm *Ictiobus niger*

4b. (3b) Body deep and narrow, greatest body depth goes 2.2 to 2.8 times in standard length, and height of anterior dorsal and anal fin rays often greater than 2/3 head length in individuals >300 mm; large eye, eye diameter goes ≤ 2 times in snout length of individuals <300 mm *Ictiobus bubalus*

5a. (1b) Lateral line complete and well developed; air bladder with 3 chambers; no rows of spots present *Moxostoma congestum*

5b. (1b) Lateral line incomplete or absent; air bladder with 2 chambers; rows of spots present *Minytrema melanops*

FAMILY CHARACIDAE—characins

*Astyanax mexicanus*

FAMILY ICTALURIDAE—bullhead catfishes

1a. Adipose fin joined to the caudal fin or separated by a shallow notch *Noturus gyrinus*

1b. Adipose fin free at tip, not joined to caudal fin 2

2a. (1b) Head dorso-ventrally compressed; mouth terminal to superior *Pylodictis olivaris*

2b. (1b) Head rounded; mouth subterminal 3

3a. (2b) Caudal fin rounded or shallowly emarginate 4

3b. (2b) Caudal fin deeply forked 5

4a. (3a) Chin barbels completely or partially black; anal fin rays 17 to 24; anal fin broadly rounded *Ameiurus melas*

4b. (3a) Chin barbels white or yellow; anal fin rays 24 to 27; margin of anal fin generally straight *Ameiurus natalis*

5a. (3b) Anal fin rays 30 to 36; anal fin free margin is straight; medial keel-like ridge anterior to dorsal fin forms humped back appearance *Ictalurus furcatus*

5b. (3b) Anal fin rays 22 to 29; anal fin free margin is rounded; no humped back appearance 6

6a. (5b) Anal fin rays 27 to 29; pectoral fin spine goes < 5 times into standard length; random scattering of few black spots may be present *Ictalurus punctatus*

6b. (5b) Anal fin rays 22 to 26; pectoral fin spine goes > 5 times into standard length; diffuse black spots on sides *Ictalurus lupus*

FAMILY SALMONIDAE—salmons

*Oncorhynchus mykiss*

FAMILY APHREDODERIDAE—pirate perch

*Aphredoderus sayanus*

FAMILY MUGILIDAE—mullets

1a. Lower jaw rounded, without a symphyseal knob; lower limb of 1st gill arch with 17 to 20 gill rakers; no adipose eyelid; scales ctenoid *Agonostomus monticola*

1b. Lower jaw angular, with a prominent symphyseal knob; lower limb of 1st gill arch with 25

to 60 gill rakers; adipose eyelid well developed in adults; scales cycloid in young, ctenoid in adults. *Mugil cephalus*

FAMILY ATHERINOPSIDAE—New World silversides

*Menidia audens*

FAMILY FUNDULIDAE—topminnows

1a. Distance from origin of dorsal fin to end of hypural plate < distance from origin of dorsal

fin to preopercle or occasionally about equal to that distance; more than 30 longitudinal scale

rows 2

1b. Distance from origin of dorsal fin to end of hypural plate > distance from origin of dorsal fin to preopercle; 30 or fewer longitudinal scale rows *Lucania parva*

2a. (1a) More than 40 longitudinal scale rows; dark vertical barring; gill slit not extending dorsal to uppermost pectoral fin ray *Fundulus zebrinus*

2b. (1a) Fewer than 40 longitudinal scale rows; gill slit extending dorsal to uppermost pectoral fin ray 3

3a. (2b) Body with a distinct black lateral band *Fundulus notatus*

3b. (2b) Body without a distinct black lateral band 4

4a. (3b) Dorsal fin originating posterior to anal fin origin *Fundulus chrysotus*

4b. (3b) Dorsal fin originating anterior to anal fin origin; > 15 scale rows from pelvic fin origin to isthmus; predorsal stripe absent or not reaching occiput *Fundulus grandis*

FAMILY CYPRINODONTIDAE— pupfishes

1a. Abdomen with scales anterior to pelvic fins; distance from origin of dorsal fin to end of hypural plate > distance from origin of dorsal to anterior nostril *Cyprinodon variegatus*

1b. Abdomen without scales anterior to pelvic fins; distance from origin of dorsal fin to end of hypural plate < the distance from origin of dorsal to anterior nostril *Cyprinodon rubrofluviatilis*

FAMILY POECILIIDAE—livebearers

1a. Origin of dorsal fin anterior to anal fin origin; intestinal canal long with many convolutions 2

1b. Origin of dorsal fin posterior to anal fin origin; intestinal canal short with few convolutions 3

2a. (1a) Teeth in single row; dorsal fin rays ≥ 12; parallel rows of spots usually present *Poecilia latipinna*

2b. (1a) Teeth in villiform bands; dorsal fin rays ≤ 12; spots, if present, are diffuse *Xiphophorus variatus*

3a. (1b) Spines at tip of 3rd anal fin ray of male gonopodium (first enlarged ray) 1 to 3 times longer than wide *Gambusia affinis*

3b. (1b) Spines at tip of 3rd anal fin ray of male gonopodium 4 to 10 times longer than wide 4

4a. (3b) Dorsal and anal fins without yellow pigmentation; dusky lateral stripe indistinct; mouth without black markings and anal spot of females not restricted to area immediately around anus; pectoral fin of males with indentation, much deeper than widest pectoral fin ray; found in Clear Creek, Menard County only *Gambusia heterochir*

4b. (3b) Dorsal and (in females) anal fins with yellow pigmentation (lost in preservation); males with a shallow pectoral indentation *Gambusia geiseri*

FAMILY MORONIDAE—temperate basses

1a. Body depth goes < 3 times in standard length; teeth in single patch on back of tongue *Morone chrysops*

1b. Body depth goes > 3 times in standard length; teeth in 2 parallel patches on back of tongue *Morone saxatilis*

FAMILY CENTRARCHIDAE—sunfishes

1a. Five to 8 anal spines 2

1b. Three anal spines 3

2a. (1a) Dorsal fin set back on body, length of dorsal fin base < distance from its origin to posterior margin of eye; lateral body with wide to narrow dorsal black bands; ≤ 6 dorsal spines *Pomoxis annularis*

2b. (1a) Dorsal fin set forward on body, length of dorsal fin base equal to or greater than distance from its origin to posterior margin of eye; lateral body with checkerboard black and light pattern; ≥7 dorsal spines *Pomoxis nigromaculatus*

3a. (1b) Body slender, body depth contained > 3 times into standard length 4

3b. (1b) Body deep, body depth contained < 3 times into standard length 7

4a. (3a) Dorsal fins narrowly joined at base forming a deep notch; upper jaw extends past posterior margin of eye in adults; mid-lateral stripe generally complete, rows of spots ventral to mid-lateral stripe faint and incomplete *Micropterus salmoides*

4b. (3a) Dorsal fins broadly joined at base forming a shallow notch; upper jaw does not reach past posterior portion of eye; bases of soft dorsal and anal fins scaled 5

5a. (4b) No tooth patch on tongue; lower lateral region scales without black spots forming horizontal rows *Micropterus dolomieu*

5b. (4b) Tooth patch on tongue; lower lateral region scales with black spots forming horizontal rows 6

6a. (5b) Mid-lateral stripe often appears interrupted anteriorly, rows of spots ventral to mid-lateral stripe distinct and complete. *Micropterus punctulatus*

6b. (5b) Dark wide midlateral stripe present and disconnected anteriorly into a narrow midlateral stripe posteriorly, forming vertical bars *Micropterus treculii*

7a. (3b) Teeth on tongue; head and opercle with 3 to 5 distinct dark and light longitudinal stripes; red spot on posterior margin of opercle flap in fresh specimens *Lepomis gulosus*

7b. (3b) No teeth on tongue; head and opercle lacking distinct dark and light longitudinal stripes 8

8a. (7b) Pectoral fins long and pointed, reach anterior portion of eye or beyond when bent forward 9

8b. (7b) Pectoral fins short and rounded, do not reach past eye when bent forward 11

9a. (8a) Opercle flap stiff to its margin, posterior margin either red or orange in live specimens *Lepomis microlophus*

9b. (8a) Opercle flap flexible, posterior margin not red or orange in live specimens 10

10a. (9b) Opercle flap black to the margin; black spot on posterior base of soft dorsal fin *Lepomis macrochirus*

10b. (9b) Opercle flap outlined with thick white band; lacking black spot on posterior base of soft dorsal fin *Lepomis humilis*

11a. (8b) Black opercle flap stiff near the posterior margin with bone supporting all or majority of the flap 12

11b. (8b) Black opercle flap flexible near the posterior margin without bone supporting majority of the flap 14

12a. (11a) Lateral line incomplete; smaller individuals with black spot surrounded by white margin on posterior base of soft dorsal fin *Lepomis symmetricus*

12b. (11a) Lateral line complete; black spot, if present, on posterior base of soft dorsal fin without white margin 13

13a. (12b) Body elongated with black spot on posterior base of soft dorsal fin *Lepomis cyanellus*

13b. (12b) Body rounded without black spot on posterior base of soft dorsal fin; lateral body with alternating stripes formed from black and red spots *Lepomis miniatus*

14a. (11b) Opercle flap black to the posterior margin; opercle flap is thin near the opercle bone with the narrowest width of the flexible portion of the flap about the same diameter of the eye pupil *Lepomis auritus*

14b. (11b) Opercle flap black and surrounded by white on the posterior margin; opercle flap is wide with narrowest width of the flexible flap is about two times the diameter of the eye pupil *Lepomis megalotis*

FAMILY PERCIDAE—perches

1a. Snout conical, extends beyond upper lip; body with ≥ 14 black vertical bars 2

1b. Snout less conical, does not extend beyond upper lip; body with < 14 black vertical bars or with a pattern other than vertical bars 3

2a. (1a) Body with thick vertical bars, bars alternate in length from long to short; 9 to 10 long bars *Percina carbonaria*

2b. (1a) Body with 14 to 16 thin vertical bars of similar length *Percina macrolepida*

3a. (1b) Sides of body with large black blotches; midline of abdomen naked or with enlarged scales *Percina sciera*

3b. (1b) Sides of body without large black blotches; scales on abdomen normal 4

4a. (3b) Lateral line short, < 6 pored scales; single row of horizontal dashes present *Etheostoma proeliare*

4b. (3b) Lateral line long (complete or incomplete), > 6 pored scales; if horizontal dashes present, accompanied by vertical bars 5

5a. (4b) Lateral line arched upward *Etheostoma gracile*

5b. (4b) Lateral line straight 6

6a. (5b) Lateral body with distinct series of M-shaped pigments; snout rounded and blunt *Etheostoma chlorosoma*

6b. (5b) Lateral body without distinct series of M-shaped pigments; snout not noticeably rounded and blunt 7

7a. (6b) Lateral region with mottling bisected by a light colored lateral stripe *Etheostoma parvipinne*

7b. (6b) Lateral region without mottling bisected by a light colored lateral stripe 8

8a. (7b) Anterior portion of lateral region with black horizontal dashes and posterior portion of lateral region with vertical bars; uninterrupted supratemporal canal; throat of live males orange; no reddish orange spots on sides *Etheostoma spectabile*

8b. (7b) Lateral region with vertical bars, horizontal dashes on anterior portion sometimes visible, but obscured by vertical bars; interrupted supratemporal canal; throat of live males blue or green; sides of live males scattered with reddish orange spots *Etheostoma lepidum*

FAMILY SCIAENIDAE—drums

*Aplodinotus grunniens*

FAMILY CICHLIDAE—cichlids

1a. Anal fin spines 5 to 6 *Herichthys cyanoguttatus*

1b. Anal fin spines < 5 (usually 3) *Oreochromis aureus*

FAMILY GOBIIDAE—gobies

*Gobiosoma bosc*

FAMILY ACHIRIDAE—American soles

*Trinectes maculatus*

**GUADALUPE AND SAN ANTONIO RIVER BASINS**

KEY TO THE FAMILIES

1a. Both eyes on one side of head; without right pectoral fin American Soles – Achiridae

1b. One eye on either side of head; with both pectoral fins 2

2a. (1a) Body without pelvic fins 3

2b. (1a) Body with pelvic fins 4

3a. (2a) Dorsal fin attached to caudal fin Freshwater Eels – Anguillidae

3b. (2a) Dorsal fin not attached to caudal fin Pipefishes – Syngnathidae

4a. (2b) Caudal fin heterocercal or abbreviated heterocercal Gars – Lepiososteidae

4b. (2b) Caudal fin homocercal 5

5a. (4b) One dorsal fin; pelvic fins without uniserial spines 6

5b. (4b) One or two dorsal fins; pelvic fins with uniserial spines 15

6a. (5a) With adipose fin 7

6b. (5a) Without adipose fin 10

7a. (4a) Without barbels 8

7b. (4a) With barbels 9

8a. (6a) Scales large, < 50 lateral line scales; incisor teeth present Tetras – Characidae

8b. (6a) Scales small, > 60 lateral line scales; incisor teeth absent Trouts – Salmonidae

9a. (7b) Body covered with bony plates; head with one pair of barbels Armored Catfishes – Loricariidae

9b. (7b) Scales absent; head with four to eight barbels Bullhead Catfishes – Ictaluridae

10a. (6b) Long anal fin with ≥ 17 fin rays Shads – Clupiedae

10b. (6b) Short anal fin with ≤ 13 rays 11

11a. (10b) Caudal fin forked or emarginated; lateral line usually present 12

11b. (10b) Caudal fin truncated or rounded; lateral line usually absent 13

12a. (11a) Inferior, fleshy mouth modified for sucking; > 7 pharyngeal teeth in main row, usually ≥ 10 dorsal fin rays Suckers – Catostomidae

12b. (11a) Mouth usually not fleshy or modified for sucking; < 7 pharyngeal teeth in main row, usually ≤ 10 dorsal fin rays Carps and Minnows – Cyprinidae

13a. (11b) Mature males with rounded anal fin; males and females with 3rd anal fin ray branched, no gonopodium present 14

13b. (11b) Mature males with pointed anal fin forming a gonopodium; males and females with 3rd anal fin ray unbranched Livebearers – Poeciliidae

14a. (13a) Body robust; teeth in single row are incisor-like and tricuspid (three points on a tooth) Pupfishes – Cyprinodontidae

14b. (13a) Body elongate; conical (cone-shaped) pointed teeth in a single row or several rows Killifishes – Fundulidae

15a. (5b) Pelvic fin position abdominal or sub-thoracic; dorsal fins widely separated 16

15b. (5b) Pelvic fin position thoracic; dorsal fins joined or, if separate, closely adjacent to one another 17

16a. (15a) Dorsal fin with 4 thick spines; anal fin with 2-3 spines; adipose eyelids present Mullets – Mugilidae

16b. (15a) Dorsal fin with 4 to 8 thin spines; anal fin with 1 spine; adipose eyelids absent Silversides – Atherinopsinidae

17a. (15b) One nostril (nare) on each side of head; lateral line interrupted Cichlids – Cichlidae

17b. (15b) Two nostrils (nares) on each side of head; lateral line complete, incomplete, or absent 18

18a. (17b) Dorsal fin with > 23 fin rays; lateral line extends to tip of caudal fin Drums – Sciaenidae

18b. (17b) Dorsal fin with < 23 fin rays; lateral line, if present, does not extend to tip of caudal fin 19

19a. (17b) Anal fin with 1 to 2 spines Perches – Percidae

19b. (17b) Anal fin with 3 to 8 spines 20

20a. (19b) Posterior margin of operculum with a sharp spine; spiny and soft dorsal fin separate or only slightly connected; pseudobranchium present and exposed Temparate Basses – Moronidae

20b. (19b) Posterior margin of operculum without a sharp spine; spiny and soft dorsal fins connected or with deep notch; pseudobranchium covered or absent Sunfishes – Centrarchidae

KEY TO THE SPECIES

FAMILY LEPISOSTEIDAE—gars

1a. Large teeth in upper jaw in parallel rows on each side *Atractosteus spatula*

1b. Large teeth in upper jaw one row, although another non-parallel row might be present 2

2a. (1b) Beak long and narrow, least width goes about 12 to 20 times in length; width of beak at nostrils < eye diameter; snout > ⅔ of head length *Lepisosteus osseus*

2b. (1b) Beak short and blunt, least width goes about 5 to 7 times in length; width of beak at nostrils > eye diameter; snout < ⅔ of head length *Lepisosteus oculatus*

FAMILY ANGUILLIDAE—freshwater eels

*Anguilla rostrata*

FAMILY CLUPEIDAE—herrings

1a. Twenty-nine to 33 anal fin rays; mouth subterminal and below level of middle of eye; black

shoulder spot ≥ pupil of eye *Dorosoma cepedianum*

1b. Twenty-four to 28 anal fin rays; mouth terminal and at level of eye; black shoulder spot < pupil of eye *Dorosoma petenense*

FAMILY CYPRINIDAE - minnows

1a. More than 15 soft rays on dorsal fin; dorsal and anal fins each with a strong serrated spine 2

1b. Fewer than 10 soft rays on dorsal fin; dorsal and anal fins without spine 3

2a. (1a) Upper jaw with two pairs of barbels *Cyprinus carpio*

2b. (1a) Upper jaw without barbels *Carassius auratus*

3a. (1b) Anal fin near caudal fin, distance from snout to origin of anal fin is > 2.5 times the distance from origin of anal fin to base of caudal fin; pharyngeal teeth with prominent parallel grooves *Ctenopharyngodon idella*

3b. (1b) Anal fin not noticeably near caudal fin: distance from snout to origin of anal fin is < 2.5 times the distance from origin of anal fin to base of caudal fin; pharyngeal teeth without prominent parallel grooves 4

4a. (3b) Intestine wound spirally around swim bladder; keratinous ridge on lower jaw *Campostoma anomalum*

4b. (3b) Intestine not wound spirally around swim bladder; keratinous ridge of lower jaw hardly evident 5

5a. (4b) Abdomen behind pelvic fins with a fleshy keel lacking scales; lateral line greatly decurved, distance between anterior lateral line scale and ventral most lateral line scale is > 3 scales in height *Notemigonus crysoleucas*

5b. (4b) Abdomen behind pelvic fin with scales; lateral line not greatly decurved, lateral line descends < 3 scales ventrally from highest point 6

6a. (5b) With maxillary barbels, might be small and not observable without opening the mouth or with magnification *Macrhybopsis marconis*

6b. (5b) Without maxillary barbels 7

7a. (6b) Predorsal scales appear crowded, smaller than scales on lateral body or appear as

overlapping scales; black spot in the middle, anterior portion of the dorsal fin 8

7b. (6b) Predorsal scales not crowded; without black spot in the middle, anterior portion of

the dorsal fin 9

8a. (7a) Caudal spot, if distinct, continuous with mid-lateral stripe; lateral line incomplete; intestine long, more than twice the standard length. *Pimephales promelas*

8b. (7a) Caudal spot distinct from mid-lateral stripe; lateral line complete, intestine forming a short S-shaped loop *Pimephales vigilax*

9a. (7b) Long intestine in a flat coil; black mid-lateral stripe extends through eye to snout *Dionda nigrotaeniata*

9b. (7b) Short S-shaped intestine 10

10a. (9b) Moderately decurved lateral line; diamond-shaped scales; dark shoulder patch present; melanophores concentrated between rays of dorsal and anal fins 11

10b. (9b) Lateral line incomplete, complete-straight, or complete-slightly decurved; scales not noticeably diamond-shaped; without dark shoulder patch; melanophores concentrated along rays of dorsal and anal fins 13

11a. (10a) Terminal mouth *Cyprinella lutrensis*

11b. (10a) Sub-terminal mouth 12

12a. (11b) Caudal fin base with a large black spot, about size of eye *Cyprinella venusta*

12b. (11b) Caudal fin base without a large black spot *Cyprinella lepida*

13a. (10b) Distinct and separate black dash at base of dorsal fin; apparent when viewed from above; pharyngeal teeth count usually 0,4-4,0 14

13b. (10b) No distinct and separate black dash at base of dorsal fin; pharyngeal teeth count usually 1,4-4,1 or 2,4-4,2 16

14a. (13a) Lateral line scales markedly elevated (taller than wide) anteriorly, elevated scale height 2 to 5 times scale width 15

14b. (13a) Lateral line scales not markedly elevated anteriorly, scale height 1 to 2 times scale width *Notropis stramineus*

15a. (14a) Dorsal and lateral body with melanophores outlining scales; with pronounced black lateral stripe; dorsal fin height goes 2.1 or more times in pre-dorsal length; infraorbital canal complete *Notropis volucellus*

15b. (14a) Dorsal and lateral body with sparse melanophores; scales outlined with melanophores are rare; pre-dorsal black spot is prominent and distinct from mid-dorsal stripe; dorsal fin height goes 2.0 or fewer times in pre-dorsal length; infraorbital canal incomplete *Notropis buchanani*

16a. (13b) Depressed dorsal fin longer than head. *Hybopsis amnis*

16b. (13b) Depressed dorsal fin shorter than head 17

17a. (16a) Dorsal fin origin opposite or anterior to pelvic fin origin 18

17b. (16a) Dorsal fin origin posterior to pelvic fin origin 20

18a. (17a) Pharyngeal teeth 5-5; mouth small and almost vertical *Opsopoeodus emiliae*

18b. (17a) Pharyngeal teeth are 1,4-4,1 or 2,4-4,2; mouth large and oblique 19

19a. (18b) Usually 8 anal fin rays; inside of mouth with black melanophores; dorsal fin insertion is opposite to pelvic fin insertion *Notropis chalybaeus*

19b. (18b) Usually 7 anal fin rays; inside of mouth without black melanophores; dorsal fin insertion is anterior to pelvic fin insertion *Notropis texanus*

20a. (17b) 32 – 36 lateral line scales; melanophores concentrated on upper and lower jaws; predorsal scales are not crowded *Notropis amabilis*

20b. (17b) 41 – 45 lateral line scales; melanophores scattered on chin; crowded predorsal scales *Lythrurus fumeus*

FAMILY CATOSTOMIDAE—suckers

1a. Dorsal fin long, base > than ⅓ of standard length; 22 to 30 dorsal fin rays 2

1b. Dorsal fin short, base < than ¼ of standard length; 4 to 18 dorsal fin rays 4

2a. (1a) Small scales, lateral line scales > 50; eye closer to back of head than to tip of snout;

head abruptly more slender than body; papillose lips *Cycleptus elongatus*

2b. (1a) Large scales, lateral line scales < 45; eye closer to tip of snout than back of head; plicate lips 3

3a. (2b) Subopercle triangular, broadest toward base; knob present at tip of lower lip; blunt snout, forming level with eye *Carpiodes carpio*

3b. (2b) Subopercle semicircular, broadest towards middle; knob absent at tip of lower lip rounded snout, forming below level of eye *Ictiobus bubalus*

4a. (1b) Lateral line complete and well developed; air bladder with 3 chambers *Moxostoma congestum*

4b. (1b) Lateral line absent in adults; air bladder with 2 chambers; color pattern (except in young with 2 dark stripes) consists of narrow vertical bars; back with crescentric scale marks *Erimyzon sucetta*

FAMILY CHARACIDAE—characins

*Astyanax mexicanus*

FAMILY ICTALURIDAE—bullhead catfishes

1a. Eyes absent; skin without pigment 2

1b. Eyes present; skin pigmented 3

2a. (1a) No teeth on jaws; lips at corner of mouth thin *Trogloglanis pattersoni*

2b. (1a) Well developed teeth on jaws; lips at corner of mouth thick *Satan eurystomus*

3a. (1b) Adipose fin joined to the caudal fin or separated by a shallow notch 4

3b. (1b) Adipose fin free at tip, not joined to caudal fin 5

4a. (3a) Mouth terminal; pectoral fin spine not serrated; lower lip and chin not heavily speckled with black pigment *Noturus gyrinus*

4b. (3a) Mouth sub-terminal; pectoral spine serrated; lower lip and chin heavily speckled with black pigment. *Noturus nocturnus*

5a. (3b) Head dorso-ventrally compressed; mouth terminal to superior *Pylodictis olivaris*

5b. (3b) Head rounded; mouth subterminal 6

6a. (5b) Caudal fin rounded or shallowly emarginate 7

6b. (5b) Caudal fin deeply forked 8

7a. (6a) Chin barbels completely or partially black; anal fin rays 17 to 24; anal fin broadly rounded *Ameiurus melas*

7b. (6a) Chin barbels white or yellow; anal fin rays 24 to 27; margin of anal fin generally straight *Ameiurus natalis*

8a. (6b) Anal fin rays 30 to 36; anal fin free margin is straight; medial keel-like ridge anterior to dorsal fin forms humped back appearance *Ictalurus furcatus*

8b. (6b) Anal fin rays 22 to 29; anal fin free margin is rounded; no humped back appearance 9

9a. (8b) Anal fin rays 27 to 29; pectoral fin spine goes < 5 times into standard length; random scattering of few black spots may be present *Ictalurus punctatus*

9b. (8b) Anal fin rays 22 to 26; pectoral fin spine goes > 5 times into standard length; diffuse black spots on sides *Ictalurus lupus*

FAMILY LORICARIIDAE—suckermouth catfishes

1a. Dorsal fin short with ≤ 9 rays *Hypostomus plecostomus*

1b. Dorsal fin long with ≥ 10 rays 2

2a. (1b) Light spots on a dark background *Pterygoplichthys anisitsi*

2b. (1b) Dark spots on a light background *Pterygoplichthys disjunctivus*

FAMILY SALMONIDAE—salmons

*Oncorhynchus mykiss*

FAMILY MUGILIDAE—mullets

1a. Lower jaw rounded, without a symphyseal knob; lower limb of 1st gill arch with 17 to 20 gill rakers; no adipose eyelid; scales ctenoid *Agonostomus monticola*

1b. Lower jaw angular, with a prominent symphyseal knob; lower limb of 1st gill arch with 25

to 60 gill rakers; adipose eyelid well developed in adults; scales cycloid in young, ctenoid in adults *Mugil cephalus*

FAMILY ATHERINOPSIDAE—New World silversides

1a. Scales ctenoid, rough to the touch; double pairs of black spots on dorsum; bases of dorsal and anal fin covered with scales *Membras martinica*

1b. Scales cycloid, smooth to the touch; dorsum with crosshatching, but not double pairs of black spots; bases of dorsal and anal fins not covered with scales; horizontal distance between spinous dorsal and anal fin origin less than 7% of standard length *Menidia audens*

FAMILY FUNDULIDAE—topminnows

1a. Distance from origin of dorsal fin to end of hypural plate < distance from origin of dorsal fin to preopercle or occasionally about equal to that distance; more than 30 longitudinal scale rows 2

1b. Distance from origin of dorsal fin to end of hypural plate > distance from origin of dorsal fin to preopercle; 30 or fewer longitudinal scale rows 3

2a. (1a) Body with a distinct black lateral band *Fundulus notatus*

2b. (1a) Body without a distinct black lateral band *Fundulus chrysotus*

3a. (1b) Conspicuous lateral stripe extending through eye to snout; body depth goes 4.5 to 5 times in standard length *Lucania goodei*

3b. (1b) No distinct lateral stripe; body depth goes 3.5 to 4 times in standard length *Lucania parva*

FAMILY CYPRINODONTIDAE— pupfishes

*Cyprinodon variegatus*

FAMILY POECILIIDAE—livebearers

1a. Origin of dorsal fin anterior to anal fin origin; intestinal canal long with many convolutions 2

1b. Origin of dorsal fin posterior to anal fin origin; intestinal canal short with few convolutions 5

2a. (1a) Teeth in single row; ventral rays of caudal fin in mature males extended to form swordlike extension *Xiphophorus hellerii*

2b. (1a) Teeth in villiform bands; caudal fin rays symmetrical 3

3a. (2b) Dorsal fin rays < 9 *Poecilia reticulata*

3b. (2b) Dorsal fin rays ≥10 4

4a. (3b) Dorsal fin rays 12 to 14; dorsal fin base more than ½ predorsal length; rows of dark spots on scales obscure diamond-shaped color pattern *Poecilia latipinna*

4b. (3b) Dorsal fin rays 10 to 12; dorsal fin base < ½ predorsal length; dark spots on scales do not obscure diamond-shaped color pattern; only exists as females *Poecilia formosa*

5a. (1b) Spines at tip of 3rd anal fin ray of male gonopodium (first enlarged ray) 1 to 3 times longer than wide *Gambusia affinis*

5b. (1b) Spines at tip of 3rd anal fin ray of male gonopodium 4 to 10 times longer than wide 6

6a. (5b) Distal segments of anterior branch of 4th fin ray of gonopodium coalesced to elbow; Extinct *Gambusia georgei*

6b. (5b) Distal segments of anterior branch of 4th fin ray of gonopodium not coalesced to elbow; postanal streak prominent (darker than markings on scale pockets); black markings on mouth; median row of spots on caudal fin; median row of spots on dorsal fin; terminal hook on 4th and 5th rays of gonopodium angular at tip *Gambusia geiseri*

FAMILY SYNGNATHIDAE—pipefishes

*Syngnathus scovelli*

FAMILY MORONIDAE—temperate basses

1a. Body depth goes < 3 times in standard length; teeth in single patch on back of tongue *Morone chrysops*

1b. Body depth goes > 3 times in standard length; teeth in 2 parallel patches on back of tongue *Morone saxatilis*

FAMILY CENTRARCHIDAE—sunfishes

1a. Five to 8 anal spines 2

1b. Three anal spines 4

2a. (1a) Eleven to 13 dorsal fin spines *Ambloplites rupestris*

2b. (1a) Six to 8 dorsal fin spines 3

2a. (1a) Dorsal fin set back on body, length of dorsal fin base < distance from its origin to posterior margin of eye; lateral body with wide to narrow dorsal black bands; ≤ 6 dorsal spines *Pomoxis annularis*

2b. (1a) Dorsal fin set forward on body, length of dorsal fin base equal to or greater than distance from its origin to posterior margin of eye; lateral body with checkerboard black and light pattern; ≥7 dorsal spines *Pomoxis nigromaculatus*

4a. (1b) Body slender, body depth contained > 3 times into standard length 5

4b. (1b) Body deep, body depth contained < 3 times into standard length 8

5a. (4a) Dorsal fins narrowly joined at base forming a deep notch; upper jaw extends past posterior margin of eye in adults; mid-lateral stripe generally complete, rows of spots ventral to mid-lateral stripe faint and incomplete *Micropterus salmoides*

5b. (4a) Dorsal fins broadly joined at base forming a shallow notch; upper jaw does not reach past posterior portion of eye; bases of soft dorsal and anal fins scaled 6

6a. (5b) No tooth patch on tongue; lower lateral region scales without black spots forming horizontal rows *Micropterus dolomieu*

6b. (5b) Tooth patch on tongue; lower lateral region scales with black spots forming horizontal rows 7

7a. (6b) Mid-lateral stripe often appears interrupted anteriorly, rows of spots ventral to mid-lateral stripe distinct and complete. *Micropterus punctulatus*

7b. (6b) Dark wide midlateral stripe present and disconnected anteriorly into a narrow midlateral stripe posteriorly, forming vertical bars *Micropterus treculii*

8a. (4b) Teeth on tongue; head and opercle with 3 to 5 distinct dark and light longitudinal stripes; red spot on posterior margin of opercle flap in fresh specimens *Lepomis gulosus*

8b. (4b) No teeth on tongue; head and opercle lacking distinct dark and light longitudinal stripes 9

9a. (8b) Pectoral fins long and pointed, reach anterior portion of eye or beyond when bent forward 10

9b. (8b) Pectoral fins short and rounded, do not reach past eye when bent forward 12

10a. (9a) Opercle flap stiff to its margin, posterior margin either red or orange in live specimens *Lepomis microlophus*

10b. (9a) Opercle flap flexible, posterior margin not red or orange in live specimens 11

11a. (10b) Opercle flap black to the margin; black spot on posterior base of soft dorsal fin *Lepomis macrochirus*

11b. (10b) Opercle flap outlined with thick white band; lacking black spot on posterior base of soft dorsal fin *Lepomis humilis*

12a. (9b) Black opercle flap stiff near the posterior margin with bone supporting all or majority of the flap 13

12b. (9b) Black opercle flap flexible near the posterior margin without bone supporting majority of the flap 14

13a. (12a) Body elongated with black spot on posterior base of soft dorsal fin *Lepomis cyanellus*

13b. (12a) Body rounded without black spot on posterior base of soft dorsal fin; lateral body with alternating stripes formed from black and red spots *Lepomis miniatus*

14a. (12b) Opercle flap black to the posterior margin; opercle flap is thin near the opercle bone with the narrowest width of the flexible portion of the flap about the same diameter of the eye pupil *Lepomis auritus*

14b. (12b) Opercle flap black and surrounded by white on the posterior margin; opercle flap is wide with narrowest width of the flexible flap is about two times the diameter of the eye pupil *Lepomis megalotis*

FAMILY PERCIDAE—perches

1a. Snout conical, extends beyond upper lip; body with ≥ 14 black vertical bars 2

1b. Snout less conical, does not extend beyond upper lip; body with < 14 black vertical bars or with a pattern other than vertical bars 3

2a. (1a) Body with thick vertical bars, bars alternate in length from long to short; 9 to 10 long bars *Percina carbonaria*

2b. (1a) Body with 14 to 16 thin vertical bars of similar length *Percina macrolepida*

3a. (1b) Sides of body with large black blotches; midline of abdomen naked or with enlarged scales 4

3b. (1b) Sides of body without large black blotches; scales on abdomen normal 5

4a. (3a) Upper lip connected to snout by a narrow frenum; blotches on sides of body are rectangle-shaped and bleed downward *Percina shumardi*

4b. (3a) Upper lip connected to snout by a broad frenum; blotches on sides of body do not bleed downward *Percina apristis*

5a. (3b) Lateral line short, < 6 pored scales; single row of horizontal dashes present *Etheostoma fonticola*

5b. (3b) Lateral line long (complete or incomplete), > 6 pored scales; if horizontal dashes present, accompanied by vertical bars 6

6a. (5b) Lateral line arched upward *Etheostoma gracile*

6b. (5b) Lateral line straight 7

7a. (6b) Lateral body with distinct series of M-shaped pigments; snout rounded and blunt *Etheostoma chlorosoma*

7b. (6b) Lateral body without distinct series of M-shaped pigments; snout not noticeably rounded and blunt 8

8a. (7b) Anterior portion of lateral region with black horizontal dashes and posterior portion of lateral region with vertical bars; uninterrupted supratemporal canal; throat of live males orange; no reddish orange spots on sides *Etheostoma spectabile*

8b. (7b) Lateral region with vertical bars, horizontal dashes on anterior portion sometimes visible, but obscured by vertical bars; interrupted supratemporal canal; throat of live males blue or green; sides of live males scattered with reddish orange spots *Etheostoma lepidum*

FAMILY SCIAENIDAE—drums

*Aplodinotus grunniens*

FAMILY CICHLIDAE—cichlids

1a. Anal fin spines 5 to 6 *Herichthys cyanoguttatus*

1b. Anal fin spines < 5 (usually 3) 2

2a. (1b) Gill rakers 14 to 20 (usually 17 to 18) on lower part of first gill arch; most teeth in outer row are unicuspid in adults; sides with 3 or 4 dark blotches or with no markings; no yellow on dorsal fin; caudal fin without distinct vertical stripes *Oreochromis mossambicus*

2b. (1b) Gill rakers 18 to 26 on lower part of 1st gill arch; outer row of teeth bicuspid in adults; caudal fin unmarked, or with vague, irregular dark markings, caudal fin often with a broad, red distal margin; young often with vertical bands on caudal fin *Oreochromis aureus*

FAMILY ACHIRIDAE—American soles

*Trinectes maculatus*

**NUECES RIVER BASIN**

KEY TO THE FAMILIES

1a. Both eyes on one side of head; without right pectoral fin American Soles – Achiridae

1b. One eye on either side of head; with both pectoral fins 2

1a. (1b) Body long and slender; without pelvic fins Freshwater Eels – Anguillidae

1b. (1b) Body truncated or elongated; with pelvic fins 2

3a. (2b) Caudal fin heterocercal or abbreviated heterocercal Gars – Lepiososteidae

3b. (2b) Caudal fin homocercal 4

4a. (3b) One dorsal fin; pelvic fins without uniserial spines 5

4b. (3b) One or two dorsal fins; pelvic fins with uniserial spines 11

5a. (4a) With adipose fin 6

5b. (4a) Without adipose fin 8

6a. (5a) Without barbels 7

6b. (5a) With barbels Bullhead Catfishes – Ictaluridae

7a. (6a) Scales large, < 50 lateral line scales; incisor teeth present Tetras – Characidae

7b. (6a) Scales small, > 60 lateral line scales; incisor teeth absent Trouts – Salmonidae

8a. (5b) Long anal fin with ≥ 17 fin rays Shads – Clupiedae

8b. (5b) Short anal fin with ≤ 13 rays 9

9a. (8b) Caudal fin forked or emarginated; lateral line usually present 10

9b. (8b) Caudal fin truncated or rounded; lateral line usually absent Livebearers – Poeciliidae

10a. (9a) Inferior, fleshy mouth modified for sucking; > 7 pharyngeal teeth in main row, usually ≥ 10 dorsal fin rays Suckers – Catostomidae

10b. (9a) Mouth usually not fleshy or modified for sucking; < 7 pharyngeal teeth in main row, usually ≤ 10 dorsal fin rays Carps and Minnows – Cyprinidae

11a. (4b) Pelvic fin position abdominal or sub-thoracic; dorsal fins widely separated 12

11b. (4b) Pelvic fin position thoracic; dorsal fins joined or, if separate, closely adjacent to one another 13

12a. (11a) Dorsal fin with 4 thick spines; anal fin with 2-3 spines; adipose eyelids present Mullets – Mugilidae

12b. (11a) Dorsal fin with 4 to 8 thin spines; anal fin with 1 spine; adipose eyelids absent Silversides – Atherinopsinidae

13a. (12b) One nostril (nare) on each side of head; lateral line interrupted Cichlids – Cichlidae

13b. (12b) Two nostrils (nares) on each side of head; lateral line complete, incomplete, or absent 14

14a. (15b) Dorsal fin with > 23 fin rays; lateral line extends to tip of caudal fin Drums – Sciaenidae

14b. (15b) Dorsal fin with < 23 fin rays; lateral line, if present, does not extend to tip of caudal fin 15

15a. (14b) Anal fin with 1 to 2 spines Perches – Percidae

15b. (14b) Anal fin with 3 to 8 spines 16

16a. (15b) Posterior margin of operculum with a sharp spine; spiny and soft dorsal fin separate or only slightly connected; pseudobranchium present and exposed Temperate Basses – Moronidae

16b. (15b) Posterior margin of operculum without a sharp spine; spiny and soft dorsal fins connected or with deep notch; pseudobranchium covered or absent Sunfishes – Centrarchidae

KEY TO THE SPECIES

FAMILY LEPISOSTEIDAE—gars

1a. Large teeth in upper jaw in parallel rows on each side *Atractosteus spatula*

1b. Large teeth in upper jaw one row, although another non-parallel row might be present 2

2a. (1b) Beak long and narrow, least width goes about 12 to 20 times in length; width of beak at nostrils < eye diameter; snout > ⅔ of head length *Lepisosteus osseus*

2b. (1b) Beak short and blunt, least width goes about 5 to 7 times in length; width of beak at nostrils > eye diameter; snout < ⅔ of head length *Lepisosteus oculatus*

FAMILY ANGUILLIDAE—freshwater eels

*Anguilla rostrata*

FAMILY CLUPEIDAE—herrings

1a. Twenty-nine to 33 anal fin rays; mouth subterminal and below level of middle of eye; black

shoulder spot ≥ pupil of eye *Dorosoma cepedianum*

1b. Twenty-four to 28 anal fin rays; mouth terminal and at level of eye; black shoulder spot < pupil of eye *Dorosoma petenense*

FAMILY CYPRINIDAE - minnows

1a. More than 15 soft rays on dorsal fin; dorsal and anal fins each with a strong serrated spine 2

1b. Fewer than 10 soft rays on dorsal fin; dorsal and anal fins without spine 3

2a. (1a) Upper jaw with two pairs of barbels *Cyprinus carpio*

2b. (1a) Upper jaw without barbels *Carassius auratus*

3a. (1b) Anal fin near caudal fin, distance from snout to origin of anal fin is > 2.5 times the distance from origin of anal fin to base of caudal fin; pharyngeal teeth with prominent parallel grooves *Ctenopharyngodon idella*

3b. (1b) Anal fin not noticeably near caudal fin: distance from snout to origin of anal fin is < 2.5 times the distance from origin of anal fin to base of caudal fin; pharyngeal teeth without prominent parallel grooves 4

4a. (3b) Intestine wound spirally around swim bladder; keratinous ridge on lower jaw *Campostoma anomalum*

4b. (3b) Intestine not wound spirally around swim bladder; keratinous ridge of lower jaw hardly evident 5

5a. (4b) Abdomen behind pelvic fins with a fleshy keel lacking scales; lateral line greatly decurved, distance between anterior lateral line scale and ventral most lateral line scale is > 3 scales in height *Notemigonus crysoleucas*

5b. (4b) Abdomen behind pelvic fin with scales; lateral line not greatly decurved, lateral line descends < 3 scales ventrally from highest point 6

6a. (5b) Predorsal scales appear crowded, smaller than scales on lateral body or appear as

overlapping scales; black spot in the middle, anterior portion of the dorsal fin 7

6b. (5b) Predorsal scales not crowded; without black spot in the middle, anterior portion of

the dorsal fin 8

7a. (6a) Caudal spot, if distinct, continuous with mid-lateral stripe; lateral line incomplete; intestine long, more than twice the standard length *Pimephales promelas*

7b. (6a) Caudal spot distinct from mid-lateral stripe; lateral line complete, intestine forming a short S-shaped loop *Pimephales vigilax*

8a. (6b) Long intestine in a flat coil *Dionda serena*

8b. (6b) Short S-shaped intestine 9

9a. (8b) Moderately decurved lateral line; diamond-shaped scales; dark shoulder patch present; melanophores concentrated between rays of dorsal and anal fins 10

9b. (8b) Lateral line incomplete, complete-straight, or complete-slightly decurved; scales not noticeably diamond-shaped; without dark shoulder patch; melanophores concentrated along rays of dorsal and anal fins 12

10a. (9a) Terminal mouth *Cyprinella lutrensis*

10b. (9a) Sub-terminal mouth 11

11a. (10b) Caudal fin base with a large black spot, about size of eye *Cyprinella venusta*

11b. (10b) Caudal fin base without a large black spot *Cyprinella lepida*

12a. (9b) Distinct and separate black dash at base of dorsal fin; apparent when viewed from above; pharyngeal teeth count usually 0,4-4,0 13

12b. (9b) No distinct and separate black dash at base of dorsal fin; pharyngeal teeth count usually 1,4-4,1 or 2,4-4,2 15

13a. (12a) Lateral line scales not markedly elevated anteriorly, scale height 1 to 2 times scale width *Notropis stramineus*

13b. (12a) Lateral line scales markedly elevated (taller than wide) anteriorly, elevated scale height 2 to 5 times scale width 14

14a. (13b) Dorsal and lateral body with melanophores outlining scales; with pronounced black lateral stripe; dorsal fin height goes 2.1 or more times in pre-dorsal length; infraorbital canal complete *Notropis volucellus*

14b. (13b) Dorsal and lateral body with sparse melanophores; scales outlined with melanophores are rare; pre-dorsal black spot is prominent and distinct from mid-dorsal stripe; dorsal fin height goes 2.0 or fewer times in pre-dorsal length; infraorbital canal incomplete *Notropis buchanani*

15a. (12b) Dorsal fin origin opposite or anterior to pelvic fin origin 16

15b. (12b) Dorsal fin origin posterior to pelvic fin origin *Notropis amabilis*

16a. (15a) Pharyngeal teeth 5-5; mouth small and almost vertical *Opsopoeodus emiliae*

16b. (15a) Pharyngeal teeth are 1,4-4,1 or 2,4-4,2; mouth large and oblique *Notropis texanus*

FAMILY CATOSTOMIDAE—suckers

1a. Dorsal fin long, base > than ⅓ of standard length; 22 to 30 dorsal fin rays 2

1b. Dorsal fin short, base < than ¼ of standard length; 4 to 18 dorsal fin rays *Moxostoma congestum*

2a. (1a) Small scales, lateral line scales > 50; eye closer to back of head than to tip of snout; head abruptly more slender than body; papillose lips *Cycleptus elongatus*

2b. (1a) Large scales, lateral line scales < 45; eye closer to tip of snout than back of head; plicate lips 3

3a. (2b) Subopercle triangular, broadest toward base; knob present at tip of lower lip; blunt snout, forming level with eye *Carpiodes carpio*

3b. (2b) Subopercle semicircular, broadest towards middle; knob present at tip of lower lip; rounded snout, forming below level of eye *Ictiobus bubalus*

FAMILY CHARACIDAE—characins

*Astyanax mexicanus*

FAMILY ICTALURIDAE—bullhead catfishes

1a. Adipose fin joined to the caudal fin or separated by a shallow notch *Noturus gyrinus*

1b. Adipose fin free at tip, not joined to caudal fin 2

2a. (1b) Head dorso-ventrally compressed; mouth terminal to superior *Pylodictis olivaris*

2b. (1b) Head rounded; mouth subterminal 3

3a. (2b) Caudal fin rounded or shallowly emarginate 4

3b. (2b) Caudal fin deeply forked 5

4a. (3a) Chin barbels completely or partially black; anal fin rays 17 to 24; anal fin broadly rounded *Ameiurus melas*

4b. (3a) Chin barbels white or yellow; anal fin rays 24 to 27; margin of anal fin generally straight *Ameiurus natalis*

5a. (3b) Anal fin rays 30 to 36; anal fin free margin is straight; medial keel-like ridge anterior to dorsal fin forms humped back appearance *Ictalurus furcatus*

5b. (3b) Anal fin rays 22 to 29; anal fin free margin is rounded; no humped back appearance 6

6a. (5b) Anal fin rays 27 to 29; pectoral fin spine goes < 5 times into standard length; random scattering of few black spots may be present *Ictalurus punctatus*

6b. (5b) Anal fin rays 22 to 26; pectoral fin spine goes > 5 times into standard length; diffuse black spots on sides *Ictalurus lupus*

FAMILY SALMONIDAE—salmons

*Oncorhynchus mykiss*

FAMILY MUGILIDAE—mullets

1a. Lower jaw rounded, without a symphyseal knob; lower limb of 1st gill arch with 17 to 20 gill rakers; no adipose eyelid; scales ctenoid *Agonostomus monticola*

1b. Lower jaw angular, with a prominent symphyseal knob; lower limb of 1st gill arch with 25

to 60 gill rakers; adipose eyelid well developed in adults; scales cycloid in young, ctenoid in adults *Mugil cephalus*

FAMILY ATHERINOPSIDAE—New World silversides

*Menidia audens*

FAMILY FUNDULIDAE—topminnows

*Fundulus notatus*

FAMILY POECILIIDAE—livebearers

1a. Origin of dorsal fin anterior to anal fin origin; intestinal canal long with many convolutions 2

1b. Origin of dorsal fin posterior to anal fin origin; intestinal canal short with few convolutions

*Gambusia affinis*

2a. (1a) Dorsal fin rays 12 to 14; dorsal fin base more than ½ predorsal length; rows of dark spots on scales obscure diamond-shaped color pattern *Poecilia latipinna*

2b. (1a) Dorsal fin rays 10 to 12; dorsal fin base < ½ predorsal length; dark spots on scales do not obscure diamond-shaped color pattern; only exists as females *Poecilia formosa*

FAMILY MORONIDAE—temperate basses

1a. Body depth goes < 3 times in standard length; teeth in single patch on back of tongue *Morone chrysops*

1b. Body depth goes > 3 times in standard length; teeth in 2 parallel patches on back of tongue *Morone saxatilis*

FAMILY CENTRARCHIDAE—sunfishes

1a. Five to 8 anal spines 2

1b. Three anal spines 3

2a. (1a) Dorsal fin set back on body, length of dorsal fin base < distance from its origin to posterior margin of eye; lateral body with wide to narrow dorsal black bands; ≤ 6 dorsal spines *Pomoxis annularis*

2b. (1a) Dorsal fin set forward on body, length of dorsal fin base equal to or greater than distance from its origin to posterior margin of eye; lateral body with checkerboard black and light pattern; ≥7 dorsal spines *Pomoxis nigromaculatus*

3a. (1b) Body slender, body depth contained > 3 times into standard length 4

3b. (1b) Body deep, body depth contained < 3 times into standard length 6

4a. (3a) Dorsal fins narrowly joined at base forming a deep notch; upper jaw extends past posterior margin of eye in adults; mid-lateral stripe generally complete, rows of spots ventral to mid-lateral stripe faint and incomplete *Micropterus salmoides*

4b. (3a) Dorsal fins broadly joined at base forming a shallow notch; upper jaw does not reach past posterior portion of eye; bases of soft dorsal and anal fins scaled 5

5a. (4b) No tooth patch on tongue; lower lateral region scales without black spots forming horizontal rows. *Micropterus dolomieu*

5b. (4b) Tooth patch on tongue; lower lateral region scales with black spots forming horizontal rows *Micropterus treculii*

6a. (3b) Teeth on tongue; head and opercle with 3 to 5 distinct dark and light longitudinal stripes; red spot on posterior margin of opercle flap in fresh specimens *Lepomis gulosus*

6b. (3b) No teeth on tongue; head and opercle lacking distinct dark and light longitudinal stripes 7

7a. (6b) Pectoral fins long and pointed, reach anterior portion of eye or beyond when bent forward 8

7b. (6b) Pectoral fins short and rounded, do not reach past eye when bent forward 10

8a. (7a) Opercle flap stiff to its margin, posterior margin either red or orange in live specimens *Lepomis microlophus*

8b. (7a) Opercle flap flexible, posterior margin not red or orange in live specimens 9

9a. (8b) Opercle flap black to the margin; black spot on posterior base of soft dorsal fin *Lepomis macrochirus*

9b. (8b) Opercle flap outlined with thick white band; lacking black spot on posterior base of soft dorsal fin *Lepomis humilis*

10a. (7b) Black opercle flap stiff near the posterior margin with bone supporting all or majority of the flap 11

10b. (7b) Black opercle flap flexible near the posterior margin without bone supporting majority of the flap 12

11a. (10a) Body elongated with black spot on posterior base of soft dorsal fin *Lepomis cyanellus*

11b. (10a) Body rounded without black spot on posterior base of soft dorsal fin; lateral body with alternating stripes formed from black and red spots *Lepomis miniatus*

12a. (10b) Opercle flap black to the posterior margin; opercle flap is thin near the opercle bone with the narrowest width of the flexible portion of the flap about the same diameter of the eye pupil *Lepomis auritus*

12b. (10b) Opercle flap black and surrounded by white on the posterior margin; opercle flap is wide with narrowest width of the flexible flap is about two times the diameter of the eye pupil *Lepomis megalotis*

FAMILY PERCIDAE—perches

1a. Snout conical, extends beyond upper lip; body with ≥ 14 black vertical bars; bars contain medial constrictions, giving them an hourglass shape *Percina carbonaria*

1b. Snout less conical, does not extend beyond upper lip; body with < 14 black vertical bars or with a pattern other than vertical bars 2

2a. (1b) Lateral line arched upward; lateral region may contain verticle 8-10 green vertical bars *Etheostoma gracile*

2b. (1b) Lateral line straight; lateral region with 8 to 13 vertical bars *Etheostoma lepidum*

FAMILY SCIAENIDAE—drums

*Aplodinotus grunniens*

FAMILY CICHLIDAE—cichlids

1a. Anal fin spines 5 to 6 *Herichthys cyanoguttatus*

1b. Anal fin spines < 5 (usually 3) 2

2a. (1b) Gill rakers 14 to 20 (usually 17 to 18) on lower part of first gill arch; most teeth in outer row are unicuspid in adults; sides with 3 or 4 dark blotches or with no markings; no yellow on dorsal fin; caudal fin without distinct vertical stripes *Oreochromis mossambicus*

2b. (1b) Gill rakers 18 to 26 on lower part of 1st gill arch; outer row of teeth bicuspid in adults; caudal fin unmarked, or with vague, irregular dark markings, caudal fin often with a broad, red distal margin; young often with vertical bands on caudal fin *Oreochromis aureus*

FAMILY ACHIRIDAE—American soles

*Trinectes maculatus*

**RED RIVER BASIN**

KEY TO THE FAMILIES

1a. Jawless, disc-shaped mouth; without pectoral and pelvic fins; 7 pairs of external gill openings Lampreys – Petromyzontidae

1b. Jawed mouth; one gill opening on each side of head; with pectoral, pelvic, or both fins 2

2a. (1b) Body long and slender; without pelvic fins Freshwater Eels – Anguillidae

2b. (1b) Body truncated or elongated; with pelvic fins 3

3a. (2b) Caudal fin heterocercal or abbreviated heterocercal 4

3b. (2b) Caudal fin homocercal 7

4a. (3a) Caudal fin heterocercal, body with bony scutes or appears scaleless 5

4b. (3a) Caudal fin abbreviated heterocercal; body with ganoid or cycloid scales 6

5a. (4a) Long, paddle shaped snout; scaleless, except for a few ganoid scales at the base of caudal fin Paddlefishes – Polyodontidae

5b. (4a) Snout conical or shovel-shaped with four barbels on ventral surface; several rows of bony scutes (plates) along body Sturgeons – Acipenseridae

6a. (4b) Body covered with ganoid scales; snout formed into a beak; without gular plate Gars – Lepisosteidae

6b. (4b) Body covered with cycloid scales, snout not formed into a beak, with a gular plate Bowfin – Amiidae

7a. (3b) Jaws duckbilled Pickerels – Esocidae

7b. (3b) Jaws not duckbilled 8

8a. (7b) One dorsal fin; pelvic fins without uniserial spines 9

8b. (7b) One or two dorsal fins; pelvic fins with uniserial spines 18

9a. (8a) With adipose fin 10

9b. (8a) Without adipose fin 12

10a. (9a) Without barbels 11

10b. (9a) With barbels Bullhead Catfishes – Ictaluridae

11a. (10a) Scales large, < 50 lateral line scales; incisor teeth present Tetras – Characidae

11b. (10a) Scales small, > 60 lateral line scales; incisor teeth absent Trouts – Salmonidae

12a. (9b) Long anal fin with ≥ 17 fin rays 13

12b. (9b) Short anal fin with ≤ 13 rays 14

13a. (12a) Belly with scales forming a saw-like keel Shads – Clupiedae

13b. (12a) Belly without scales forming a saw-like keel Mooneyes – Hiodontidae

14a. (12b) Caudal fin forked or emarginated; lateral line usually present 15

14b. (12b) Caudal fin truncated or rounded; lateral line usually absent 16

15a. (14a) Inferior, fleshy mouth modified for sucking; > 7 pharyngeal teeth in main row, usually ≥ 10 dorsal fin rays Suckers – Catostomidae

15b. (14a) Mouth usually not fleshy or modified for sucking; < 7 pharyngeal teeth in main row, usually ≤ 10 dorsal fin rays Carps and Minnows – Cyprinidae

16a. (14b) Mature males with rounded anal fin; males and females with 3rd anal fin ray branched, no gonopodium present 17

16b. (14b) Mature males with pointed anal fin forming a gonopodium; males and females with 3rd anal fin ray unbranched. Livebearers – Poeciliidae

17a. (16a) Body robust; teeth in single row are incisor-like and tricuspid (three points on a tooth). Pupfishes – Cyprinodontidae

17b. (16a) Body elongate; conical (cone-shaped) pointed teeth in a single row or several rows Killifishes – Fundulidae

18a. (8b) Anus anterior to pelvic fins; > 5 soft rays on each pelvic fin Pirate Perch – Aphredoderidae

18b. (8b) Anus posterior to pelvic fins; 5 soft rays on pelvic fins 19

19a. (18b) Pelvic fin position abdominal or sub-thoracic; dorsal fins widely separated 20

19b. (18b) Pelvic fin position thoracic; dorsal fins joined or, if separate, closely adjacent to one another 21

20a. (19a) Dorsal fin with 4 thick spines; anal fin with 2-3 spines; adipose eyelids present Mullets – Mugilidae

20b. (19a) Dorsal fin with 4 to 8 thin spines; anal fin with 1 spine; adipose eyelids absent Silversides – Atherinopsidae

21a. (19b) Dorsal fin with > 23 fin rays; lateral line extends to tip of caudal fin Drums – Sciaenidae

21b. (19b) Dorsal fin with < 23 fin rays; lateral line, if present, does not extend to tip of caudal fin 22

22a. (21b) Anal fin with 1 to 2 spines Perches – Percidae

22b. (21b) Anal fin with 3 to 8 spines 23

23a. (22b) Posterior margin of operculum with a sharp spine; spiny and soft dorsal fin separate or only slightly connected; pseudobranchium present and exposed Temperate Basses – Moronidae

23b. (22b) Posterior margin of operculum without a sharp spine; spiny and soft dorsal fins connected or with deep notch; pseudobranchium covered or absent 24

24a. (23b) Lateral line present or incomplete Sunfishes – Centrarchidae

24b. (23b) Lateral line absent Pygmy Sunfishes – Elassomatidae

KEY TO THE SPECIES

FAMILY PETROMYZONTIDAE – lampreys

1a. Disc-shaped mouth large, diameter of mouth > body width and about 140 times into total length; with rasping teeth; adults with well-developed intestine *Ichthyomyzon castaneus*

1b. Disc-shaped mouth small, diameter of mouth ≤ body width and about 170 to 250 times into total length; without rasping teeth; adults without well-developed intestine *Ichthyomyzon gagei*

FAMILY ACIPENSERIDAE—sturgeons

*Scaphirhynchus platorynchus*

FAMILY POLYODONTIDAE—paddlefishes

*Polyodon spathula*

FAMILY LEPISOSTEIDAE—gars

1a. Large teeth in upper jaw in parallel rows on each side *Atractosteus spatula*

1b. Large teeth in upper jaw one row, although another non-parallel row might be present 2

2a. (1b) Beak long and narrow, least width goes about 12 to 20 times in length; width of beak at nostrils < eye diameter; snout > ⅔ of head length *Lepisosteus osseus*

2b. (1b) Beak short and blunt, least width goes about 5 to 7 times in length; width of beak at nostrils > eye diameter; snout < ⅔ of head length 3

3a. (2b) Fifty-nine to 63 lateral line scales; 38 to 44 scale rows around body, lacking black spots on head *Lepisosteus platostomus*

3b. (2b) Fifty-four to 57 lateral line scales; 32 to 38 scale rows around body; with black spots on head *Lepisosteus oculatus*

FAMILY AMIIDAE—bowfins

*Amia calva*

FAMILY HIODONTIDAE—mooneyes

*Hiodon alosoides*

FAMILY ANGUILLIDAE—freshwater eels

*Anguilla rostrata*

FAMILY CLUPEIDAE—herrings

1a. Twenty-nine to 33 anal fin rays; mouth subterminal and below level of middle of eye; black

shoulder spot ≥ pupil of eye *Dorosoma cepedianum*

1b. Twenty-four to 28 anal fin rays; mouth terminal and at level of eye; black shoulder spot < pupil of eye *Dorosoma petenense*

FAMILY CYPRINIDAE - minnows

1a. More than 15 soft rays on dorsal fin; dorsal and anal fins each with a strong serrated spine 2

1b. Fewer than 10 soft rays on dorsal fin; dorsal and anal fins without spine 3

2a. (1a) Upper jaw with two pairs of barbels *Cyprinus carpio*

2b. (1a) Upper jaw without barbels *Carassius auratus*

3a. (1b) Middle of eye is noticeably low on head, ventral to head midline *Hypophthalmichthys nobilis*

3b. (1b) Middle of eye is not noticeably low on head, equal or dorsal to head midline 4

4a. (3b) Anal fin near caudal fin, distance from snout to origin of anal fin is > 2.5 times the distance from origin of anal fin to base of caudal fin; pharyngeal teeth with prominent parallel grooves *Ctenopharyngodon idella*

4b. (3b) Anal fin not noticeably near caudal fin: distance from snout to origin of anal fin is < 2.5 times the distance from origin of anal fin to base of caudal fin; pharyngeal teeth without prominent parallel grooves 5

5a. (4b) Intestine wound spirally around swim bladder; keratinous ridge on lower jaw *Campostoma anomalum*

5b. (4b) Intestine not wound spirally around swim bladder; keratinous ridge of lower jaw hardly evident 6

6a. (5b) Abdomen behind pelvic fins with a fleshy keel lacking scales; lateral line greatly decurved, distance between anterior lateral line scale and ventral most lateral line scale is > 3 scales in height *Notemigonus crysoleucas*

6b. (5b) Abdomen behind pelvic fin with scales; lateral line not greatly decurved, lateral line descends < 3 scales ventrally from highest point 7

7a. (6b) With maxillary barbels, might be small and not observable without opening the mouth or with magnification 8

7b. (6b) Without maxillary barbels 11

8a. (7a) Mouth terminal; distinct black spot located anteriorly on dorsal fin; pharyngeal teeth on main row 5-4 or 5-5 *Semotilus atromaculatus*

8b. (7a) Mouth subterminal or inferior; no distinct black spot on dorsal fin; pharyngeal teeth on main row 4-4 9

9a. (8b) Body silvery, without scattered black specks *Macrhybopsis storeriana*

9b. (8b) Body with scattered black specks 10

10a. (9b) Two pairs of barbels; posterior barbels longer than orbit length; anterior barbels usually

half of orbit length *Macrhybopsis australis*

10b. (9b) One or 2 pairs of barbels; posterior barbels less than orbit length; anterior barbels, if

present, < half of posterior barbel length *Macrhybopsis hyostoma*

11a. (7b) Thick lower lip at corners, mouth noticeably ventral; black spot at base of caudal fin *Phenacobius mirabilis*

11b. (7b) Lower lip thin or not noticeably thick; with or without black spot at base of caudal fin 12

12a. (11b) Predorsal scales appear crowded, smaller than scales on lateral body or appear as

overlapping scales; black spot in the middle, anterior portion of the dorsal fin 13

12b. (11b) Predorsal scales not crowded; without black spot in the middle, anterior portion of the dorsal fin 14

13a. (12a) Caudal spot, if distinct, continuous with mid-lateral stripe; lateral line incomplete; intestine long, more than twice the standard length. *Pimephales promelas*

13b. (12a) Caudal spot distinct from mid-lateral stripe; lateral line complete, intestine forming a short S-shaped loop *Pimephales vigilax*

14a. (12b) Long intestine in a flat coil 15

14b. (12b) Short S-shaped intestine 17

15a. (14a) Terminal mouth; scales are diamond-shaped, most noticeable dorsally *Hybognathus hayi*

15b. (14a) Sub-terminal mouth; scales not diamond-shaped 16

16a. (15b) Head width greater than distance from tip of snout to posterior margin of orbital; internal posterior basiocciptial process is narrow and peg-like, width of internal posterior basiocciptal process fits in to head width at occipital > 7 times *Hybognathus placitus*

16b. (15b) Head width about equal to distance from tip of snout to posterior margin of orbital; internal posterior basiocciptial process is wide and flat, width of internal posterior basioccipital process fits in to head width at occipital < 7 times *Hybognathus nuchalis*

17a. (14b) Moderately decurved lateral line; diamond-shaped scales; dark shoulder patch present; melanophores concentrated between rays of dorsal and anal fins 18

17b. (14b) Lateral line incomplete, complete-straight, or complete-slightly decurved; scales not noticeably diamond-shaped; without dark shoulder patch; melanophores concentrated along rays of dorsal and anal fins 19

18a. (17a) Caudal fin base with a large black spot, about size of eye *Cyprinella venusta*

18b. (17a) Caudal fin base without a large black spot *Cyprinella lutrensis*

19a. (17b) Lateral line incomplete, < 11 pored scales 20

19b. (17b) Lateral line complete or mostly complete, > 11 pored scales 21

20a. (19a) Mouth terminal and oblique; dorsal fin origin posterior to pelvic fin origin; lacking smaller black spots above or below black spot at base of caudal fin *Pteronotropis hubbsi*

20b. (19a) Mouth sub-terminal and horizontal; dorsal fin origin in line with pelvic fin origin with smaller black spots above and below black spot at base of caudal fin *Notropis maculatus*

21a. (19b) Distinct and separate black dash at base of dorsal fin; apparent when viewed from above; pharyngeal teeth count usually 0,4-4,0 22

21b. (19b) No distinct and separate black dash at base of dorsal fin; pharyngeal teeth count usually 1,4-4,1 or 2,4-4,2 25

22a. (21a) Lateral line scales markedly elevated (taller than wide) anteriorly, elevated scale height 2 to 5 times scale width 23

22b. (21a) Lateral line scales not markedly elevated anteriorly, scale height 1 to 2 times scale width 24

23a. (22a) Dorsal and lateral body with melanophores outlining scales; with pronounced black lateral stripe; dorsal fin height goes 2.1 or more times in pre-dorsal length; infraorbital canal complete *Notropis volucellus*

23b. (22a) Dorsal and lateral body with sparse melanophores; scales outlined with melanophores are rare; pre-dorsal black spot is prominent and distinct from mid-dorsal stripe; dorsal fin height goes 2.0 or fewer times in pre-dorsal length; infraorbital canal incomplete *Notropis buchanani*

24a. (22b) Eye large, eye diameter is > snout length *Notropis stramineus*

24b. (22b) Eye small, eye diameter is < snout length *Notropis bairdi*

25a. (21b) Exposed portions of lateral line scales greatly elevated (taller than wide), elevated scale height 2 to 5 times scale width; dorsal scales with dark marking forming longitudinal stripes *Luxilus chrysocephalus*

25b. (21b) Lateral line scales not elevated, scale height 1 to 2 times scale width; dorsal scales to not form longitudinal stripes 26

26a. (25b) Depressed dorsal fin longer than head *Hybopsis amnis*

26b. (25b) Depressed dorsal fin shorter than head 27

27a. (26b) Mouth is sub-terminal; pharyngeal teeth are 0,4-4,0 *Notropis atrocaudalis*

27b. (26b) Mouth is terminal; pharyngeal teeth are 1,4-4,1 or 2,4-4,2 or 5-5 28

28a. (27b) Dorsal fin origin opposite or anterior to pelvic fin origin 29

28b. (27b) Dorsal fin origin posterior to pelvic fin origin 34

29a. (28a) Prominent mid-lateral stripe, extending through eye 30

29b. (28a) No prominent mid-lateral stripe present 32

30a. (29a) Pharyngeal teeth 5-5; mouth small and almost vertical *Opsopoeodus emiliae*

30b. (29a) Pharyngeal teeth are 1,4-4,1 or 2,4-4,2; mouth large and oblique 31

31a. (30b) Usually 8 anal fin rays; inside of mouth with black melanophores; dorsal fin insertion is opposite to pelvic fin insertion *Notropis chalybaeus*

31b. (30b) Usually 7 anal fin rays; inside of mouth without black melanophores; dorsal fin insertion is anterior to pelvic fin insertion *Notropis texanus*

32a. (29b) Usually 8 anal fin rays; head is narrow, depth at occiput more than width at occiput *Notropis shumardi*

32b. (29b) Usually 7 anal fin rays; head is wide, depth at occiput less than or equal to width at

occiput 33

33a. (32b) Mid-dorsal stripe about 5 chromatophores wide; snout overhanging mouth; middle portion of upper jaw narrower than ends *Notropis potteri*

33b. (32b) Mid-dorsal stripe about 10 chromatophores wide; snout not overhanging mouth; middle portion of upper jaw is about the same width as the ends *Notropis* *blennius*

34a. (28b) Small scales, ≥ 41 lateral line scales, > 25 predorsal scales 35

34b. (28b) Moderate-sized scales, ≤ 40 lateral line scales, < 24 predorsal scales *Notropis atherinoides*

35a. (34a) Dorsal fin with black melanophores extending from mid-dorsal stripe into the anterior fin ray; edges of the anterior dorsolateral scales are outlined with black melanophores, producing

chevron pattern. *Lythrurus umbratilis*

35b. (34a) Dorsal fin with black melanophores not extending from mid-dorsal stripe into the anterior fin ray, lacks chevron pattern *Lythrurus fumeus*

FAMILY CATOSTOMIDAE—suckers

1a. Dorsal fin long, base > than ⅓ of standard length; 22 to 30 dorsal fin rays 2

1b. Dorsal fin short, base < than ¼ of standard length; 4 to 18 dorsal fin rays 6

2a. (1a) Small scales, lateral line scales > 50; eye closer to back of head than to tip of snout;

head abruptly more slender than body; papillose lips *Cycleptus elongatus*

2b. (1a) Large scales, lateral line scales < 45; eye closer to tip of snout than back of head; plicate lips 3

3a. (2b) Subopercle triangular, broadest toward base; knob present at tip of lower lip; blunt snout, forming level with eye *Carpiodes carpio*

3b. (2b) Subopercle semicircular, broadest towards middle; knob absent at tip of lower lip; rounded snout, forming below level of eye 4

4a. (3b) Mouth large and oblique; upper jaw length is equal to snout length *Ictiobus cyprinellus*

4b. (3b) Mouth small and nearly horizontal; upper jaw shorter than snout 5

5a. (4b) Body elongate and slender, greatest body depth goes 2.6 to 3.3 times in standard length, and height of anterior rays in dorsal and anal fins often less than 2/3 head length in individuals >300 mm; small eye, eye diameter goes ≥ 2 times in snout length of individuals <300 mm *Ictiobus niger*

5b. (4b) Body deep and narrow, greatest body depth goes 2.2 to 2.8 times in standard length, and height of anterior dorsal and anal fin rays often greater than 2/3 head length in individuals >300 mm; large eye, eye diameter goes ≤ 2 times in snout length of individuals <300 mm *Ictiobus bubalus*

6a. (1b) Lateral line complete and well developed; air bladder with 3 chambers *Moxostoma erythrurum*

6b. (1b) Lateral line incomplete or absent; air bladder with 2 chambers 7

7a. (6b) Lateral line incomplete; rows of spots *Minytrema melanops*

7b. (6b) Lateral line absent 8

8a. (7b) Scales larger, lateral scale count 34 to 37; eye larger, eye length ½ of snout length; dorsal fin rays 11 or 12; back with crescentic scale marks *Erimyzon sucetta*

8b. (7b) Scales smaller, lateral scale count 39 to 43; eye smaller, eye length < ½ of snout length); dorsal fin rays 9 or 10; back without crescentic scale marks *Erimyzon claviformis*

FAMILY CHARACIDAE—characins

*Astyanax mexicanus*

FAMILY ICTALURIDAE—bullhead catfishes

1a. Adipose fin joined to the caudal fin or separated by a shallow notch 2

1b. Adipose fin free at tip, not joined to caudal fin 3

2a. (1a) Mouth terminal; pectoral fin spine not serrated; lower lip and chin not heavily speckled with black pigment *Noturus gyrinus*

2b. (1a) Mouth sub-terminal; pectoral spine serrated; lower lip and chin heavily speckled with black pigment. *Noturus nocturnus*

3a. (1b) Head dorso-ventrally compressed; mouth terminal to superior *Pylodictis olivaris*

3b. (1b) Head rounded; mouth subterminal 4

4a. (3b) Caudal fin rounded or shallowly emarginate 5

4b. (3b) Caudal fin deeply forked 7

5a. (4a) Chin barbels white or yellow; anal fin rays 24 to 27; margin of anal fin generally straight *Ameiurus natalis*

5b. (4a) Chin barbels completely or partially black; anal fin rays 17 to 24; anal fin broadly rounded 6

6a. (5b) Posterior margin of pectoral fin spine nearly smooth; anal fin rays 17 to 23; dorsal and lateral body uniformly dark *Ameiurus melas*

6b. (5b) Posterior margin of pectoral fin spine with serrations; anal fin rays 21 to 24; dorsal and lateral body mottled *Ameiurus nebulosus*

7a. (4b) Anal fin rays 30 to 36; anal fin free margin is straight; medial keel-like ridge anterior to dorsal fin forms humped back appearance *Ictalurus furcatus*

7b. (4b) Anal fin rays 22 to 29; anal fin free margin is rounded; no humped back appearance *Ictalurus punctatus*

FAMILY SALMONIDAE—salmons

*Oncorhynchus mykiss*

FAMILY ESOCIDAE—pikes and pickerels

1a. Snout short, distance from tip of snout to center of eye ≤ distance from center of eye to rear margin of operculum; < 115 scale rows along body *Esox americanus*

1b. Snout long, distance from tip of snout to center of eye > distance from center of eye to rear margin of operculum; > 120 scale rows along body *Esox niger*

FAMILY APHREDODERIDAE—pirate perch

*Aphredoderus sayanus*

FAMILY MUGILIDAE—mullets

*Mugil cephalus*

FAMILY ATHERINOPSIDAE—New World silversides

1a. Scales small, > 60 scales in lateral series, jaws produced into a short beak; snout length > eye length; > 20 anal fin rays *Labidesthes sicculus*

1b. Scales large, < 50 scales in lateral series; jaws not produced into a beak; snout length ≤ eye length; < 20 anal fin rays *Menidia audens*

FAMILY FUNDULIDAE—topminnows

1a. Lateral body with 15 to 17 prominent dark bars alternating with near equal width bands of white or yellow bars, dark bars less distinct in females; small scales, usually > 40 scales along lateral row *Fundulus zebrinus*

1b. Lateral body without 15 to 17 prominent dark bars, with spots or a prominent mid-lateral stripe; large scales, usually < 40 scales along lateral row 2

2a. (1b) Body with a distinct black lateral band 3

2b. (1b) Body without a distinct black lateral band 4

3a. (2a) Distinct black spots on anterior dorso-lateral region are as pronounced as lateral stripe; distinct black spots throughout dorsal and caudal fins *Fundulus olivaceus*

3b. (2a) Faint black spots on anterior dorso-lateral region are not as pronounced as lateral stripe; distinct black spots near base of dorsal and caudal fins *Fundulus notatus*

4a. (2b) Dorsal fin originating anterior to anal fin origin; more than 15 scale rows from pelvic fin origin to isthmus predorsal stripe absent or not reaching occiput *Fundulus grandis*

4b. (2b) Dorsal fin originating posterior to anal fin origin 5

5a. (4b) Red to dark spots in multiple rows longitudinally along lateral sides; usually with dark subocular bar *Fundulus blairae*

5b. (4b) Body mottled, barred or irregularly spotted; no dark subocular bar *Fundulus chrysotus*

FAMILY CYPRINODONTIDAE— pupfishes

*Cyprinodon rubrofluviatilis*

FAMILY POECILIIDAE—livebearers

*Gambusia affinis*

FAMILY MORONIDAE—temperate basses

1a. Dorsal fins united at base; 2nd and 3rd anal fin spines approximately equal in length; no teeth on tongue; 9 to 10 anal fin soft rays; stripes along sides usually sharply broken and offset above front of anal fin *Morone mississippiensis*

1b. Dorsal fins separated; 2nd anal fin spine much shorter than 3rd; base of tongue with teeth;

11 to 13 anal fin soft rays; stripes along sides usually continuous 2

2a. (1b) Body depth goes < 3 times in standard length; teeth in single patch on back of tongue *Morone chrysops*

2b. (1b) Body depth goes > 3 times in standard length; teeth in 2 parallel patches on back of tongue *Morone saxatilis*

FAMILY CENTRARCHIDAE—sunfishes

1a. Five to 8 anal spines 2

1b. Three anal spines 4

2a. (1a) Eleven to 13 dorsal fin spines *Centrarchus macropterus*

2b. (1a) Six to 8 dorsal fin spines 3

3a. (2b) Dorsal fin set back on body, length of dorsal fin base < distance from its origin to posterior margin of eye; lateral body with wide to narrow dorsal black bands; ≤ 6 dorsal spines *Pomoxis annularis*

3b. (2b) Dorsal fin set forward on body, length of dorsal fin base equal to or greater than distance from its origin to posterior margin of eye; lateral body with checkerboard black and light pattern; ≥7 dorsal spines *Pomoxis nigromaculatus*

4a. (1b) Body slender, body depth contained > 3 times into standard length 5

4b. (1b) Body deep, body depth contained < 3 times into standard length 7

5a. (4a) Dorsal fins narrowly joined at base forming a deep notch; upper jaw extends past posterior margin of eye in adults; mid-lateral stripe generally complete, rows of spots ventral to mid-lateral stripe faint and incomplete *Micropterus salmoides*

5b. (4a) Dorsal fins broadly joined at base forming a shallow notch; upper jaw does not reach past posterior portion of eye; bases of soft dorsal and anal fins scaled 6

6a. (5b) No tooth patch on tongue; lower lateral region scales without black spots forming horizontal rows *Micropterus dolomieu*

6b. (5b) Tooth patch on tongue; lower lateral region scales with black spots forming horizontal rows *Micropterus punctulatus*

7a. (4b) Teeth on tongue; head and opercle with 3 to 5 distinct dark longitudinal stripes; red spot on posterior margin of opercle flap in fresh specimens *Lepomis gulosus*

7b. (4b) No teeth on tongue; head and opercle lacking distinct dark longitudinal stipes 8

8a. (7b) Pectoral fins long and pointed, extending past anterior portion of eye or when bent forward 9

8b. (7b) Pectoral fins short and rounded, do not extend past eye when bent forward 11

9a. (7a) Opercle flap stiff to its margin, posterior margin either red or orange in live specimens *Lepomis microlophus*

9b. (7a) Opercle flap flexible, posterior margin not red or orange in live specimens 10

10a. (9b) Opercle flap black to the margin; black spot on posterior base of soft dorsal fin *Lepomis macrochirus*

10b. (9b) Opercle flap outlined with think white band; lacking black spot on posterior base of soft dorsal fin *Lepomis humilis*

11a. (8b) Black opercle flap stiff near the posterior margin with bone supporting all or majority of the flap 12

11b. (8b) Black opercle flap flexible near the posterior margin without bone supporting majority of the flap 14

12a. (11a) Lateral line incomplete; smaller individuals with black spot surrounded by white margin on posterior base of soft dorsal fin *Lepomis symmetricus*

12b. (11a) Lateral line complete; black spot, if present, on posterior base of soft dorsal fin without white margin 13

13a. (12b) Body elongated with black spot on posterior base of soft dorsal fin *Lepomis cyanellus*

13b. (12b) Body rounded without black spot on posterior base of soft dorsal fin; lateral body with alternating stripes formed from black and red spots *Lepomis miniatus*

14a. (11b) Opercle flap black to the posterior margin; opercle flap is thin near the opercle bone with the narrowest width of the flexible portion of the flap about the same diameter of the eye pupil *Lepomis auritus*

14b. (11b) Opercle flap black and surrounded by white on the posterior margin; opercle flap is wide with narrowest width of the flexible flap is about two times the diameter of the eye pupil 15

15a. (14b) Twelve pectoral fin rays, 3 to 5 cheek scales; opercle flap often with white pigment form speckles, distinct red spots (white in preserved specimens) along lateral line *Lepomis marginatus*

15b. (14b) Thirteen to 15 pectoral fin rays, 5 to 7 cheek scales; opercle flaps with red or white margin; 13 to 15 pectoral fin rays *Lepomis megalotis*

FAMILY PERCIDAE—perches

1a. Body depth contained in standard length more than 7 times 2

1b. Body depth contained in standard length less than 7 times .3

2a. (1a) Lateral blotches longer than deep *Ammocrypta clara*

2b. (1a) Lateral blotches deeper than long *Ammocrypta vivax*

3a. (1b) Snout conical, extends beyond upper lip; body with ≥ 14 black vertical bars 4

3b. (1b) Snout less conical, does not extend beyond upper lip; body with < 14 black vertical bars or with a pattern other than vertical bars 5

4a. (3a) Body with 14 to 16 thin vertical bars of similar length *Percina macrolepida*

4b. (3a) Body with thick vertical bars, bars alternate in length from long to short *Percina caprodes*

5a. (3b) Sides of body with large black blotches; midline of abdomen naked or with enlarged scales 6

5b. (3b) Sides of body without large black blotches; scales on abdomen normal 9

6a. (5a) Upper lip connected to snout by a narrow frenum; blotches on sides of body are rectangle-shaped and bleed downward *Percina shumardi*

6b. (5a) Upper lip connected to snout by a broad frenum; blotches on sides of body do not bleed downward 7

7a. (6b) Nape unscaled; blotches on sides of body are rectangle shaped and might appear connected *Percina maculata*

7b. (6b) Nape scaled; blotches on sides of body not rectangle shaped 8

8a. (7b) Sides of body with large, black heart-shaped blotches; preopercle with ≥ 5 serrations *Percina sciera*

8b. (7b) Sides of body with large diamond-shaped blotches; preopercle with 0 to 3 serrations *Percina phoxocephala*

9a. (5b) Lateral line short, < 6 pored lateral line scales; single row of horizontal dashes present *Etheostoma proeliare*

9b. (5b) Lateral line long, > 6 pored lateral line scales; if horizontal dashes present, accompanied by vertical bars 10

10a. (9b) Pectoral fin long, pectoral fin folded forward extends past head *Etheostoma histrio*

10b. (9b) Pectoral fin short, pectoral fin folded forward does not extend past 11

11a. (10b) Lateral line arched upward 12

11b. (10b) Lateral line straight 13

12a. (11a) Breast without scales; breast and abdomen without black speckles *Etheostoma gracile*

12b. (11a) Breast with scales; breast and abdomen with black speckles *Etheostoma fusiforme*

13a. (11b) Lateral body with distinct series of M-shaped pigments; snout rounded and blunt *Etheostoma chlorosoma*

13b. (11b) Lateral body without distinct series of M-shaped pigments; snout not noticeably rounded and blunt 14

14a. (13b) Lateral region with mottling bisected by a light colored lateral stripe *Etheostoma parvipinne*

14b. (13b) Lateral region without mottling bisected by a light colored lateral stripe 15

15a. (14b) Gill membranes widely joined across isthmus 16

15b. (14b) Gill membranes either not joined or barely joined across isthmus 17

16a. (15a) Lateral region with red or yellow spots *Etheostoma artesiae*

16b. (15a) Lateral region without red or yellow spots *Etheostoma radiosum*

17a. (15b) Cheek not scaled; infraorbital canal incomplete; lateral body with 8 to 9 vertical bars *Etheostoma spectabile*

17b. (15b) Cheek scaled; infraorbital canal complete; lateral body with 6 to 8 vertical bars *Etheostoma asprigene*

FAMILY SCIAENIDAE—drums

*Aplodinotus grunniens*

FAMILY ELASSOMATIDAE—pygmy sunfishes

*Elassoma zonatum*

**RIO GRANDE AND PECOS RIVER BASINS**

KEY TO THE FAMILIES

1a. Body long and slender; without pelvic fins Freshwater Eels – Anguillidae

1b. Body truncated or elongated; with pelvic fins 2

2a. (1b) Caudal fin heterocercal or abbreviated heterocercal Gars – Lepiososteidae

2b. (1b) Caudal fin homocercal 3

3a. (2b) One dorsal fin; pelvic fins without uniserial spines 4

3b. (2b) One or two dorsal fins; pelvic fins with uniserial spines 13

4a. (3a) With adipose fin 5

4b. (3a) Without adipose fin 8

5a. (4a) Without barbels 6

5b. (4a) With barbels 7

6a. (5a) Scales large, < 50 lateral line scales; incisor teeth present Tetras – Characidae

6b. (5a) Scales small, > 60 lateral line scales; incisor teeth absent Trouts – Salmonidae

7a. (5b) Body covered with bony plates; head with one pair of barbels Armored Catfishes – Loricariidae

7b. (5b) Scales absent; head with four to eight barbels Bullhead Catfishes – Ictaluridae

8a. (4b) Long anal fin with ≥ 17 fin rays Shads – Clupiedae

8b. (4b) Short anal fin with ≤ 13 rays 9

9a. (8b) Caudal fin forked or emarginated; lateral line usually present 10

9b. (8b) Caudal fin truncated or rounded; lateral line usually absent 11

10a. (9a) Inferior, fleshy mouth modified for sucking; > 7 pharyngeal teeth in main row, usually ≥ 10 dorsal fin rays Suckers – Catostomidae

10b. (9a) Mouth usually not fleshy or modified for sucking; < 7 pharyngeal teeth in main row, usually ≤ 10 dorsal fin rays Carps and Minnows – Cyprinidae

11a. (9b) Mature males with rounded anal fin; males and females with 3rd anal fin ray branched, no gonopodium present 12

11b. (9b) Mature males with pointed anal fin forming a gonopodium; males and females with 3rd anal fin ray unbranched. Livebearers – Poeciliidae

12a. (11a) Body robust; teeth in single row are incisor-like and tricuspid (three points on a tooth). Pupfishes – Cyprinodontidae

12b. (11a) Body elongate; conical (cone-shaped) pointed teeth in a single row or several rows Killifishes – Fundulidae

13a. (3b) Pelvic fin position abdominal or sub-thoracic; dorsal fins widely separated 14

13b. (3b) Pelvic fin position thoracic; dorsal fins joined or, if separate, closely adjacent to one another 15

14a. (13a) Dorsal fin with 4 thick spines; anal fin with 2-3 spines; adipose eyelids present Mullets – Mugilidae

14b. (13a) Dorsal fin with 4 to 8 thin spines; anal fin with 1 spine; adipose eyelids absent Silversides – Atherinopsidae

15a. (13b) One nostril (nare) on each side of head; lateral line interrupted Cichlids – Cichlidae

15b. (13b) Two nostrils (nares) on each side of head; lateral line complete, incomplete, or absent 16

16a. (15b) Dorsal fin with > 23 fin rays; lateral line extends to tip of caudal fin Drums – Sciaenidae

16b. (15b) Dorsal fin with < 23 fin rays; lateral line, if present, does not extend to tip of caudal fin 17

17a. (16b) Pelvic fins joined into a sucking disk, gill membranes broadly joined to isthmus Gobies – Gobiidae

17b. (16b) Pelvic fins not joined, gill membranes free or nearly free from isthmus (may be joined to each other across isthmus) 18

18a. (17b) Anal fin with 1 to 2 spines Perches – Percidae

18b. (17b) Anal fin with 3 to 8 spines 19

19a. (18b) Posterior margin of operculum with a sharp spine; spiny and soft dorsal fin separate or only slightly connected; pseudobranchium present and exposed Temparate Basses – Moronidae

19b. (18b) Posterior margin of operculum without a sharp spine; spiny and soft dorsal fins connected or with deep notch; pseudobranchium covered or absent Sunfishes – Centrarchidae

KEY TO THE SPECIES

FAMILY LEPISOSTEIDAE—gars

1a. Large teeth in upper jaw in parallel rows on each side *Atractosteus spatula*

1b. Large teeth in upper jaw one row, although another non-parallel row might be present 2

2a. (1b) Beak long and narrow, least width goes about 12 to 20 times in length; width of beak at nostrils < eye diameter; snout > ⅔ of head length *Lepisosteus osseus*

2b. (1b) Beak short and blunt, least width goes about 5 to 7 times in length; width of beak at nostrils > eye diameter; snout < ⅔ of head length *Lepisosteus oculatus*

FAMILY ANGUILLIDAE—freshwater eels

*Anguilla rostrata*

FAMILY CLUPEIDAE—herrings

1a. Twenty-nine to 33 anal fin rays; mouth subterminal and below level of middle of eye; black

shoulder spot ≥ pupil of eye *Dorosoma cepedianum*

1b. Twenty-four to 28 anal fin rays; mouth terminal and at level of eye; black shoulder spot < pupil of eye *Dorosoma petenense*

FAMILY CYPRINIDAE - minnows

1a. More than 15 soft rays on dorsal fin; dorsal and anal fins each with a strong serrated spine 2

1b. Fewer than 10 soft rays on dorsal fin; dorsal and anal fins without spine 3

2a. (1a) Upper jaw with two pairs of barbels *Cyprinus carpio*

2b. (1a) Upper jaw without barbels *Carassius auratus*

3a. (1b) Anal fin near caudal fin, distance from snout to origin of anal fin is > 2.5 times the distance from origin of anal fin to base of caudal fin; pharyngeal teeth with prominent parallel grooves *Ctenopharyngodon idella*

3b. (1b) Anal fin not noticeably near caudal fin: distance from snout to origin of anal fin is < 2.5 times the distance from origin of anal fin to base of caudal fin; pharyngeal teeth without prominent parallel grooves 4

4a. (3b) Intestine wound spirally around swim bladder; keratinous ridge on lower jaw 5

4b. (3b) Intestine not wound spirally around swim bladder; keratinous ridge of lower jaw hardly evident 6

5a. (4a) Intestine wound completely around swim bladder, large scales, 41 to 58 lateral line scales *Campostoma anomalum*

5b. (4a) Intestine only partially wound around swim bladder; 58 to 77 lateral line scales;

more common in the Presidio to Big Bend Reach of Rio Grande drainage *Campostoma ornatum*

6a. (4b) Upper jaw with frenum, dorsal part of premaxillary bones connected to frenum are not protractible *Rhinichthys cataractae*

6b. (4b) Upper jaw without frenum, premaxillary bones are protractible 7

7a. (6b) Abdomen behind pelvic fins with a fleshy keel lacking scales; lateral line greatly decurved, distance between anterior lateral line scale and ventral most lateral line scale is > 3 scales in height *Notemigonus crysoleucas*

7b. (6b) Abdomen behind pelvic fin with scales; lateral line not greatly decurved, lateral line descends < 3 scales ventrally from highest point 8

8a. (7b) With maxillary barbels, might be small and not observable without opening the mouth or

with magnification *Macrhybopsis aestivalis*

8b. (7b) Without maxillary barbels 9

9a. (8b) Small scales, ≥ 50 lateral line scales; current Texas distribution is Little Aguja Creek in the Rio Grande drainage *Gila pandora*

9b. (8b) Larger scales, < 45 lateral line scales 10

10a. (9b) Predorsal scales appear crowded, smaller than scales on lateral body or appear as

overlapping scales; black spot in the middle, anterior portion of the dorsal fin 11

10b. (9b) Predorsal scales not crowded; without black spot in the middle, anterior portion of

the dorsal fin 12

11a. (10a) Caudal spot, if distinct, continuous with mid-lateral stripe; lateral line incomplete; intestine long, more than twice the standard length. *Pimephales promelas*

11b. (10a) Caudal spot distinct from mid-lateral stripe; lateral line complete, intestine forming a short S-shaped loop *Pimephales vigilax*

12a. (10b) Long intestine in a flat coil 13

12b. (10b) Short S-shaped intestine 16

13a. (12a) Black mid-lateral stripe extends through eye to snout; eye width greater than or equal to snout length 14

13b. (12a) Mid-lateral stripe, if present (sometimes appears as a broad, diffuse band of melanophores), does not extend through the eye to the snout; eye width less than snout length *Hybognathus amarus*

14a. (13a) Scales outlined with melanophores, most noticeable dorsal of mid-lateral stripe, forming a cross-hatched appearance; more of a triangular shaped caudal spot; sympatric with *Dionda diaboli*

14b. (13a) Scales equally covered with melanophores; more of a rounded caudal spot 15

15a. (14b) Located (all water bodies) downstream from I-10 in the Pecos and within and downstream from Lake Amistad in the Rio Grande *Dionda argentosa*

15b. (14b) Complete description pending. Based on genetic analyses, located in Trans Pecos region (all water bodies) upstream from I-10 in the Pecos River and upstream from Lake Amistad in the Rio Grande. Not sympatric with other *Dionda* *Dionda episcopa*

16a. (12b) Moderately decurved lateral line; diamond-shaped scales; dark shoulder patch present; melanophores concentrated between rays of dorsal and anal fins 17

16b. (12b) Lateral line incomplete, complete-straight, or complete-slightly decurved; scales not noticeably diamond-shaped; without dark shoulder patch; melanophores concentrated along rays of dorsal and anal fins 19

17a. (16a) Terminal mouth *Cyprinella lutrensis*

17b. (16a) Sub-terminal mouth 18

18a. (17b) A thick distinct black bar extends from lower jaw through isthmus; caudal fin base without a large black spot *Cyprinella proserpina*

18b. (17b) Caudal fin base with a large black spot, about size of eye *Cyprinella venusta*

19a. (16b) Dorsal fin origin opposite or anterior to pelvic fin origin 20

19b. (16b) Dorsal fin origin posterior to pelvic fin origin 23

20a. (19a) Lateral line scales markedly elevated (taller than wide) anteriorly, elevated scale height 2 to 5 times scale width *Notropis buchanani*

20b. (19a) Lateral line scales not markedly elevated anteriorly, scale height 1 to 2 times scale width 21

21a. (20b) Black mid-lateral stripe; melanophores form double dashes above and below the lateral line inferior to mid-lateral stripe between pectoral and pelvic fins *Notropis braytoni*

21b. (20b) Faint or absent mid-lateral stripe 22

22a. (21b) Melanophores form double dashes above and below the entire lateral line; without scattered, black melanophores on dorsal of body *Notropis stramineus*

22b. (21b) If present, melanophores form double dashes above and below the anterior portion of the lateral line; with scattered, black melanophores on dorsal of body *Notropis chihuahua*

23a. (19b) Sub-terminal mouth *Notropis simus*

23b. (19b) Terminal mouth 24

24a. (23b) Depressed pelvic fins reach or extend past origin of anal fin; eye larger, eye diameter

greater than snout length *Notropis amabilis*

24b. (23b) Depressed pelvic fins do not reach origin of anal fin; eye smaller, eye diameter about equal to snout length *Notropis jemezanus*

FAMILY CATOSTOMIDAE—suckers

1a. Dorsal fin long, base > than ⅓ of standard length; 22 to 30 dorsal fin rays 2

1b. Dorsal fin short, base < than ¼ of standard length; 4 to 18 dorsal fin rays 5

2a. (1a) Small scales, lateral line scales > 50; eye closer to back of head than to tip of snout;

head abruptly more slender than body; papillose lips *Cycleptus elongatus*

2b. (1a) Large scales, lateral line scales < 45; eye closer to tip of snout than back of head; plicate lips 3

3a. (2b) Subopercle triangular, broadest toward base; knob present at tip of lower lip; blunt snout, forming level with eye *Carpiodes carpio*

3b. (2b) Subopercle semicircular, broadest towards middle; knob absent at tip of lower lip; rounded snout, forming below level of eye 4

4a. (3b) Body elongate and slender, greatest body depth goes 2.6 to 3.3 times in standard length, and height of anterior rays in dorsal and anal fins often less than 2/3 head length in individuals >300 mm; small eye, eye diameter goes ≥ 2 times in snout length of individuals <300 mm *Ictiobus niger*

4b. (3b) Body deep and narrow, greatest body depth goes 2.2 to 2.8 times in standard length, and height of anterior dorsal and anal fin rays often greater than 2/3 head length in individuals >300 mm; large eye, eye diameter goes ≤ 2 times in snout length of individuals <300 mm *Ictiobus bubalus*

5a. (1b) 44 to 46 scales along the lateral line; pectoral fin length equal to head length *Moxostoma congestum*

5b. (1b) 47 to 50 scales along the lateral line; pectoral fin length < head length *Moxostoma austrinum*

FAMILY CHARACIDAE—characins

*Astyanax mexicanus*

FAMILY ICTALURIDAE—bullhead catfishes

1a. Adipose fin joined to the caudal fin or separated by a shallow notch *Noturus gyrinus*

1b. Adipose fin free at tip, not joined to caudal fin 2

2a. (1b) Head dorso-ventrally compressed; mouth terminal to superior *Pylodictis olivaris*

2b. (1b) Head rounded; mouth subterminal 3

3a. (2b) Caudal fin rounded or shallowly emarginate 4

3b. (2b) Caudal fin deeply forked 5

4a. (3a) Chin barbels completely or partially black; anal fin rays 17 to 24; anal fin broadly rounded *Ameiurus melas*

4b. (3a) Chin barbels white or yellow; anal fin rays 24 to 27; margin of anal fin generally straight *Ameiurus natalis*

5a. (3b) Anal fin rays 30 to 36; anal fin free margin is straight; medial keel-like ridge anterior to dorsal fin forms humped back appearance *Ictalurus furcatus*

5b. (3b) Anal fin rays 22 to 29; anal fin free margin is rounded; no humped back appearance 6

6a. (5b) Anal fin rays 27 to 29; pectoral fin spine goes < 5 times into standard length; random scattering of few black spots may be present *Ictalurus punctatus*

6b. (5b) Anal fin rays 22 to 26; pectoral fin spine goes > 5 times into standard length; diffuse black spots on sides *Ictalurus lupus*

FAMILY LORICARIIDAE—suckermouth catfishes

1a. Dorsal fin short with ≤ 9 rays *Hypostomus plecostomus*

1b. Dorsal fin long with ≥ 10 rays; dark spots forming extensive vermiculations on sides and ventral surface *Pterygoplichthys disjunctivus*

FAMILY SALMONIDAE—salmons

1a. Lateral scale rows 120 to 140; basibranchial teeth absent; paired fins with a white border; no deep red to orange slash on each side of throat along inner side of dentary bone; small spots heavily scattered along sides and caudal fin *Oncorhynchus mykiss*

1b. Lateral scale rows 150 to 180; basibranchial teeth usually present, but small or vestigial; paired fins uniformly brown or reddish but without a white border; deep red to orange slash on each side of throat along inner side of dentary bone; large spots concentrated on caudal peduncle in adults *Oncorhynchus clarki*

FAMILY MUGILIDAE—mullets

1a. Lower jaw rounded, without a symphyseal knob; lower limb of 1st gill arch with 17 to 20 gill rakers; no adipose eyelid; scales ctenoid *Agonostomus monticola*

1b. Lower jaw angular, with a prominent symphyseal knob; lower limb of 1st gill arch with 25

to 60 gill rakers; adipose eyelid well developed in adults; scales cycloid in young, ctenoid in adults *Mugil cephalus*

FAMILY ATHERINOPSIDAE—New World silversides

1a. Scales ctenoid, rough to the touch; double pairs of black spots on dorsum; bases of dorsal

and anal fin covered with scales *Membras martinica*

1b. Scales cycloid, smooth to the touch; dorsum with crosshatching, but not double pairs of black spots; bases of dorsal and anal fins not covered with scales; horizontal distance between spinous dorsal and anal fin origin less than 7% of standard length *Menidia audens*

FAMILY FUNDULIDAE—topminnows

1a. Distance from origin of dorsal fin to end of hypural plate < distance from origin of dorsal

fin to preopercle or occasionally about equal to that distance; more than 30 longitudinal scale

rows 2

1b. Distance from origin of dorsal fin to end of hypural plate > distance from origin of dorsal fin to preopercle; 30 or fewer longitudinal scale rows *Lucania parva*

2a. (1a) More than 40 longitudinal scale rows; dark vertical barring; gill slit not extending dorsal to uppermost pectoral fin ray *Fundulus zebrinus*

2b. (1a) Fewer than 40 longitudinal scale rows; gill slit extending dorsal to uppermost pectoral fin ray *Fundulus grandis*

FAMILY CYPRINODONTIDAE— pupfishes

1a. Abdomen without scales anterior to pelvic fins *Cyprinodon pecosensis*

1b. Abdomen with scales anterior to pelvic fins 2

2a. (1b) Six to 7 anal fin rays 3

2b. (1b) Greater than 8 anal fin rays 4

3a. (2a) Dark blotches form a lateral stripe; found in small springs near Balmorhea, TX *Cyprinodon elegans*

3b. (2a) Faint blotches deeper than wide; found in Devils River and Alamito Creek *Cyprinidon eximius*

4a. (2b) Five to 8 triangular dark bars wide dorsally, coming to a point ventrally *Cyprinodon variegatus*

4b. (2b) Lateral blotches wider than deep; found only in Leon Creek. *Cyprinodon bovinus*

FAMILY POECILIIDAE—livebearers

1a. Origin of dorsal fin anterior to anal fin origin; intestinal canal long with many convolutions 2

1b. Origin of dorsal fin posterior to anal fin origin; intestinal canal short with few convolutions 3

2a. (1a) Dorsal fin rays 12 to 14; dorsal fin base more than ½ predorsal length; rows of dark spots on scales obscure diamond-shaped color pattern *Poecilia latipinna*

2b. (1a) Dorsal fin rays 10 to 12; dorsal fin base < ½ predorsal length; dark spots on scales do not obscure diamond-shaped color pattern; only exists as females *Poecilia formosa*

3a. (1b) Spines at tip of 3rd anal fin ray of male gonopodium (first enlarged ray) 1 to 3 times longer than wide 4

3b. (1b) Spines at tip of 3rd anal fin ray of male gonopodium 4 to 10 times longer than wide 5

4a. (3a) Dorsal fin rays 6 (rarely 7); distal end of the 4th fin ray of gonopodium in male parallel or curved in only a weak arch *Gambusia affinis*

4b. (3a) Dorsal fin rays 7; distal end of the 4th fin ray of gonopodium in male curved in a wide arch; found in Devil’s River *Gambusia speciosa*

5a. (3b) Dorsal fin rays 9 (rarely 10); predorsal stripe distinct and broad; found in San Felipe Springs *Gambusia krumholzi*

5b. (3b) Dorsal fin rays 7 to 8; predorsal stripe thin or absent 6

6a. (5b) Lateral stripe broad; caudal fin without prominent black markings; markings on sides crescentric; tip of anterior branch of 4th ray of male gonopodium does not extend to tip of posterior branch 7

6b. (5b) Lateral stripe thin and threadlike; caudal fin with prominent black markings; markings on sides rounded specks; tip of anterior branch of 4th ray of male gonopodium extends as far as tip of posterior branch 8

7a. (6a) Elbow of gonopodium composed of usually 4 fused segments; no dark markings around anus of mature females. Likely extirpated *Gambusia senilis*

7b. (6a) Elbow of gonopodium composed of usually 2 (rarely 3) fused segments; dark markings on anus of mature females *Gambusia gaigei*

8a. (7b) Postanal streak prominent (darker than markings on scale pockets); black markings on mouth; median row of spots on caudal fin; median row of spots on dorsal fin; terminal hook on

4th and 5th rays of gonopodium angular at tip *Gambusia geiseri*

8b. (7b) Postanal streak weaker than markings on scale pockets; dusky or no markings on mouth; no prominent spots in middle of caudal fin; a subbasal row of spots on dorsal fin; terminal hooks on 4th and 5th rays of gonopodium rounded at tip *Gambusia nobilis*

FAMILY MORONIDAE—temperate basses

1a. Body depth goes < 3 times in standard length; teeth in single patch on back of tongue *Morone chrysops*

1b. Body depth goes > 3 times in standard length; teeth in 2 parallel patches on back of tongue *Morone saxatilis*

FAMILY CENTRARCHIDAE—sunfishes

1a. Five to 8 anal spines 2

1b. Three anal spines 3

2a. (1a) Dorsal fin set back on body, length of dorsal fin base < distance from its origin to posterior margin of eye; lateral body with wide to narrow dorsal black bands; ≤ 6 dorsal spines *Pomoxis annularis*

2b. (1a) Dorsal fin set forward on body, length of dorsal fin base equal to or greater than distance from its origin to posterior margin of eye; lateral body with checkerboard black and light pattern; ≥7 dorsal spines *Pomoxis nigromaculatus*

3a. (1b) Body slender, body depth contained > 3 times into standard length 4

3b. (1b) Body deep, body depth contained < 3 times into standard length 5

4a. (3a) Dorsal fins narrowly joined at base forming a deep notch; upper jaw extends past posterior margin of eye in adults; mid-lateral stripe generally complete, rows of spots ventral to mid-lateral stripe faint and incomplete *Micropterus salmoides*

4b. (3a) Dorsal fins broadly joined at base forming a shallow notch; upper jaw does not reach past posterior portion of eye; bases of soft dorsal and anal fins scaled *Micropterus dolomieu*

5a. (3b) Teeth on tongue; head and opercle with 3 to 5 distinct dark and light longitudinal stripes; red spot on posterior margin of opercle flap in fresh specimens *Lepomis gulosus*

5b. (3b) No teeth on tongue; head and opercle lacking distinct dark and light longitudinal stripes 6

6a. (5b) Pectoral fins long and pointed, reach anterior portion of eye or beyond when bent forward 7

6b. (5b) Pectoral fins short and rounded, do not reach past eye when bent forward 8

7a. (6a) Opercle flap stiff to its margin, posterior margin either red or orange in live specimens *Lepomis microlophus*

7b. (6a) Opercle flap flexible, posterior margin not red or orange in live specimens *Lepomis macrochirus*

8a. (6b) Black opercle flap stiff near the posterior margin with bone supporting all or majority of the flap 9

8b. (6b) Black opercle flap flexible near the posterior margin without bone supporting majority of the flap 10

9a. (8a) Body elongated with black spot on posterior base of soft dorsal fin *Lepomis cyanellus*

9b. (8a) Body rounded without black spot on posterior base of soft dorsal fin; lateral body with alternating stripes formed from black and red spots *Lepomis miniatus*

10a. (8b) Opercle flap black to the posterior margin; opercle flap is thin near the opercle bone with the narrowest width of the flexible portion of the flap about the same diameter of the eye pupil *Lepomis auritus*

10b. (8b) Opercle flap black and surrounded by white on the posterior margin; opercle flap is wide with narrowest width of the flexible flap is about two times the diameter of the eye pupil *Lepomis megalotis*

FAMILY PERCIDAE—perches

1a. Upper jaw extending to below the middle of the eye or farther; preopercle strongly serrate; caudal fin forked *Sander vitreus*

1b. Upper jaw not extending to beneth middle of eye; preopercle smooth or weakly serrated 2

2a. (1b) Snout conical, extends beyond upper lip; body with thin vertical bars; caudal fin straight, rounded, or slightly emarginate *Percina macrolepida*

2b. (1b) Snout less conical, does not extend beyond upper lip 2

3a. (2b) Lateral line arched upward *Etheostoma gracile*

3b. (2b) Lateral line straight *Etheostoma grahami*

FAMILY SCIAENIDAE—drums

*Aplodinotus grunniens*

FAMILY CICHLIDAE—cichlids

1a. Anal fin spines 5 to 6 *Herichthys cyanoguttatus*

1b. Anal fin spines < 5 (usually 3) 2

2a. (1b) Gill rakers 14 to 20 (usually 17 to 18) on lower part of first gill arch; most teeth in outer row are unicuspid in adults; sides with 3 or 4 dark blotches or with no markings; no yellow on dorsal fin; caudal fin without distinct vertical stripes *Oreochromis mossambicus*

2b. (1b) Gill rakers 18 to 26 on lower part of 1st gill arch; outer row of teeth bicuspid in adults; caudal fin unmarked, or with vague, irregular dark markings, caudal fin often with a broad, red distal margin; young often with vertical bands on caudal fin *Oreochromis aureus*

FAMILY GOBIIDAE—gobies

1a. Body without scales *Gobiosoma bosc*

1b. Body mostly scaled; Scales small, > 70 rows in lateral series *Awaous banana*

**SABINE AND NECHES RIVER BASINS**

KEY TO THE FAMILIES

1a. Jawless, disc-shaped mouth; without pectoral and pelvic fins; 7 pairs of external gill openings Lampreys – Petromyzontidae

1b. Jawed mouth; one gill opening on each side of head; with pectoral, pelvic, or both fins 2

2a. (1b) Both eyes on one side of head; without right pectoral fin American Soles – Achiridae

2b. (1b) One eye on either side of head; with both pectoral fins 3

3a. (2b) Body long and slender; without pelvic fins Freshwater Eels – Anguillidae

3b. (2b) Body truncated or elongated; with pelvic fin 4

4a. (3b) Caudal fin heterocercal or abbreviated heterocercal 5

4b. (3b) Caudal fin homocercal 7

5a. (4a) Caudal fin heterocercal, body appears scaleless, except for a few ganoid scales at the base of caudal fin; long, paddle shaped snout Paddlefish – Polyodontidae

5b. (4a) Caudal fin abbreviated heterocercal; body with ganoid or cycloid scales 6

6a. (5b) Body covered with ganoid scales; snout formed into a beak; without gular plate Gars – Lepisosteidae

6b. (5b) Body covered with cycloid scales, snout not formed into a beak, with a gular plate Bowfin – Amiidae

7a. (4b) Jaws duckbilled Pikerels – Esocidae

7b. (4b) Jaws not duckbilled 8

8a. (7b) One dorsal fin; pelvic fins without uniserial spines 9

8b. (7b) One or two dorsal fins; pelvic fins with uniserial spines 15

9a. (8a) With adipose fin 10

9b. (8a) Without adipose fin 11

10a. (9a) Without barbels Trouts – Salmonidae

10b. (9a) With barbels Bullhead Catfishes – Ictaluridae

11a. (9b) Long anal fin with ≥ 17 fin rays Shads – Clupiedae

11b. (9b) Short anal fin with ≤ 13 rays 12

12a. (11b) Caudal fin forked or emarginated; lateral line usually present 13

12b. (11b) Caudal fin truncated or rounded; lateral line usually absent 14

13a. (12a) Inferior, fleshy mouth modified for sucking; > 7 pharyngeal teeth in main row, usually ≥ 10 dorsal fin rays Suckers – Catostomidae

13b. (12a) Mouth usually not fleshy or modified for sucking; < 7 pharyngeal teeth in main row, usually ≤ 10 dorsal fin rays Minnows – Cyprinidae

14a. (12b) Mature males with rounded anal fin; males and females with 3rd anal fin ray branched, no gonopodium present Killifishes – Fundulidae

14b. (12b) Mature males with pointed anal fin forming a gonopodium; males and females with 3rd anal fin ray unbranched. Livebearers – Poeciliidae

15a. (8b) Anus anterior to pelvic fins; > 5 soft rays on each pelvic fin Pirate Perch – Aphredoderidae

15b. (8b) Anus posterior to pelvic fins; 5 soft rays on pelvic fins 16

16a. (15b) Pelvic fin position abdominal or sub-thoracic; dorsal fins widely separated 17

16b. (15b) Pelvic fin position thoracic; dorsal fins joined or, if separate, closely adjacent to one another 18

17a. (16a) Dorsal fin with 4 thick spines; anal fin with 2-3 spines; adipose eyelids present Mullets – Mugilidae

17b. (16a) Dorsal fin with 4 to 8 thin spines; anal fin with 1 spine; adipose eyelids absent Silversides – Atherinopsinidae

18a. (16b) One nostril (nare) on each side of head; lateral line interrupted Cichlids – Cichlidae

18b. (16b) Two nostrils (nares) on each side of head; lateral line complete, incomplete, or absent 19

19a. (17b) Dorsal fin with > 23 fin rays; lateral line extends to tip of caudal fin Drums – Sciaenidae

19b. (17b) Dorsal fin with < 23 fin rays; lateral line, if present, does not extend to tip of caudal fin 20

20a. (19b) Anal fin with 1 to 2 spines Perches – Percidae

20b. (19b) Anal fin with 3 to 8 spines 21

21a. (20b) Posterior margin of operculum with a sharp spine; spiny and soft dorsal fin separate or only slightly connected; pseudobranchium present and exposed Temparate Basses – Moronidae

21b. (20b) Posterior margin of operculum without a sharp spine; spiny and soft dorsal fins connected or with deep notch; pseudobranchium covered or absent 22

22a. (21b) Lateral line present or incomplete Sunfishes – Centrarchidae

22b. (21b) Lateral line absent Pygmy Sunfishes – Elassomatidae

KEY TO THE SPECIES

FAMILY PETROMYZONTIDAE – lampreys

1a. Disc-shaped mouth large, diameter of mouth > body width and about 140 times into total length; with rasping teeth; adults with well-developed intestine *Ichthyomyzon castaneus*

1b. Disc-shaped mouth small, diameter of mouth ≤ than body width and about 170 to 250 times into total length; without rasping teeth; adults without well-developed intestine *Ichthyomyzon gagei*

FAMILY POLYODONTIDAE—paddlefishes

*Polyodon spathula*

FAMILY LEPISOSTEIDAE—gars

1a. Large teeth in upper jaw in parallel rows on each side *Atractosteus spatula*

1b. Large teeth in upper jaw one row, although another non-parallel row might be present 2

2a. (1b) Beak long and narrow, least width goes about 12 to 20 times in length; width of beak at nostrils < eye diameter; snout > ⅔ of head length *Lepisosteus osseus*

2b. (1b) Beak short and blunt, least width goes about 5 to 7 times in length; width of beak at nostrils > eye diameter; snout < ⅔ of head length *Lepisosteus oculatus*

FAMILY AMIIDAE—bowfins

*Amia calva*

FAMILY ANGUILLIDAE—freshwater eels

*Anguilla rostrata*

FAMILY CLUPEIDAE—herrings

1a. Twenty-nine to 33 anal fin rays; mouth subterminal and below level of middle of eye; black

shoulder spot ≥ pupil of eye *Dorosoma cepedianum*

1b. Twenty-four to 28 anal fin rays; mouth terminal and at level of eye; black shoulder spot < pupil of eye *Dorosoma petenense*

FAMILY CYPRINIDAE - minnows

1a. More than 15 soft rays on dorsal fin; dorsal and anal fins each with a strong serrated spine 2

1b. Fewer than 10 soft rays on dorsal fin; dorsal and anal fins without spine 3

2a. (1a) Upper jaw with two pairs of barbels *Cyprinus carpio*

2b. (1a) Upper jaw without barbels *Carassius auratus*

3a. (1b) fin near caudal fin, distance from snout to origin of anal fin is > 2.5 times the distance from origin of anal fin to base of caudal fin; pharyngeal teeth with prominent parallel grooves *Ctenopharyngodon idella*

3b. (1b) Anal fin not noticeably near caudal fin: distance from snout to origin of anal fin is < 2.5 times the distance from origin of anal fin to base of caudal fin; pharyngeal teeth without prominent parallel grooves 4

4a. (3b) Abdomen behind pelvic fins with a fleshy keel lacking scales; lateral line greatly decurved, distance between anterior lateral line scale and ventral most lateral line scale is > 3 scales in height *Notemigonus crysoleucas*

4b. (3b) Abdomen behind pelvic fin with scales; lateral line not greatly decurved, lateral line descends < 3 scales ventrally from highest point 5

5a. (4b) With maxillary barbels, might be small and not observable without opening the mouth or with magnification 6

5b. (4b) Without maxillary barbels 7

6a. (5a) Mouth terminal; distinct black spot located anteriorly on dorsal fin; pharyngeal teeth on main row 5-4 or 5-5 *Semotilus atromaculatus*

6b. (5a) Mouth subterminal or inferior; no distinct black spot on dorsal fin; pharyngeal teeth on main row 4-4 *Macrhybopsis hyostoma*

7a. (5b) Thick lower lip at corners, mouth noticeably ventral; black spot at base of caudal fin *Phenacobius mirabilis*

7b. (5b) Lower lip thin or not noticeably thick; with or without black spot at base of caudal fin 8

8a. (7b) Predorsal scales appear crowded, smaller than scales on lateral body or appear as

overlapping scales; black spot in the middle, anterior portion of the dorsal fin 9

8b. (7b) Predorsal scales not crowded; without black spot in the middle, anterior portion of

the dorsal fin 10

9a. (8a) Caudal spot, if distinct, continuous with mid-lateral stripe; lateral line incomplete; intestine long, more than twice the standard length. *Pimephales promelas*

9b. (8a) Caudal spot distinct from mid-lateral stripe; lateral line complete, intestine forming a short S-shaped loop *Pimephales vigilax*

10a. (8b) Long intestine in a flat coil 11

10b. (8b) Short S-shaped intestine 12

11a. (10a) Terminal mouth; scales are diamond-shaped, most noticeable dorsally; Sabine drainage *Hybognathus hayi*

11b. (10a) Sub-terminal mouth; scales not diamond-shaped *Hybognathus nuchalis*

12a. (10b) Moderately decurved lateral line; diamond-shaped scales; dark shoulder patch present; melanophores concentrated between rays of dorsal and anal fins 13

12b. (10b) Lateral line incomplete, complete-straight, or complete-slightly decurved; scales not noticeably diamond-shaped; without dark shoulder patch; melanophores concentrated along rays of dorsal and anal fins 14

13a. (12a) Sub-terminal mouth; caudal fin base with a caudal spot larger than eye; no shoulder patch *Cyprinella venusta*

13b. (12a) Terminal mouth; no caudal spot *Cyprinella lutrensis*

14a. (12b) Distinct and separate black dash at base of dorsal fin; apparent when viewed from above; pharyngeal teeth count usually 0,4-4,0 15

14b. (12b) No distinct and separate black dash at base of dorsal fin; pharyngeal teeth count usually 1,4-4,1 or 2,4-4,2 17

15a. (14a) Lateral line scales markedly elevated (taller than wide) anteriorly, elevated scale height 2 to 5 times scale width 16

15b. (14a) Lateral line scales not markedly elevated anteriorly, scale height 1 to 2 times scale width *Notropis sabinae*

16a. (15a) Dorsal and lateral body with melanophores outlining scales; with pronounced black lateral stripe; dorsal fin height goes 2.1 or more times in pre-dorsal length; infraorbital canal complete *Notropis volucellus*

16b. (15a) Dorsal and lateral body with sparse melanophores; scales outlined with melanophores are rare; pre-dorsal black spot is prominent and distinct from mid-dorsal stripe; dorsal fin height goes 2.0 or fewer times in pre-dorsal length; infraorbital canal incomplete *Notropis buchanani*

17a. (14b) Depressed dorsal fin longer than head. *Hybopsis amnis*

17b. (14b) Depressed dorsal fin shorter than head 19

18a. (17b) Mouth is sub-terminal; pharyngeal teeth are 0,4-4,0 *Notropis atrocaudalis*

18b. (17b) Mouth is terminal; pharyngeal teeth are 1,4-4,1 or 2,4-4,2 or 5-5 19

19a. (18b) Dorsal fin origin opposite or anterior to pelvic fin origin 20

19b. (18b) Dorsal fin origin posterior to pelvic fin origin 23

20a. (19a) Prominent mid-lateral stripe, extending through eye 21

20b. (19a) No prominent mid-lateral stripe present *Notropis shumardi*

21a. (20a) Pharyngeal teeth 5-5; mouth small and almost vertical *Opsopoeodus emiliae*

21b. (20a) Pharyngeal teeth are 1,4-4,1 or 2,4-4,2; mouth large and oblique 22

22a. (21b) Usually 8 anal fin rays; inside of mouth with black melanophores; dorsal fin insertion is opposite to pelvic fin insertion *Notropis chalybaeus*

22b. (21b) Usually 7 anal fin rays; inside of mouth without black melanophores; dorsal fin insertion is anterior to pelvic fin insertion *Notropis texanus*

23a. (19b) Small scales, ≥ 41 lateral line scales, > 25 predorsal scales *Notropis atherinoides*

23b. (19b) Moderate-sized scales, ≤ 40 lateral line scales, < 24 predorsal scales 24

24a. (23b) Dorsal fin with black melanophores extending from mid-dorsal stripe into the anterior fin ray; edges of the anterior dorsolateral scales are outlined with black melanophores, producing

chevron pattern. *Lythrurus umbratilis*

24b. (23b) Dorsal fin with black melanophores not extending from mid-dorsal stripe into the anterior fin ray, lacks chevron pattern *Lythrurus fumeus*

FAMILY CATOSTOMIDAE—suckers

1a. Dorsal fin long, base > than ⅓ of standard length; 22 to 30 dorsal fin rays 2

1b. Dorsal fin short, base < than ¼ of standard length; 4 to 18 dorsal fin rays 6

2a. (1a) Small scales, lateral line scales > 50; eye closer to back of head than to tip of snout;

head abruptly more slender than body; papillose lips *Cycleptus elongatus*

2b. (1a) Large scales, lateral line scales < 45; eye closer to tip of snout than back of head; plicate lips 3

3a. (2b) Subopercle triangular, broadest toward base; knob present at tip of lower lip; blunt snout, forming level with eye *Carpiodes carpio*

3b. (2b) Subopercle semicircular, broadest towards middle; knob absent at tip of lower lip; rounded snout, forming below level of eye 4

4a. (3b) Mouth large and oblique; upper jaw length is equal to snout length *Ictiobus cyprinellus*

4b. (3b) Mouth small and nearly horizontal; upper jaw shorter than snout 5

5a. (4b) Body elongate and slender, greatest body depth goes 2.6 to 3.3 times in standard length, and height of anterior rays in dorsal and anal fins often less than 2/3 head length in individuals >300 mm; small eye, eye diameter goes ≥ 2 times in snout length of individuals <300 mm *Ictiobus niger*

5b. (4b) Body deep and narrow, greatest body depth goes 2.2 to 2.8 times in standard length, and height of anterior dorsal and anal fin rays often greater than 2/3 head length in individuals >300 mm; large eye, eye diameter goes ≤ 2 times in snout length of individuals <300 mm *Ictiobus bubalus*

6a. (1b) Lateral line complete and well developed; air bladder with 3 chambers *Moxostoma poecilurum*

6b. (1b) Lateral line incomplete or absent; air bladder with 2 chambers 7

7a. (6b) Lateral line incomplete; rows of spots *Minytrema melanops*

7b. (6b) Lateral line absent 8

8a. (7b) Scales larger, lateral scale count 34 to 37; eye larger, eye length ½ of snout length; dorsal fin rays 11 or 12; back with crescentic scale marks *Erimyzon sucetta*

8b. (7b) Scales smaller, lateral scale count 39 to 43; eye smaller, eye length < ½ of snout length); dorsal fin rays 9 or 10; back without crescentic scale marks *Erimyzon claviformis*

FAMILY ICTALURIDAE—bullhead catfishes

1a. Adipose fin joined to the caudal fin or separated by a shallow notch 2

1b. Adipose fin free at tip, not joined to caudal fin 3

2a. (1a) Mouth terminal; pectoral fin spine not serrated; lower lip and chin not heavily speckled with black pigment *Noturus gyrinus*

2b. (1a) Mouth sub-terminal; pectoral spine serrated; lower lip and chin heavily speckled with black pigment. *Noturus nocturnus*

3a. (1b) Head dorso-ventrally compressed; mouth terminal to superior *Pylodictis olivaris*

3b. (1b) Head rounded; mouth subterminal 4

4a. (3b) Caudal fin rounded or shallowly emarginate 5

4b. (3b) Caudal fin deeply forked 6

5a. (4a) Chin barbels completely or partially black; anal fin rays 17 to 24; anal fin broadly rounded *Ameiurus melas*

5b. (4a) Chin barbels white or yellow; anal fin rays 24 to 27; margin of anal fin generally straight

*Ameiurus natalis*

6a. (4b) Anal fin rays 30 to 36; anal fin free margin is straight; medial keel-like ridge anterior to dorsal fin forms humped back appearance *Ictalurus furcatus*

6b. (4b) Anal fin rays 22 to 29; anal fin free margin is rounded; no humped back appearance *Ictalurus punctatus*

FAMILY SALMONIDAE—salmons

*Oncorhynchus mykiss*

FAMILY ESOCIDAE—pikes and pickerels

1a. Snout short, distance from tip of snout to center of eye ≤ distance from center of eye to rear margin of operculum; < 115 scale rows along body *Esox americanus*

1b. Snout long, distance from tip of snout to center of eye > distance from center of eye to rear

margin of operculum; > 120 scale rows along body *Esox niger*

FAMILY APHREDODERIDAE—pirate perch

*Aphredoderus sayanus*

FAMILY MUGILIDAE—mullets

1a. Lower jaw rounded, without a symphyseal knob; lower limb of 1st gill arch with 17 to 20 gill rakers; no adipose eyelid; scales ctenoid *Agonostomus monticola*

1b. Lower jaw angular, with a prominent symphyseal knob; lower limb of 1st gill arch with 25

to 60 gill rakers; adipose eyelid well developed in adults; scales cycloid in young, ctenoid in adults *Mugil cephalus*

FAMILY ATHERINOPSIDAE—New World silversides

1a. Scales small, > 60 scales in lateral series, jaws produced into a short beak; snout length > eye length; > 20 anal fin rays *Labidesthes sicculus*

1b. Scales large, < 50 scales in lateral series; jaws not produced into a beak; snout length ≤ eye length; < 20 anal fin rays *Menidia audens*

FAMILY FUNDULIDAE—topminnows

1a. Body with a distinct black lateral band 2

1b. Body without a distinct black lateral band 3

2a. (1a) Distinct black spots on anterior dorso-lateral region are as pronounced as lateral stripe; distinct black spots throughout dorsal and caudal fins *Fundulus olivaceus*

2b. (1a) Faint black spots on anterior dorso-lateral region are not as pronounced as lateral stripe; distinct black spots near base of dorsal and caudal fins *Fundulus notatus*

3a. (1b) Red to dark spots in multiple rows longitudinally along lateral sides; usually with dark subocular bar *Fundulus blairae*

3b. (1b) Body mottled, barred or irregularly spotted; no dark subocular bar *Fundulus chrysotus*

FAMILY POECILIIDAE—livebearers

1a. Origin of dorsal fin anterior to anal fin origin; intestinal canal long with many convolutions *Poecilia latipinna*

1b. Origin of dorsal fin posterior to anal fin origin; intestinal canal short with few convolutions

2

2a. (1b) Dorsal fin origin slightly behind anal fin origin; dark band on sides with vertical bars; large black spots near bases of dorsal and caudal fins of both sexes and on anal fin of females *Heterandria formosa*

2b. (1b) Dorsal fin origin well behind anal fin origin; no dark band on sides; median fins without

large black spots near their bases *Gambusia affinis*

FAMILY MORONIDAE—temperate basses

1a. Dorsal fins united at base; 2nd and 3rd anal fin spines approximately equal in length; no teeth on tongue; 9 to 10 anal fin soft rays; stripes along sides usually sharply broken and offset above front of anal fin *Morone mississippiensis*

1b. Dorsal fins separated; 2nd anal fin spine much shorter than 3rd; base of tongue with teeth;

11 to 13 anal fin soft rays; stripes along sides usually continuous 2

2a. (1b) Body depth goes < 3 times in standard length; teeth in single patch on back of

tongue *Morone chrysops*

2b. (1b) Body depth goes > 3 times in standard length; teeth in 2 parallel patches on back

of tongue *Morone saxatilis*

FAMILY CENTRARCHIDAE—sunfishes

1a. Five to 8 anal spines 2

1b. Three anal spines 4

2a. (1a) Eleven to 13 dorsal fin spines *Centrarchus macropterus*

2b. (1a) Six to 8 dorsal fin spines 3

3a. (2b) Dorsal fin set back on body, length of dorsal fin base < distance from its origin to posterior margin of eye; lateral body with wide to narrow dorsal black bands; ≤ 6 dorsal spines *Pomoxis annularis*

3b. (2b) Dorsal fin set forward on body, length of dorsal fin base equal to or greater than distance from its origin to posterior margin of eye; lateral body with checkerboard black and light pattern; ≥7 dorsal spines *Pomoxis nigromaculatus*

4a. (1b) Body slender, body depth contained > 3 times into standard length 5

4b. (1b) Body deep, body depth contained < 3 times into standard length 6

5a. (4a) Dorsal fins narrowly joined at base forming a deep notch; upper jaw extends past posterior margin of eye in adults; mid-lateral stripe generally complete, rows of spots ventral to mid-lateral stripe faint and incomplete *Micropterus salmoides*

5b. (4a) Dorsal fins broadly joined at base forming a shallow notch; upper jaw does not reach past posterior portion of eye; bases of soft dorsal and anal fins scaled *Micropterus punctulatus*

6a. (4b) Teeth on tongue; head and opercle with 3 to 5 distinct dark and light longitudinal stripes; red spot on posterior margin of opercle flap in fresh specimens *Lepomis gulosus*

6b. (4b) No teeth on tongue; head and opercle lacking distinct dark and light longitudinal stripes 7

7a. (6b) Pectoral fins long and pointed, reach anterior portion of eye or beyond when bent forward 8

7b. (6b) Pectoral fins short and rounded, do not reach past eye when bent forward 10

8a. (7a) Opercle flap stiff to its margin, posterior margin either red or orange in live specimens *Lepomis microlophus*

8b. (7a) Opercle flap flexible, posterior margin not red or orange in live specimens 9

9a. (8b) Opercle flap black to the margin; black spot on posterior base of soft dorsal fin *Lepomis macrochirus*

9b. (8b) Opercle flap outlined with thick white band; lacking black spot on posterior base of soft dorsal fin *Lepomis humilis*

10a. (7b) Black opercle flap stiff near the posterior margin with bone supporting all or majority of the flap 11

10b. (7b) Black opercle flap flexible near the posterior margin without bone supporting majority of the flap 13

11a. (10a) Lateral line incomplete; smaller individuals with black spot surrounded by white margin on posterior base of soft dorsal fin *Lepomis symmetricus*

11b. (10a) Lateral line complete; black spot, if present, on posterior base of soft dorsal fin without white margin 12

12a. (11b) Body elongated with black spot on posterior base of soft dorsal fin *Lepomis cyanellus*

12b. (11b) Body rounded without black spot on posterior base of soft dorsal fin; lateral body with alternating stripes formed from black and red spots *Lepomis miniatus*

13a. (10b) Opercle flap black to the posterior margin; opercle flap is thin near the opercle bone with the narrowest width of the flexible portion of the flap about the same diameter of the eye pupil *Lepomis auritus*

13b. (10b) Opercle flap black and surrounded by white on the posterior margin; opercle flap is wide with narrowest width of the flexible flap is about two times the diameter of the eye pupil 14

14a. (13b) Twelve pectoral fin rays, 3 to 5 cheek scales; opercle flap often with white pigment form speckles, distinct red spots (white in preserved specimens) along lateral line *Lepomis marginatus*

14b. (13b) Thirteen to 15 pectoral fin rays, 5 to 7 cheek scales; opercle flaps with red or white margin; 13 to 15 pectoral fin rays *Lepomis megalotis*

FAMILY PERCIDAE—perches

1a. Body depth contained in standard length > 7 times 2

1b. Body depth contained in standard length < 7 times 3

2a. (1a) Lateral blotches longer than deep *Ammocrypta clara*

2b. (1a) Lateral blotches deeper than long *Ammocrypta vivax*

3a. (1b) Snout conical, extends beyond upper lip; body with ≥ 14 black vertical bars *Percina macrolepida*

3b. (1b) Snout less conical, does not extend beyond upper lip; body with < 14 black vertical bars or with a pattern other than vertical bars 4

4a. (3b) Sides of body with large black blotches; midline of abdomen naked or with enlarged scales 5

4b. (3b) Sides of body without large black blotches; scales on abdomen normal 7

5a. (4a) Upper lip connected to snout by a narrow frenum; blotches on sides of body are rectangle-shaped and bleed downward *Percina shumardi*

5b. (4a) Upper lip connected to snout by a broad frenum; blotches on sides of body do not bleed downward 6

6a. (5b) Nape unscaled; blotches on sides of body are rectangle shaped and might appear connected *Percina maculata*

6b. (5b) Nape scaled; blotches on sides of body not rectangle shaped *Percina sciera*

7a. (4b) Lateral line short, < 6 pored scales; single row of horizontal dashes present *Etheostoma proeliare*

7b. (4b) Lateral line long (complete or incomplete), > 6 pored scales; if horizontal dashes present, accompanied by vertical bars 8

8a. (7b) Pectoral fin long; pectoral fin folded forward extends past head *Etheostoma histrio*

8b. (7b) Pectoral fin short, pectoral fin folded forward does not extend past head 9

9a. (8b) Lateral line arched upward 10

9b. (8b) Lateral line straight 11

10a. (9a) Breast without scales; breast and abdomen without black speckles *Etheostoma gracile*

10b. (9a) Breast with scales; breast and abdomen with black speckles *Etheostoma fusiforme*

11a. (9b) Lateral body with distinct series of M-shaped pigments; snout rounded and blunt *Etheostoma chlorosoma*

11b. (9b) Lateral body without distinct series of M-shaped pigments; snout not noticeably rounded and blunt 12

12a. (11b) Lateral region with mottling bisected by a light colored lateral stripe *Etheostoma parvipinne*

12b. (11b) Lateral region without mottling bisected by a light colored lateral stripe 13

13a. (12b) Gill membranes widely joined across isthmus; lateral region with red or yellow spots *Etheostoma artesiae*

13b. (12b) Gill membranes either not joined or barely joined across isthmus; lateral region without spots and with a series of vertical bars located posteriorly *Etheostoma asprigene*

FAMILY SCIAENIDAE—drums

*Aplodinotus grunniens*

FAMILY ELASSOMATIDAE—pygmy sunfishes

*Elassoma zonatum*

FAMILY ACHIRIDAE—American soles

*Trinectes maculatus*

**TRINITY AND SAN JACINTO RIVER BASINS**

KEY TO THE FAMILIES

1a. Jawless, disc-shaped mouth; without pectoral and pelvic fins; 7 pairs of external gill openings Lampreys – Petromyzontidae

1b. Jawed mouth; one gill opening on each side of head; with pectoral, pelvic, or both fins 2

2a. (1b) Both eyes on one side of head; without right pectoral fin American Soles – Achiridae

2b. (1b) One eye on either side of head; with both pectoral fins 3

3a. (2b) Body long and slender; without pelvic fins Freshwater Eels – Anguillidae

3b. (2b) Body truncated or elongated; with pelvic fins 4

4a. (3b) Caudal fin heterocercal or abbreviated heterocercal 5

4b. (3b) Caudal fin homocercal 7

5a. (4a) Caudal fin heterocercal, body appears scaleless, except for a few ganoid scales at the base of caudal fin; long, paddle shaped snout Paddlefish – Polyodontidae

5b. (4a) Caudal fin abbreviated heterocercal; body with ganoid or cycloid scales 6

6a. (5b) Body covered with ganoid scales; snout formed into a beak; without gular plate Gars – Lepisosteidae

6b. (5b) Body covered with cycloid scales, snout not formed into a beak, with a gular plate Bowfin – Amiidae

7a. (4b) Jaws duckbilled Pikerels – Esocidae

7b. (4b) Jaws not duckbilled 8

8a. (7b) One dorsal fin; pelvic fins without uniserial spines 9

8b. (7b) One or two dorsal fins; pelvic fin with a hard spine 18

9a. (8a) With adipose fin 10

9b. (8a) Without adipose fin 13

10a. (9a) Without barbels 12

10b. (9a) With barbels 11

11a. (10b) Body covered with bony plates; head with one pair of barbels Armored Catfishes – Loricariidae

11b. (10b) Scales absent; head with four to eight barbels Bullhead Catfishes – Ictaluridae

12a. (10a) Scales large, < 50 lateral line scales; incisor teeth present Tetras – Characidae

12b. (10a) Scales small, > 60 lateral line scales; incisor teeth absent Trouts – Salmonidae

13a. (10b) Long anal fin with ≥ 17 fin rays Shads – Clupiedae

13b. (10b) Short anal fin with ≤ 13 rays 14

14a. (12b) Caudal fin forked or emarginated; lateral line usually present 15

14b. (12b) Caudal fin truncated or rounded; without a distinct lateral line in most 16

15a. (14a) Inferior, fleshy mouth modified for sucking; > 7 pharyngeal teeth in main row, usually ≥ 10 dorsal fin rays Suckers – Catostomidae

15b. (14a) Mouth usually not fleshy or modified for sucking; < 7 pharyngeal teeth in main row, usually ≤ 10 dorsal fin rays Minnows – Cyprinidae

16a. (14b) Mature males with rounded anal fin; males and females with 3rd anal fin ray branched, no gonopodium present 17

16b. (14b) Mature males with pointed anal fin forming a gonopodium; males and females with 3rd anal fin ray unbranched. Livebearers – Poeciliidae

17a. (16a) Body robust; teeth in single row are incisor-like and tricuspid (three points on a tooth). Pupfishes – Cyprinodontidae

17b. (16a) Body elongate; conical (cone-shaped) pointed teeth in a single row or several rows Killifishes – Fundulidae

18a. (8b) Anus anterior to pelvic fins; > 5 soft rays on each pelvic fin Pirate Perch – Aphredoderidae

18b. (8b) Anus posterior to pelvic fins; 5 soft rays on pelvic fins 19

19a. (18b) Pelvic fin position abdominal or sub-thoracic; dorsal fins widely separated 20

19b. (18b) Pelvic fin position thoracic; dorsal fins joined or, if separate, closely adjacent to one another 21

20a. (19a) Dorsal fin with 4 thick spines; anal fin with 2-3 spines; adipose eyelids present Mullets – Mugilidae

20b. (19a) Dorsal fin with 4 to 8 thin spines; anal fin with 1 spine; adipose eyelids absent Silversides – Atherinopsidae

21a. (19b) One nostril (nare) on each side of head; lateral line interrupted Cichlids – Cichlidae

21b. (19b) Two nostrils (nares) on each side of head; lateral line complete, incomplete, or absent 22

22a. (21b) Dorsal fin with > 23 fin rays; lateral line extends to tip of caudal fin Drums – Sciaenidae

22b. (21b) Dorsal fin with < 23 fin rays; lateral line, if present, does not extend to tip of caudal fin 23

23a. (22b) Anal fin with 1 to 2 spines Perches – Percidae

23b. (22b) Anal fin with 3 to 8 spines 24

24a. (23b) Posterior margin of operculum with a sharp spine; spiny and soft dorsal fin separate or only slightly connected; pseudobranchium present and exposed Temperate Basses – Moronidae

24b. (23b) Posterior margin of operculum without a sharp spine; spiny and soft dorsal fins connected or with deep notch; pseudobranchium covered or absent 24

25a. (24b) Lateral line present or incomplete Sunfishes – Centrarchidae

25b. (24b) Lateral line absent Pygmy Sunfishes – Elassomatidae

KEY TO THE SPECIES

FAMILY PETROMYZONTIDAE – lampreys

*Ichthyomyzon gagei*

FAMILY POLYODONTIDAE—paddlefishes

*Polyodon spathula*

FAMILY LEPISOSTEIDAE—gars

1a. Large teeth in upper jaw in parallel rows on each side *Atractosteus spatula*

1b. Large teeth in upper jaw one row, although another non-parallel row might be present 2

2a. (1b) Beak long and narrow, least width goes about 12 to 20 times in length; width of beak at nostrils < eye diameter; snout > ⅔ of head length *Lepisosteus osseus*

2b. (1b) Beak short and blunt, least width goes about 5 to 7 times in length; width of beak at nostrils > eye diameter; snout < ⅔ of head length *Lepisosteus oculatus*

FAMILY AMIIDAE—bowfins

*Amia calva*

FAMILY ANGUILLIDAE—freshwater eels

*Anguilla rostrata*

FAMILY CLUPEIDAE—herrings

1a. Twenty-nine to 33 anal fin rays; mouth subterminal and below level of middle of eye; black shoulder spot ≥ pupil of eye *Dorosoma cepedianum*

1b. Twenty-four to 28 anal fin rays; mouth terminal and at level of eye; black shoulder spot < pupil of eye *Dorosoma petenense*

FAMILY CYPRINIDAE - minnows

1a. More than 15 soft rays on dorsal fin; dorsal and anal fins each with a strong serrated spine 2

1b. Fewer than 10 soft rays on dorsal fin; dorsal and anal fins without spine 3

2a. (1a) Upper jaw with two pairs of barbels *Cyprinus carpio*

2b. (1a) Upper jaw without barbels *Carassius auratus*

3a. (1b) Anal fin near caudal fin, distance from snout to origin of anal fin is > 2.5 times the distance from origin of anal fin to base of caudal fin; pharyngeal teeth with prominent parallel grooves *Ctenopharyngodon idella*

3b. (1b) Anal fin not noticeably near caudal fin: distance from snout to origin of anal fin is < 2.5 times the distance from origin of anal fin to base of caudal fin; pharyngeal teeth without prominent parallel grooves 4

4a. (3b) Intestine wound spirally around swim bladder; keratinous ridge on lower jaw *Campostoma anomalum*

4b. (3b) Intestine not wound spirally around swim bladder; keratinous ridge of lower jaw hardly evident 5

5a. (4b) Abdomen behind pelvic fins with a fleshy keel lacking scales; lateral line greatly decurved, distance between anterior lateral line scale and ventral most lateral line scale is > 3 scales in height *Notemigonus crysoleucas*

5b. (4b) Abdomen behind pelvic fin with scales; lateral line not greatly decurved, lateral line descends < 3 scales ventrally from highest point 6

6a. (5b) With maxillary barbels, might be small and not observable without opening the mouth or with magnification 7

6b. (5b) Without maxillary barbels 8

7a. (6a) Mouth terminal; distinct black spot located anteriorly on dorsal fin; pharyngeal teeth on main row 5-4 or 5-5 *Semotilus atromaculatus*

7b. (6a) Mouth subterminal or inferior; no distinct black spot on dorsal fin; pharyngeal teeth on main row 4-4 *Macrhybopsis hyostoma*

8a. (6b) Thick lower lip at corners, mouth noticeably ventral; black spot at base of caudal fin *Phenacobius mirabilis*

8b. (6b) Lower lip thin or not noticeably thick; with or without black spot at base of caudal fin 9

9a. (8b) Predorsal scales appear crowded, smaller than scales on lateral body or appear as

overlapping scales; black spot in the middle, anterior portion of the dorsal fin 10

9b. (8b) Predorsal scales not crowded; without black spot in the middle, anterior portion of

the dorsal fin 11

10a. (9a) Caudal spot, if distinct, continuous with mid-lateral stripe; lateral line incomplete; intestine long, more than twice the standard length *Pimephales promelas*

10b. (9a) Caudal spot distinct from mid-lateral stripe; lateral line complete, intestine forming a short S-shaped loop *Pimephales vigilax*

11a. (9b) Long intestine in a flat coil *Hybognathus nuchalis*

11b. (9b) Short S-shaped intestine 12

12a. (11b) Moderately decurved lateral line; diamond-shaped scales; dark shoulder patch present; melanophores concentrated between rays of dorsal and anal fins 13

12b. (11b) Lateral line incomplete, complete-straight, or complete-slightly decurved; scales not noticeably diamond-shaped; without dark shoulder patch; melanophores concentrated along rays of dorsal and anal fins 14

13a. (12a) Sub-terminal mouth; caudal fin base with a caudal spot larger than eye; no shoulder patch *Cyprinella venusta*

13b. (12a) Terminal mouth; no caudal spot *Cyprinella lutrensis*

14a. (12b) Distinct and separate black dash at base of dorsal fin; apparent when viewed from above; pharyngeal teeth count usually 0,4-4,0 15

14b. (12b) No distinct and separate black dash at base of dorsal fin; pharyngeal teeth count usually 1,4-4,1 or 2,4-4,2 18

15a. (14a) Lateral line scales markedly elevated (taller than wide) anteriorly, elevated scale height 2 to 5 times scale width 16

15b. (14a) Lateral line scales not markedly elevated anteriorly, scale height 1 to 2 times scale width 17

16a. (15a) Dorsal and lateral body with melanophores outlining scales; with pronounced black lateral stripe; dorsal fin height goes 2.1 or more times in pre-dorsal length; infraorbital canal complete *Notropis volucellus*

16b. (15a) Dorsal and lateral body with sparse melanophores; scales outlined with melanophores are rare; pre-dorsal black spot is prominent and distinct from mid-dorsal stripe; dorsal fin height goes 2.0 or fewer times in pre-dorsal length; infraorbital canal incomplete *Notropis buchanani*

17a. (15b) Eye large, eye diameter is > snout length *Notropis stramineus*

17b. (15b) Eye small, eye diameter is < snout length *Notropis sabinae*

18a. (14b) Depressed dorsal fin longer than head. *Hybopsis amnis*

18b. (14b) Depressed dorsal fin shorter than head 19

19a. (18b) Mouth is sub-terminal; pharyngeal teeth are 0,4-4,0 *Notropis atrocaudalis*

19b. (18b) Mouth is terminal; pharyngeal teeth are 1,4-4,1 or 2,4-4,2 or 5-5 20

20a. (19b) Dorsal fin origin opposite or anterior to pelvic fin origin 21

20b. (19b) Dorsal fin origin posterior to pelvic fin origin 25

21a. (20a) Prominent mid-lateral stripe, extending through eye 22

21b. (20a) No prominent mid-lateral stripe present 24

22a. (21a) Pharyngeal teeth 5-5; mouth small and almost vertical *Opsopoeodus emiliae*

22b. (21a) Pharyngeal teeth are 1,4-4,1 or 2,4-4,2; mouth large and oblique 23

23a. (22b) Usually 8 anal fin rays; inside of mouth with black melanophores; dorsal fin insertion is opposite to pelvic fin insertion *Notropis chalybaeus*

23b. (22b) Usually 7 anal fin rays; inside of mouth without black melanophores; dorsal fin insertion is anterior to pelvic fin insertion *Notropis texanus*

24a. (21b) Usually 8 anal fin rays; head is narrow, depth at occiput more than width at occiput *Notropis shumardi*

24b. (21b) Usually 7 anal fin rays; head is wide, depth at occiput less than or equal to width at

occiput *Notropis potteri*

25a. (20b) Moderate-sized scales, ≤ 40 lateral line scales, < 24 predorsal scales *Notropis atherinoides*

25b. (20b) Small scales, ≥ 41 lateral line scales, > 25 predorsal scales 26

26a. (25b) Dorsal fin with black melanophores extending from mid-dorsal stripe into the anterior fin ray; edges of the anterior dorsolateral scales are outlined with black melanophores, producing

chevron pattern. *Lythrurus umbratilis*

26b. (25b) Dorsal fin with black melanophores not extending from mid-dorsal stripe into the anterior fin ray, lacks chevron pattern *Lythrurus fumeus*

FAMILY CATOSTOMIDAE—suckers

1a. Dorsal fin long, base > than ⅓ of standard length; 22 to 30 dorsal fin rays 2

1b. Dorsal fin short, base < than ¼ of standard length; 4 to 18 dorsal fin rays 4

2a. (1a) Small scales, lateral line scales > 50; eye closer to back of head than to tip of snout;

head abruptly more slender than body; papillose lips *Cycleptus elongatus*

2b. (1a) Large scales, lateral line scales < 45; eye closer to tip of snout than back of head; plicate lips 3

3a. (2b) Subopercle triangular, broadest toward base; knob present at tip of lower lip; blunt snout, forming level with eye *Carpiodes carpio*

3b. (2b) Subopercle semicircular, broadest towards middle; knob absent at tip of lower lip; rounded snout, forming below level of eye *Ictiobus bubalus*

4a. (1b) Lateral line complete and well developed; air bladder with 3 chambers *Moxostoma poecilurum*

4b. (1b) Lateral line incomplete or absent; air bladder with 2 chambers 5

5a. (4b) Lateral line incomplete; rows of spots *Minytrema melanops*

5b. (4b) Lateral line absent 6

6a. (5b) Scales larger, lateral scale count 34 to 37; eye larger, eye length ½ of snout length; dorsal fin rays 11 or 12; back with crescentic scale marks *Erimyzon sucetta*

6b. (5b) Scales smaller, lateral scale count 39 to 43; eye smaller, eye length < ½ of snout length); dorsal fin rays 9 or 10; back without crescentic scale marks *Erimyzon claviformis*

FAMILY CHARACIDAE—characins

*Astyanax mexicanus*

FAMILY ICTALURIDAE—bullhead catfishes

1a. Adipose fin joined to the caudal fin or separated by a shallow notch 2

1b. Adipose fin free at tip, not joined to caudal fin 3

2a. (1a) Mouth terminal; pectoral fin spine not serrated; lower lip and chin not heavily speckled with black pigment *Noturus gyrinus*

2b. (1a) Mouth sub-terminal; pectoral spine serrated; lower lip and chin heavily speckled with black pigment. *Noturus nocturnus*

3a. (1b) Head dorso-ventrally compressed; mouth terminal to superior *Pylodictis olivaris*

3b. (1b) Head rounded; mouth subterminal 4

4a. (3b) Caudal fin rounded or shallowly emarginate 5

4b. (3b) Caudal fin deeply forked 6

5a. (4a) Chin barbels completely or partially black; anal fin rays 17 to 24; anal fin broadly rounded *Ameiurus melas*

5b. (4a) Chin barbels white or yellow; anal fin rays 24 to 27; margin of anal fin generally straight *Ameiurus natalis*

6a. (4b) Anal fin rays 30 to 36; anal fin free margin is straight; medial keel-like ridge anterior to dorsal fin forms humped back appearance *Ictalurus furcatus*

6b. (4b) Anal fin rays 22 to 29; anal fin free margin is rounded; no humped back appearance *Ictalurus punctatus*

FAMILY LORICARIIDAE—suckermouth catfishes

1a. Light spots on a dark background *Pterygoplichthys anisitsi*

1b. Dark spots on a light background *Pterygoplichthys disjunctivus*

FAMILY SALMONIDAE—salmons

*Oncorhynchus mykiss*

FAMILY ESOCIDAE—pikes and pickerels

*Esox americanus*

FAMILY APHREDODERIDAE—pirate perch

*Aphredoderus sayanus*

FAMILY MUGILIDAE—mullets

1a. Lower jaw rounded, without a symphyseal knob; lower limb of 1st gill arch with 17 to 20 gill rakers; no adipose eyelid; scales ctenoid *Agonostomus monticola*

1b. Lower jaw angular, with a prominent symphyseal knob; lower limb of 1st gill arch with 25

to 60 gill rakers; adipose eyelid well developed in adults; scales cycloid in young, ctenoid in adults. *Mugil cephalus*

FAMILY ATHERINOPSIDAE—New World silversides

1a. Scales small, > 60 scales in lateral series, jaws produced into a short beak; snout length > eye length; > 20 anal fin rays *Labidesthes sicculus*

1b. Scales large, < 50 scales in lateral series; jaws not produced into a beak; snout length ≤ eye length; < 20 anal fin rays 2

2a. (1b) Scales ctenoid, rough to the touch; double pairs of black spots on dorsum; bases of dorsal and anal fin covered with scales *Membras martinica*

2b. (1b) Scales cycloid, smooth to the touch; dorsum with crosshatching, but not double pairs of black spots; bases of dorsal and anal fins not covered with scales; horizontal distance between spinous dorsal and anal fin origin less than 7% of standard length *Menidia audens*

FAMILY FUNDULIDAE—topminnows

1a. More than 40 longitudinal scale rows; dark vertical barring; gill slit not extending dorsal to uppermost pectoral fin ray *Fundulus zebrinus*

1b. Fewer than 40 longitudinal scale rows; gill slit extending dorsal to uppermost pectoral fin ray 2

2a. (1b) Body with a distinct black lateral band 3

2b. (1b) Body without a distinct black lateral band 4

3a. (2a) Distinct black spots on anterior dorso-lateral region are as pronounced as lateral stripe; distinct black spots throughout dorsal and caudal fins *Fundulus olivaceus*

3b. (2a) Faint black spots on anterior dorso-lateral region are not as pronounced as lateral stripe; distinct black spots near base of dorsal and caudal fins *Fundulus notatus*

4a. (2b) Red to dark spots in multiple rows longitudinally along lateral sides; usually with dark subocular bar *Fundulus blairae*

4b. (2b) Body mottled, barred or irregularly spotted; no dark subocular bar *Fundulus chrysotus*

FAMILY CYPRINODONTIDAE— pupfishes

*Cyprinodon variegatus*

FAMILY POECILIIDAE—livebearers

1a. Origin of dorsal fin anterior to anal fin origin; intestinal canal long with many convolutions *Poecilia latipinna*

1b. Origin of dorsal fin posterior to anal fin origin; intestinal canal short with few convolutions

*Gambusia affinis*

FAMILY MORONIDAE—temperate basses

1a. Dorsal fins united at base; 2nd and 3rd anal fin spines approximately equal in length; no teeth on tongue; 9 to 10 anal fin soft rays; stripes along sides usually sharply broken and offset above front of anal fin *Morone mississippiensis*

1b. Dorsal fins separated; 2nd anal fin spine < 3rd; base of tongue with teeth; 11 to 13 anal fin soft rays; stripes along sides usually continuous 2

2a. (1b) Body depth goes < 3 times in standard length; teeth in single patch on back of

tongue *Morone chrysops*

2b. (1b) Body depth goes > 3 times in standard length; teeth in 2 parallel patches on back

of tongue *Morone saxatilis*

FAMILY CENTRARCHIDAE—sunfishes

1a. Five to 8 anal spines 2

1b. Three anal spines 4

2a. (1a) Eleven to 13 dorsal fin spines *Centrarchus macropterus*

2b. (1a) Six to 8 dorsal fin spines 3

3a3a. (2b) Dorsal fin set back on body, length of dorsal fin base < distance from its origin to posterior margin of eye; lateral body with wide to narrow dorsal black bands; ≤ 6 dorsal spines *Pomoxis annularis*

3b. (2b) Dorsal fin set forward on body, length of dorsal fin base equal to or greater than distance from its origin to posterior margin of eye; lateral body with checkerboard black and light pattern; ≥7 dorsal spines *Pomoxis nigromaculatus*

4a. (1b) Body slender, body depth contained > 3 times into standard length 5

4b. (1b) Body deep, body depth contained < 3 times into standard length 6

5a. (4a) Dorsal fins narrowly joined at base forming a deep notch; upper jaw extends past posterior margin of eye in adults; mid-lateral stripe generally complete, rows of spots ventral to mid-lateral stripe faint and incomplete *Micropterus salmoides*

5b. (4a) Dorsal fins broadly joined at base forming a shallow notch; upper jaw does not reach past posterior portion of eye; bases of soft dorsal and anal fins scaled *Micropterus punctulatus*

6a. (4b) Teeth on tongue; head and opercle with 3 to 5 distinct dark and light longitudinal stripes; red spot on posterior margin of opercle flap in fresh specimens *Lepomis gulosus*

6b. (4b) No teeth on tongue; head and opercle lacking distinct dark and light longitudinal stripes 7

7a. (6b) Pectoral fins long and pointed, reach anterior portion of eye or beyond when bent forward 8

7b. (6b) Pectoral fins short and rounded, do not reach past eye when bent forward 10

8a. (7a) Opercle flap stiff to its margin, posterior margin either red or orange in live specimens *Lepomis microlophus*

8b. (7a) Opercle flap flexible, posterior margin not red or orange in live specimens 9

9a. (8b) Opercle flap black to the margin; black spot on posterior base of soft dorsal fin *Lepomis macrochirus*

9b. (8b) Opercle flap outlined with thick white band; lacking black spot on posterior base of soft dorsal fin *Lepomis humilis*

10a. (7b) Black opercle flap stiff near the posterior margin with bone supporting all or majority of the flap 11

10b. (7b) Black opercle flap flexible near the posterior margin without bone supporting majority of the flap 13

11a. (10a) Lateral line incomplete; smaller individuals with black spot surrounded by white margin on posterior base of soft dorsal fin *Lepomis symmetricus*

11b. (10a) Lateral line complete; black spot, if present, on posterior base of soft dorsal fin without white margin 12

12a. (11b) Body elongated with black spot on posterior base of soft dorsal fin *Lepomis cyanellus*

12b. (11b) Body rounded without black spot on posterior base of soft dorsal fin; lateral body with alternating stripes formed from black and red spots *Lepomis miniatus*

13a. (10b) Opercle flap black to the posterior margin; opercle flap is thin near the opercle bone with the narrowest width of the flexible portion of the flap about the same diameter of the eye pupil *Lepomis auritus*

13b. (10b) Opercle flap black and surrounded by white on the posterior margin; opercle flap is wide with narrowest width of the flexible flap is about two times the diameter of the eye pupil 14

14a. (13b) Twelve pectoral fin rays, 3 to 5 cheek scales; opercle flap often with white pigment form speckles, distinct red spots (white in preserved specimens) along lateral line *Lepomis marginatus*

14b. (13b) Thirteen to 15 pectoral fin rays, 5 to 7 cheek scales; opercle flaps with red or white margin; 13 to 15 pectoral fin rays *Lepomis megalotis*

FAMILY PERCIDAE—perches

1a. Body depth contained in standard length > 7 times *Ammocrypta vivax*

1b. Body depth contained in standard length < 7 times 2

2a. (1b) Snout conical, extends beyond upper lip; body with ≥ 14 black vertical bars 3

2b. (1b) Snout less conical, does not extend beyond upper lip; body with < 14 black vertical bars or with a pattern other than vertical bars 4

3a. (2a) Body with thick vertical bars, bars alternate in length from long to short; 9 to 10 long bars *Percina carbonaria*

3b. (2a) Body with 14 to 16 thin vertical bars of similar length *Percina macrolepida*

4a. (2b) Sides of body with large black blotches; midline of abdomen naked or with enlarged scales 5

4b. (2b) Sides of body without large black blotches; scales on abdomen normal 6

5a. (4a) Nape unscaled; blotches on sides of body are rectangle shaped and might appear connected *Percina maculata*

5b. (4a) Nape scaled; blotches on sides of body not rectangle shaped *Percina sciera*

6a. (4b) Lateral line short, < 6 pored scales; single row of horizontal dashes present *Etheostoma proeliare*

6b. (4b) Lateral line long (complete or incomplete), > 6 pored scales; if horizontal dashes present, accompanied by vertical bars 7

7a. (5b) Lateral line arched upward *Etheostoma gracile*

7b. (5b) Lateral line straight 8

8a. (6b) Lateral body with distinct series of M-shaped pigments; snout rounded and blunt *Etheostoma chlorosoma*

8b. (6b) Lateral body without distinct series of M-shaped pigments; snout not noticeably rounded and blunt 9

9a. (8b) Lateral region with mottling bisected by a light colored lateral stripe *Etheostoma parvipinne*

9b. (8b) Lateral region without mottling bisected by a light colored lateral stripe; lateral region with a series of dashes followed by a series of vertical bars; throat of live males orange *Etheostoma spectabile*

FAMILY SCIAENIDAE—drums

*Aplodinotus grunniens*

FAMILY ELASSOMATIDAE—pygmy sunfishes

*Elassoma zonatum*

FAMILY CICHLIDAE—cichlids

1a. Anal fin spines 5 to 6 *Herichthys cyanoguttatus*

1b. Anal fin spines < 5 (usually 3) *Oreochromis aureus*

FAMILY ACHIRIDAE—American soles

*Trinectes maculatus*
